# Differential Impact of Tumor Suppressor Pathways on DNA Damage Response and Therapy-Induced Transformation in a Mouse Primary Cell Model

**DOI:** 10.1371/journal.pone.0008558

**Published:** 2010-01-01

**Authors:** A. Kathleen McClendon, Jeffry L. Dean, Adam Ertel, Erik S. Knudsen

**Affiliations:** Kimmel Cancer Center, Department of Cancer Biology, Thomas Jefferson University, Philadelphia, Pennsylvania, United States of America; Roswell Park Cancer Institute, United States of America

## Abstract

The RB and p53 tumor suppressors are mediators of DNA damage response, and compound inactivation of RB and p53 is a common occurrence in human cancers. Surprisingly, their cooperation in DNA damage signaling in relation to tumorigenesis and therapeutic response remains enigmatic. In the context of individuals with heritable retinoblastoma, there is a predilection for secondary tumor development, which has been associated with the use of radiation-therapy to treat the primary tumor. Furthermore, while germline mutations of the p53 gene are critical drivers for cancer predisposition syndromes, it is postulated that extrinsic stresses play a major role in promoting varying tumor spectrums and disease severities. In light of these studies, we examined the tumor suppressor functions of these proteins when challenged by exposure to therapeutic stress. To examine the cooperation of RB and p53 in tumorigenesis, and in response to therapy-induced DNA damage, a combination of genetic deletion and dominant negative strategies was employed. Results indicate that loss/inactivation of RB and p53 is not sufficient for cellular transformation. However, these proteins played distinct roles in response to therapy-induced DNA damage and subsequent tumorigenesis. Specifically, RB status was critical for cellular response to damage and senescence, irrespective of p53 function. Loss of RB resulted in a dramatic evolution of gene expression as a result of alterations in epigenetic programming. Critically, the observed changes in gene expression have been specifically associated with tumorigenesis, and RB-deficient, recurred cells displayed oncogenic characteristics, as well as increased resistance to subsequent challenge with discrete therapeutic agents. Taken together, these findings indicate that tumor suppressor functions of RB and p53 are particularly manifest when challenged by cellular stress. In the face of such challenge, RB is a critical suppressor of tumorigenesis beyond p53, and RB-deficiency could promote significant cellular evolution, ultimately contributing to a more aggressive disease.

## Introduction

The response to genotoxic stress is a critical event that has broad implications to cancer. It is well appreciated that a number of environmental carcinogens act through the induction of DNA damage to promote tumor initiation [1], [2]. For example, Aflatoxin B1 elicits oxidative damage and is a key etiological factor for hepatocellular carcinoma [3], and exposure to solar radiation is a key risk factor for skin cancer [4]. While genotoxic agents are strongly linked to tumorigenesis, the cytotoxic effect of DNA damage is also a critical facet of cancer therapy. In fact, the majority of human tumors are treated using agents that are genotoxic compounds. A major caveat of such therapies is the possibility of inducing secondary primary malignancies, or exacerbating existing disease by promoting genomic instability or facilitating selection of aggressive, therapy-resistant forms of disease [5]. Clearly, understanding genetic alterations that influence these responses is critical for efficacious cancer treatment.

The retinoblastoma tumor suppressor (RB) is a regulator of the cell cycle that is functionally inactivated in a variety of human cancers [6], [7], [8]. RB functions as a negative regulator of a transcriptional program that is mediated by E2F transcription factors [9], [10]. Transcriptional targets of RB include genes involved in diverse processes, including DNA replication, cell cycle progression, DNA damage response, and apoptosis [11], [12], [13]. Correspondingly, the deletion of RB leads to the deregulation of these target genes in many model systems [14].

An important consequence of gene deregulation through RB loss is the propensity to facilitate bypass of the canonical DNA damage checkpoints that inhibit G1 and S-phase progression [15], [16]. This function of RB is similar to that of the p53 tumor suppressor [17], [18], [19], [20]. While there is evidence that RB and p53 function in related/partially overlapping pathways to modify cell cycle checkpoints, this point remains unresolved and is likely modified by discrete forms of DNA damage. Importantly, many tumors display disruption of both tumor suppressor pathways, suggesting intrinsic cooperation [21], [22], [23]. One basis for this cooperation is that while RB deficiency is associated with enhanced cell death, p53 deficiency facilitates cell survival [17], [18], [19], [24].

How RB and p53 cooperate in DNA damage signaling in relation to tumorigenesis and therapeutic response is not completely understood. In breast, lung, and prostate cancer models, RB deficiency was associated with enhanced sensitivity to cytotoxic therapy [8], [25], [26], [27]. However, increased sensitivity in such models did not lead to durable response, and recurrence can contribute to therapy-resistance. In the context of individuals with heritable retinoblastoma, there is a strong predilection for secondary tumor development [28], [29], [30]. Particularly, such secondary tumor development has been closely associated with the use of radiation-therapy to treat the primary retinoblastoma [28], [29], [31]. Similarly, while germline mutations of p53 are major drivers for cancer predisposition syndromes, identical mutations have been shown to result in widely varying tumor spectrums and severities in different patients [32]. It is postulated that extrinsic stresses (either environmental or therapy-induced) play a major role in promoting secondary “hits” that can lead to higher cancer predisposition [33], [34].

In light of these studies, we questioned whether the combined loss of RB and p53 is sufficient to drive tumorigenesis, or if the tumor suppressor functions of these proteins are only truly manifest when challenged by exposure to therapeutic stress. Our findings indicate that loss of RB and p53 alone is not sufficient for transformation. However, these proteins played distinct roles in the response to therapy-induced damage, and interestingly, RB was observed to be a critical modulator of DNA damage response and senescence, irrespective of p53 status. Consequently, RB deficiency promoted significant cellular evolution, ultimately resulting in tumorigenesis and enhanced therapeutic resistance.

## Materials and Methods

### Isolation of Primary Rb^loxP/loxP^ Mouse Adult Fibroblasts (MAFs) and Cell Culture

Primary mouse adult fibroblasts (MAFs) were isolated from floxed Rb (Rb^loxP/loxP^) mice and cultured as previously described [16]. Rb^loxP/loxP^ MAFs were then subcultured in DMEM containing 10% fetal bovine serum supplemented with 100 U/mL penicillin/streptomycin and 2 mM L-glutamine at 37°C in 5% CO_2_. All animal experiments were conducted in accordance with the NIH Guide for Care and Use of Laboratory Animals and were approved by the Thomas Jefferson University Institutional Animal Care and Use Committee.

### Viral Infections

Cells were infected with adenoviral constructs expressing green fluorescent protein (GFP) or GFP and Cre recombinase (GFP-Cre), with an infection efficiency of 90–95% as determined by GFP immunofluorescence. Recombination at the Rb locus of Rb^loxP/loxP^ MAFs was confirmed by PCR as previously described [35]. GFP MAFs and GFP-Cre MAFs were infected with retrovirus encoding LXSN or LXSN-p53DD, selected, and pooled for characterization as previously described [26].

### Immunoblot Analysis

Total cell lysates were resolved by SDS-PAGE and transferred to Immobilon-P membrane (Millipore, Bedford, MA). Proteins were detected using the following antibodies: from Santa Cruz Biotechnology- lamin B (M-20), MCM7 (141.2), Cyclin A (C-19), PCNA (PC10), p21 (C-19), phospho-ERK (E-4), ERK (K-23); from Novacastra Ltd.- p53 (CM5); from Neomarkers- Cyclin D1 (Ab-3).

### Cell Growth Analysis

1.5×10^5^ cells were seeded (Day 0), and every 24 h, cell numbers were counted using trypan blue exclusion. For cisplatin (CDDP) treatments, cells were treated 24 h after seeding, and plates were stained with 1% crystal violet at the indicated time points. Anchorage independent growth was facilitated through plating cells into non-coated Petri dishes. For 5-aza-2′-deoxycytidine (5-aza-dC) (Sigma-Aldrich, St. Louis, MO) treatments, cells were initially treated with 5 µM 5-aza-dC for 24 h, then subsequently treated for 5 days with 1 µM 5-aza-dC.

### BrdU Incorporation/Flow Cytometry

Cells were treated with CDDP (4 and 8 uM), Etoposide (1 uM), Camptothecin (5 uM), or Mitomycin C (2 uM) for 24 h, or CDDP (0.5 uM) up to 96 h, labeled with BrdU for 1 h, and processed for flow cytometry as previously described [35].

### β-galactosidase Assay

Cells were treated with CDDP (0.5 uM) for 48 h, and stained for β-galactosidase (β-gal) activity using a Senescence β-gal Staining Kit (Cell Signaling Technology, Danvers, MA). β-gal-positive cells were quantified and displayed as fold-increase in expression over untreated cells.

### Foci Formation

Cells were treated with CDDP (1 uM) for 24 h, allowed to recover in fresh media lacking CDDP, and stained with 1% crystal violet two weeks post-treatment. Foci formation was quantified by measuring the relative intensity of staining using ImageJ 1.41 software. Foci formation recovery assays were preformed with six independently treated cell populations.

### Microarray Analysis

RNA was harvested using TRIzol (Invitrogen, Carlsbad, CA) according to the manufacturer's suggested protocols and quantified on a Nanodrop ND-100 spectrophotometer. RNA quality was assessed by analysis on an Agilent 2100 bioanalyzer (Agilent, Palo Alto, CA). Two micrograms of total RNA from each cell type was used for Affymetrix one-cycle target labeling as recommended by the manufacturer (Affymetrix, Santa Clara, CA). Each Affymetrix GeneChip for Mouse Genome 430 2.0 was hybridized for 16 h with biotin-labeled fragmented cRNA (10 µg) in 200 µL of hybridization cocktail according to Affymetrix protocol. Arrays were washed and stained using GeneChip Fluidic Station 450, and hybridization signals were amplified using antibody amplification with goat IgG (Sigma-Aldrich, St. Louis, MO) and anit-streptavidin biotinylated antibody. Chips were scanned on an Affymetrix GeneChip Scanner 3000 using GeneChip Operating Software verson 3.0. Microarray normalization was performed by computing the Robust Multichip Average (RMA) expression measure [PMID: 12582260]. Significant genes changes were determined using Significance Analysis of Micorarrays (SAM) [PMID: 11309499] in the TM4 MultiExperiment Viewer (MeV) software package [PMID: 12613259]. Significant changes in gene expression were determined using a 1.5-fold cut-off in expression change and a FDR of <25%. Microarray analyses were performed on at least two independently generated (naïve or recurred) cell populations. The complete microarray gene list is included in Table S1. All microarray data are MIAME compliant and have been deposited in NCBI's Gene Expression Omnibus (GEO). Raw data is accessible through GEO Series accession number GSE18395.

Chromosome locations for the Mouse Genome 430 2.0 probe sets were extracted from the Mouse430_2 annotation file release 29, dated 7/1/2009, available from the Affymetrix website. Chromosome locations and fold-change values for the 1176 significant probe sets (<25% FDR and 1.5 fold-change) were imported into Matlab and log2 fold-change was plotted as a function of chromosome position.

### RT-PCR

RNA was harvested from cell cultures using TRIzol (Invitrogen, Carlsbad, CA) according to the manufacturer's suggested protocol. Superscript reverse transcriptase (Invitrogen, Carlsbad, CA) and 5 g of total RNA were used to generate cDNAs with random hexamer primers. PCR was carried out using the following primers: 5′-GTCACAGACCTGCAGTGGCTCAT-3′ and 5′-ATCTTGGAGTAAGACAGTGGTCC-3′ (Lumican), 5′-GCCACAACGTGGGCTACAA-3′ and 5′-ACCTCTGCCATGGTCTCGTG-3′ (Sfrp1), 5′-ATGGAAACCCTTTGTAAAAATGACT-3′ and 5′-TCTTGCTCTTTGTCTCCAGGATGAT-3′ (Sfrp2), 5′-GAAGGGCCAGTGTGAAAGTG-3′ and 5′-GGGCAGGATTGTTGGTTGAA-3′ (Clusterin), 5′-GTTGCCTTCGATGGGAAAAAG-3′ and 5′-TGGGACAGTCGTCTCTCTGGA-3′ (Gpr149), 5′-CAGACTCCAAAGGACATCGCAA-3′ and 5′-GTCAAAGGGTGACCCAGGAA-3′ (Pla2g7), 5′-ATCTTCCAGGAGCGAGACCCCA-3′ and 5′-TCCACAATGCCAAAGTTGTCATGG-3′ (GAPDH). PCR conditions were as follows: denaturation at 94°C for 30 s, annealing at 58°C for 30 s, and elongation at 72°C for 30 s for all reactions, with 30 cycles for SFRP1, Gpr149 and GAPDH, and 36 cycles for Lumican, SFRP2, Clusterin and Pla2g7.

### Immunofluorescence

For phalloidin staining, cells were plated on coverslips in 6-well dishes, fixed in 3.7% formaldehyde, and processed according to the manufacturer's protocol (Invitrogen Molecular Probes, Carlsbad, CA). For CDDP adduct detection, cells were plated on coverslips, treated with 8 µM CDDP for 24 h, fixed in 3.7% formaldehyde, and processed as previously described [36].

### Xenografts

Tumors were grown as xenografts in 6-week-old to 8-week-old female athymic nude mice (Harlan Sprague-Dawley, Inc.) by subcutaneous flank injection of 2×10^6^ cells in 150 µL of phosphate-buffered saline solution mixed with 50 µL of Matrigel (BD Biosciences, Bedford, MA). Once palpable, tumors were measured with calipers every 7 days, and tumor volume was calculated as *v = π(width^2^×length)/6*. Xenograft tumor formation assays were performed in two cohorts of nude mice with two independently generated (naïve or recurred) cell populations. All animal experiments were conducted in accordance with the NIH Guide for Care and Use of Laboratory Animals and were approved by the Thomas Jefferson University Institutional Animal Care and Use Committee.

### H&E Tissue Staining

Tissue from xenografts was fixed in 10% neutral buffered formalin (NBF), paraffin-embedded, and cut into 5 um sections. Sections were stained with H&E using standard techniques.

### Statistical Analysis

All statistical analyses were performed with GraphPad Prism (GraphPad Prism Software, Inc.) unless otherwise indicated. *P* values were calculated by performing Student's T-Tests.

## Results

### RB Mediates Acute Cell Cycle Arrest in Response to Therapy, Independent of p53 Status

**Figure 1:**
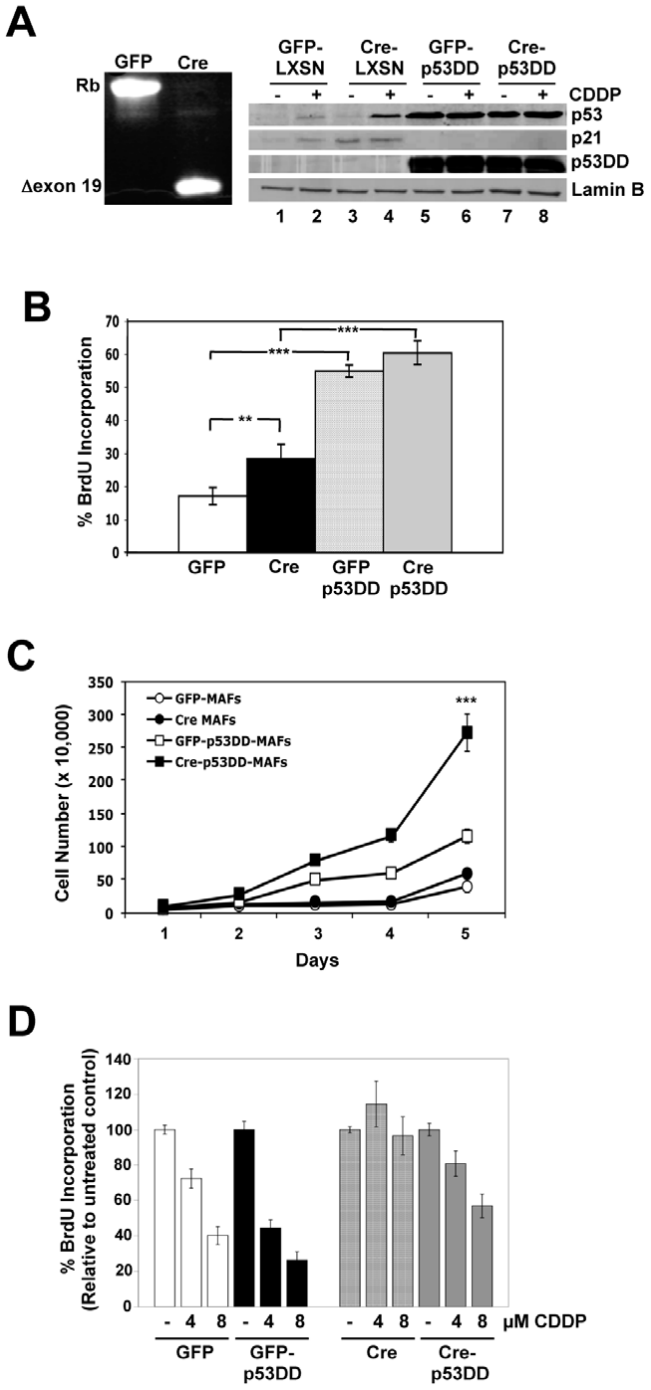
Characterization of RB/p53 cooperation in cell growth and acute DNA damage response. (A) Mouse adult fibroblasts isolated from Rb^loxP/loxP^ mice were infected with adenoviral GFP or GFP-Cre. Recombination was examined by PCR using primer sequences flanking *Rb* exon 19. MAFs were subsequently infected with retroviruses encoding LXSN or LXSN-p53DD. Cells were treated with 8 µM CDDP for 24 h, and total protein lysates were immunoblotted for p53 and p21. Lamin B served as a loading control. (B) Cells were cultured in normal growth media, pulsed with BrdU for 1 h prior to harvesting, stained with FITC-anti-BrdU and PI and analyzed by flow cytometry. Average percent BrdU incorporation is shown. **p<0.0054, ***p<0.0002 (C) Cells were cultured in normal growth media and viable cells were counted every 24 h. ***p<0.0001 (D) Cells were treated with 4 or 8 µM CDDP for 24 h, pulsed with BrdU for 1 h prior to harvesting, stained with FITC-anti-BrdU and PI, and analyzed by flow cytometry. Average percent BrdU incorporation relative to untreated controls is shown. ***p<0.0001.

To examine the cooperation of RB and p53 dysfunction in response to therapy-induced damage, primary adult fibroblasts (MAFs) were isolated from Rb^loxp/loxp^ mice and subjected to viral transduction targeting the *Rb* gene and/or the p53 protein. Specifically, MAFs were infected with adenovirus expressing either a GFP vector or GFP and Cre recombinase, which results in the deletion of exon 19 of *Rb* (Figure 1A, left panel). This recombination event has been established to result in the loss of RB protein expression [35], [37]. Additionally, RB-proficient and RB-deficient MAFs were infected with an empty retroviral vector, LXSN, or a retroviral vector expressing an N-terminally truncated dominant-negative form of murine p53, LXSN-p53DD (Figure 1A, right panel). Similar to previous reports, cells infected with LXSN-p53DD displayed an accumulation of endogenous p53 (Figure 1A, right panel, lanes 5–8). However, elevated levels of endogenous p53 did not result in up-regulation of p21 in the presence of the DNA damaging compound cisplatin (CDDP) (Figure 1A, right panel, compare lanes 2 and 4 to lanes 6 and 8), indicating that the response of the p53 pathway to DNA damage had been compromised [26], [38]. Introduction of LXSN-p53DD into MAFs resulted in increased levels of DNA replication (Figure 1B), as well as enhanced cell proliferation (Figure 1C) when compared to control cell populations. Interestingly, RB and p53 deficiency resulted in additive effects on proliferation in extended culture, resulting in a >2-fold growth advantage as compared to cells harboring a single tumor suppressor deficiency (Figure 1C).

Previous studies have shown that loss of RB or p53 results in bypass of the G_1_/S checkpoint normally triggered by DNA damage [15], [16]. To examine the cooperation of RB and p53 deficiency in bypass of therapy-induced damage, cells were challenged with cisplatin (CDDP) for 24 hours, and DNA replication was monitored by BrdU incorporation (Figure 1D). RB-proficient cells displayed a significant decrease in DNA replication upon exposure to CDDP, independent of p53 status. In contrast, loss of RB resulted in bypass of DNA damage-mediated cell cycle arrest, though some sensitivity is observed at higher concentrations (Figure 1D). These data suggest that while both the RB and p53 pathways play a role in regulating normal cell proliferation, RB function is dominant for cell cycle checkpoint response to therapy-induced damage.

### RB Status Is Critical for Maintaining Cell Cycle Arrest and Initiating Cellular Senescence in the Absence of a Functional p53 Pathway

**Figure 2:**
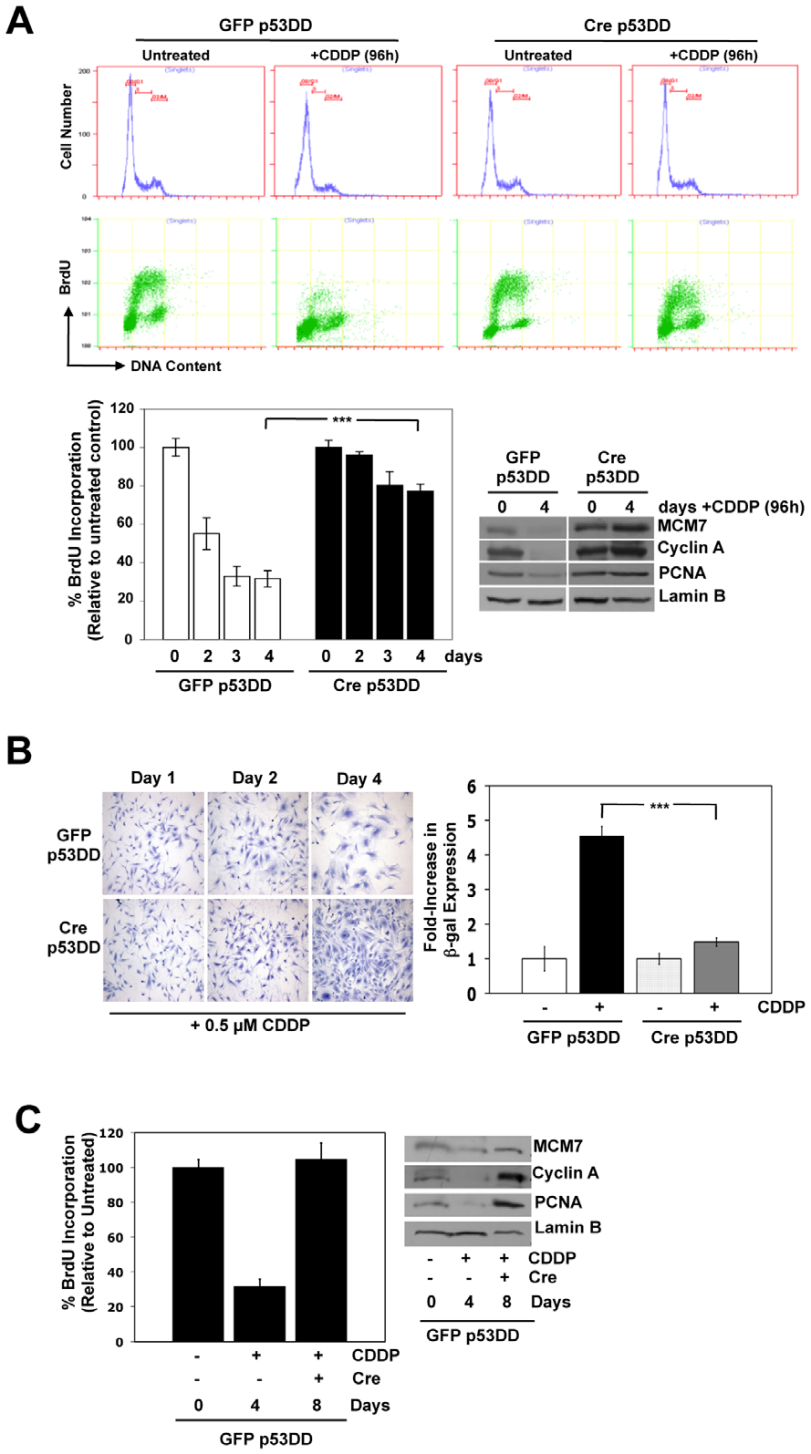
RB status mediates cell growth vs. senescence in response to chronic therapy-induced damage, independent of p53 status. (A) Cells were treated with 0.5 µM CDDP for 4 days. Cells were treated with 0.5 µM CDDP for 24 h, pulsed with BrdU for 1 h prior to harvesting, stained with FITC-anti-BrdU and PI, and analyzed by flow cytometry. *Top*, representative traces for both PI and BrdU staining are shown. *Left*, average percent BrdU incorporation relative to untreated controls is shown. ***p<0.0001 *Right*, total protein lysates were immunoblotted for the indicated proteins. Lamin B served as a loading control. (B) *Left*, cells were cultured in 0.5 µM CDDP, and viable cells were stained with crystal violet. *Right*, cells were treated with 0.5 µM CDDP for 2 days and stained for β-gal activity. Fold-increase in β-gal activity relative to untreated controls is shown. ***p<0.0001 (C) Four days post-CDDP exposure, RB-proficient cells were subjected to adenoviral infection to delete *Rb*. *Left*, four days post-infection, cells were pulsed with BrdU for 1 h prior to harvesting and processed as described in (A). *Right*, total protein lysates were prepared at each time point for immunoblot analysis. Lamin B served as a loading control.

To mimic clinically relevant exposure to therapies, such as CDDP, we next monitored the ability of the cells to proliferate under continuous, low-dose (0.5 uM) CDDP treatment. Levels of DNA replication in RB-proficient p53DD cells decreased by ∼70% in the presence CDDP over the course of 4 days (Figure 2A, top and left panel). In contrast, levels of DNA replication were diminished by only ∼20% in RB-deficient p53DD cells, confirming the importance of RB in not only initiating cell cycle checkpoints while p53 is disabled, but also in maintaining cell cycle arrest. Concomitantly, cell cycle and replication proteins such as Cyclin A, MCM7 and PCNA are nearly undetectable in RB-proficient cells after a prolonged exposure to CDDP, while these same proteins are detected at equal levels in RB-deficient cells under the same treatment (Figure 2A, right panel). Furthermore, RB-proficient p53DD cells displayed a senescent-like morphology as early as 2 days after exposure to CDDP, while RB-deficient cells grew uninhibited in the presence of CDDP (Figure 2B, left panel). As a direct test of cellular senescence, RB-proficient cells displayed a 4- to 5-fold increase in β-galactosidase staining over both the untreated control cells and the RB-deficient cells in the absence or presence of CDDP (Figure 2B, right panel).

To further interrogate whether the presence of RB is responsible for the maintenance of cell cycle arrest, RB-proficient p53DD cells were exposed to CDDP for 4 days, resulting in significant cell cycle arrest and appearance of senescent cells. These cells were then infected with Cre recombinase-encoding adenoviruses to delete *Rb*. Upon *Rb* deletion, cells escaped from cell cycle arrest and began replicating at levels equivalent to the original untreated cell population (Figure 2C, left panel). Correspondingly, levels of cell cycle and replication proteins were dramatically enhanced (Figure 2C, right panel). Thus, these studies indicate that RB status is not only required for the initiation of cell cycle arrest by DNA damaging agents, but RB actively participates in the maintenance of such cell cycle withdrawal in the presence of a disabled p53 pathway.

### RB-Deficiency Promotes Aggressive Recovery from Therapy-Induced Damage and Dramatic Alterations in Gene Expression

**Figure 3:**
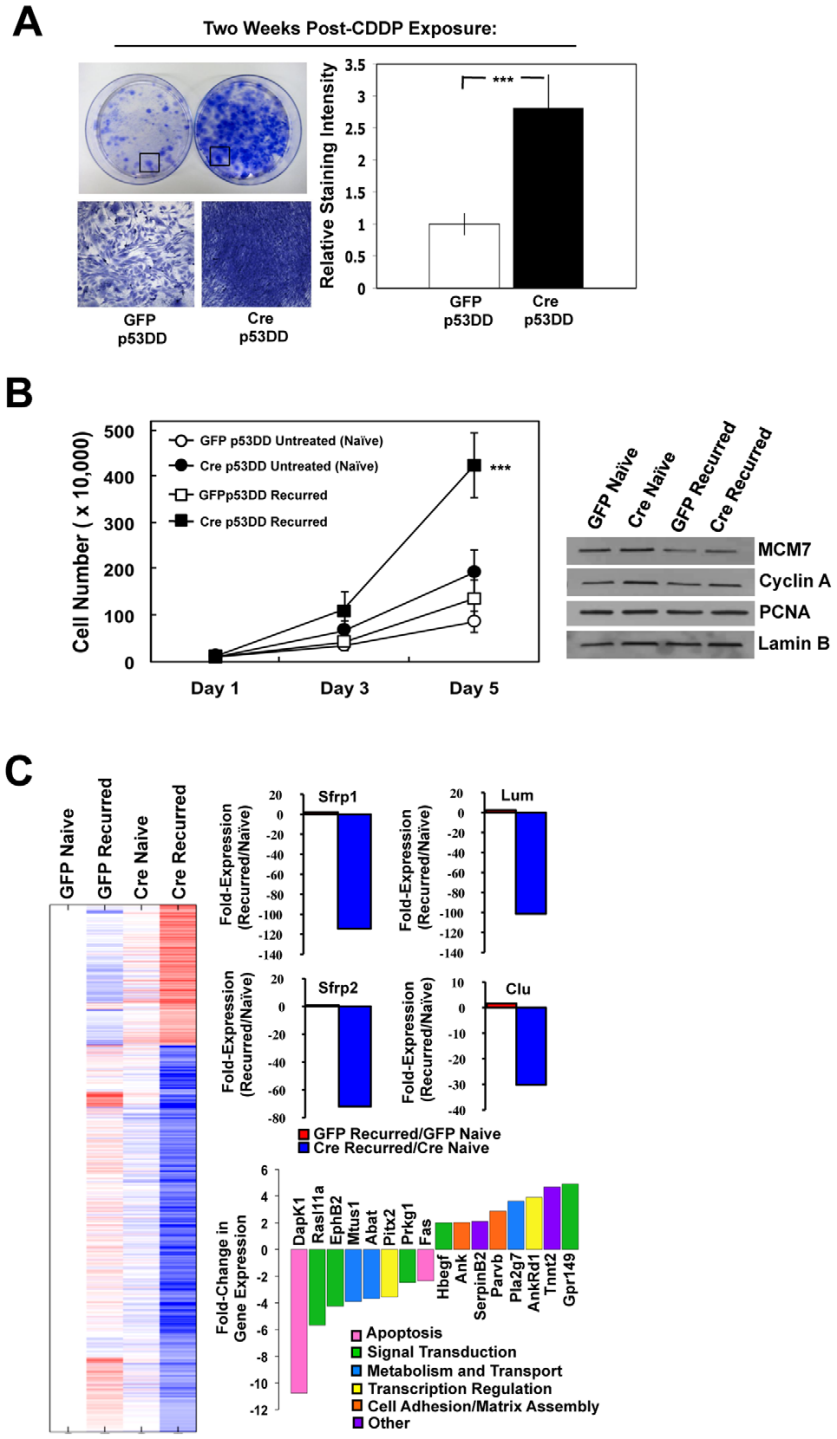
RB loss promotes aggressive recovery from therapy-induced damage and significant alterations in gene expression. (A) Cells were treated with 1 uM CDDP for 24 h, allowed to recover in fresh media, and stained with crystal violet two weeks post-treatment. Relative staining intensities from six independent experiments were quantified. ***p<0.0001 (B) *Left*, cells were cultured in normal growth media, and viable cells were counted over a course of 5 days. ***p<0.0001 *Right*, total protein lysates were immunoblotted for the indicated proteins. Lamin B served as a loading control. (C) Gene expression data was averaged for each cell line and normalized to the RB-proficient p53DD, untreated cell population (GFP Naïve). *Left*, changes in gene expression are displayed as a heat map. *Right*, those genes with specific involvement in transformation are highlighted in bar graphs. Gene expression values are displayed as fold-change in gene expression.

To evaluate the ability of cells deficient in RB and/or p53 to recover from therapy-induced damage, cells were treated with CDDP for 24 h and then allowed to recover in fresh media without CDDP. Initial recovery experiments were performed with six independently treated cell populations (Figure 3A), and all subsequent assays were preformed with a minimum of two independent, recovered cell populations. Two weeks post-CDDP exposure, RB-proficient p53DD cells slowly recovered from treatment and resumed proliferation (Figure 3A). In contrast, RB-deficient p53DD cells recovered faster and very aggressively from treatment, forming cell foci with a density ∼3-fold greater than that of the RB-proficient cells, as determined by intensity of crystal violet staining (Figure 3A). Upon further evaluation of the recurred, RB- and/or p53-deficient cell populations, the RB-deficient, recurred cells grew ∼3–4 times faster than the recurred, RB-proficient cells, and with twice the kinetics of the naïve, RB-deficient cells (Figure 3B, left panel). Interestingly, the enhanced growth rate did not correspond to increased levels of cell cycle and/or replication proteins (Figure 3B, right panel), indicating that cell characteristics beyond those directly mediated by the RB pathway may have been altered.

To define the basis of the aforementioned changes in cell growth, gene expression profiling of each cell population was determined using the Affymetrix GeneChip Mouse Genome 430 2.0 Array. Gene expression values were normalized to the RB-proficient p53DD, untreated cells (GFP Naïve), and significant changes in gene expression were determined using a 1.5-fold cut-off and a FDR of <25%. These data are displayed as a heat map in Figure 3C (left panel). Only modest changes in gene expression were observed between the RB-proficient and RB-deficient p53DD, untreated cells, which were not significant based on the aforementioned statistics. Similarly, comparison of the RB-proficient, naïve and recurred cells (GFP Recurred vs. GFP Naïve) indicated relatively modest alterations in gene expression. In contrast, the RB/p53-deficient cells that had been treated with CDDP (Cre Recurred) displayed dramatic alterations in gene expression not observed in any other cell population (Figure 3C). Gene ontology analyses did not identify a single key pathway altered in these cell populations. However, the genes that were significantly modified in this population included those previously shown to suppress transformation (Figure 3C, top right panel) [39], [40], [41], [42], [43]. Additionally, the RB-deficient p53DD, recurred cells exhibited alterations in gene expression that were in accord with a recently described signature of cooperative transformation (Figure 3C, bottom right panel) [44]. Thus, CDDP damage, in the absence of RB and p53, yielded a complex gene expression program that had hallmarks associated with a transformed/oncogenic phenotype.

### RB-Deficiency Promotes Alterations in Epigenetic Programming, Resulting in Enhanced Cell Growth

**Figure 4:**
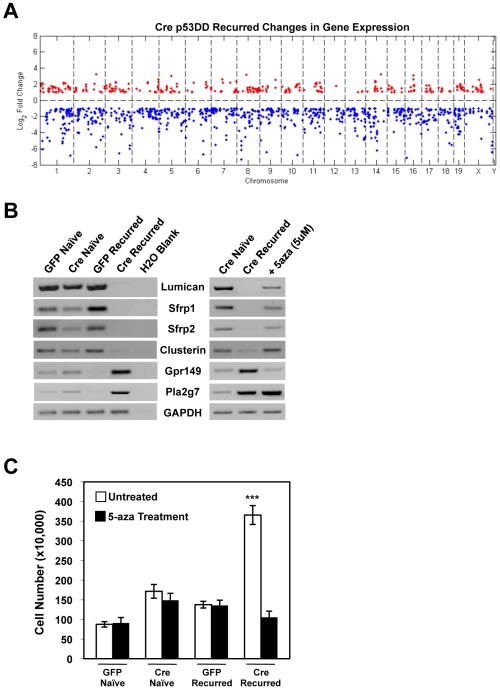
RB loss promotes alterations in epigenetic programming. (A) Gene expression data for the RB-deficient p53DD, recurred cells demonstrated by microarray analysis were mapped along the mouse genome, indicating locations of up-regulated (Red) and down-regulated (Blue) genes. (B) Cells were cultured in the absence or presence of 5-aza-dC for 5 days, and RT-PCR was carried out for the indicated genes. (C) Cells were cultured in the absence or presence of 5-aza-dC for 5 days, and viable cells were counted. ***p<0.0001.

To determine the underlying mechanism behind the dramatic alterations in gene expression observed in the RB-deficient p53DD, recurred cells, we first examined the locations of the gene changes across the mouse genome. The dramatic (>50 fold) down-regulation in specific genes displayed in the microarray data indicated potential deletions of chromosome regions. However, upon examining the overall distribution of gene changes across the mouse genome, it was apparent that the gene alterations were located throughout the genome and not the necessarily reflective of a localized event (Figure 4A). These data lead us to examine the possibility that epigenetic alterations were behind the dramatic gene down-regulation. In order to determine whether the changes in gene expression observed in the RB-deficient p53DD, recurred cells were the result of alterations in methylation, cells were treated with 5-aza-2′-deoxycytidine (5-aza-dC). First, RT-PCR was performed to validate the gene expression profiles of a panel of genes in each RB-proficient and RB-deficient, naïve and recurred cell population (Figure 4B, left panel). Then, the RB-deficient p53DD, recurred cells were treated with 5-aza-dC for 5 days, and gene expression was re-examined. In the panel of genes examined, 5-aza-dC treatment resulted in restoration of expression of those genes that were significantly down-regulated in the RB-deficient, recurred cell population (Figure 4B, right panel). Interestingly, genes that were up-regulated in the RB-deficient, recurred cells displayed varying responses to 5-aza-dC treatment. For example, while Gpr149 expression was sensitive to 5-aza-dC treatment, Pla2g7 expression was unaltered (Figure 4B, right panel). Taken together, these data indicate that, at least for these genes that were down-regulated in the RB-deficient p53DD, recurred cells, changes in epigenetic programming represent a key mechanism behind the altered gene expression profile observed.

To determine whether such epigenetic alterations were responsible for the enhanced growth characteristics of the RB-deficient p53DD, recurred cell population, cell growth was examined after 5 days of 5-aza-dC treatment. The RB-deficient, recurred cells displayed a significantly decreased growth rate as a result of 5-aza-dC treatment (Figure 4C). Furthermore, the RB-proficient, naïve and recurred cells, as well as the RB-deficient, naïve cells, were unaffected by 5-aza-dC treatment. These data indicated that the enhanced growth characteristics observed in the RB-deficient, recurred cells were the result of a dramatic deregulation of gene expression caused by alterations in epigenetic programming.

### RB-Deficiency and Response to Therapy-Induced Damage Results in a Tumorigenic Cell Population

**Figure 5:**
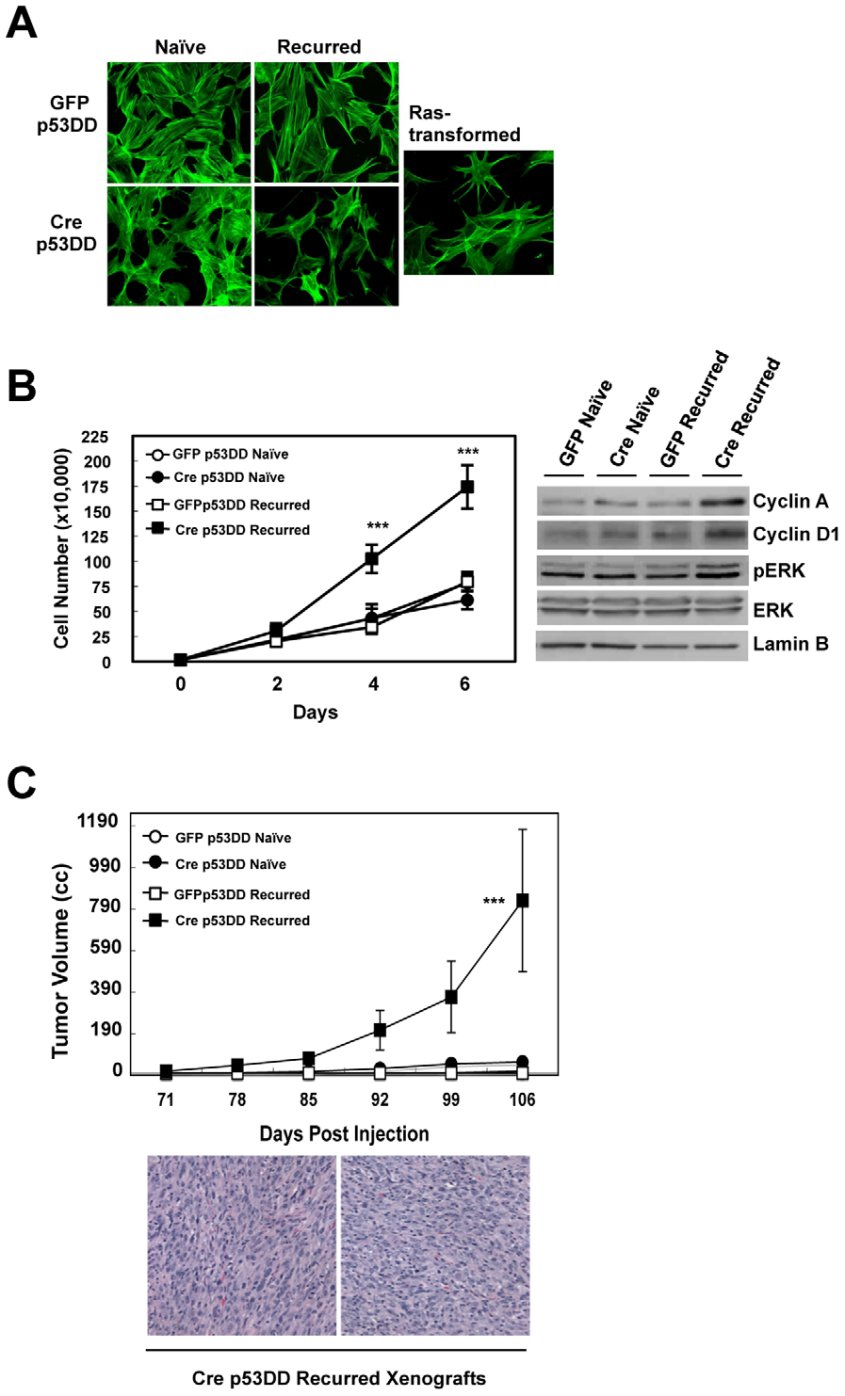
RB-deficiency and response to therapy-induced damage promotes tumorigenesis. (A) Asynchronously growing cells were stained with phalloidin. Ras transformed cells are representative of transformed morphology. (B) *Left*, cells were cultured in non-coated Petri dishes, and viable cells were counted over a course of 6 days. ***p<0.0001 *Right*, total protein lysates were immunoblotted for the indicated proteins. Lamin B served as a loading control. (C) *Top*, Cells were injected into the flanks of nude mice, and tumor volumes were measured every 7 days post-palpable tumor formation. ***p = 0.0003 *Bottom*, representative H&E stained tissue sections of Cre p53DD, recurred xenografts are shown.

Based on these findings, we directly interrogated the tumorigenic properties of both naïve and recurred cell populations. Initially, the overall morphology of the cells, as determined by phalloidin staining, indicated significant remodeling of actin fibers, similar to that observed in Ras-transformed cells (Figure 5A). To further assess the potential transformation of these cells, RB-proficient and RB-deficient p53DD, naïve and recurred cells were challenged first by growth under anchorage independent conditions. The RB-deficient p53DD, recurred cells displayed a significantly enhanced growth rate in comparison to the other cell populations (Figure 5B, left panel), which was accompanied by deregulated ERK signaling, as demonstrated by increased levels of phosphorylated ERK, and an up-regulation of proliferative proteins such as cyclin D1 and cyclin A (Figure 5B, right panel). Furthermore, upon injection into the flanks of nude mice, only the RB-deficient p53DD, recurred cells were able to initiate tumor formation over the indicated time course (Figure 5C, top panel). Neither inactivation of p53 function alone, nor combined inactivation of RB and p53 resulted in tumor development. Similarly, the RB-proficient p53DD, recurred cells were incapable of forming tumors (Figure 5C, top panel). In contrast, injection of the RB-deficient p53DD, recurred cells resulted in the formation of tumors displaying significant cytological abnormalities. These tissues exhibited distinct nuclear pleomorphisms, nuclear inclusions, and high mitotic indexes, indicative of an undifferentiated sarcoma (Figure 5C, bottom panel).

Taken together, these findings indicate that RB is a critical suppressor of cellular transformation and tumorigenesis following therapy-induced damage, independent of p53 status. Furthermore, RB loss in the presence of therapy-induced damage can result in dramatic evolution of cells that can have significant impact on tumorigenesis not observed through loss of tumor suppressors alone.

### RB-Deficient Recurred Cells Display Enhanced Resistance to Discrete Therapeutic Challenges

**Figure 6:**
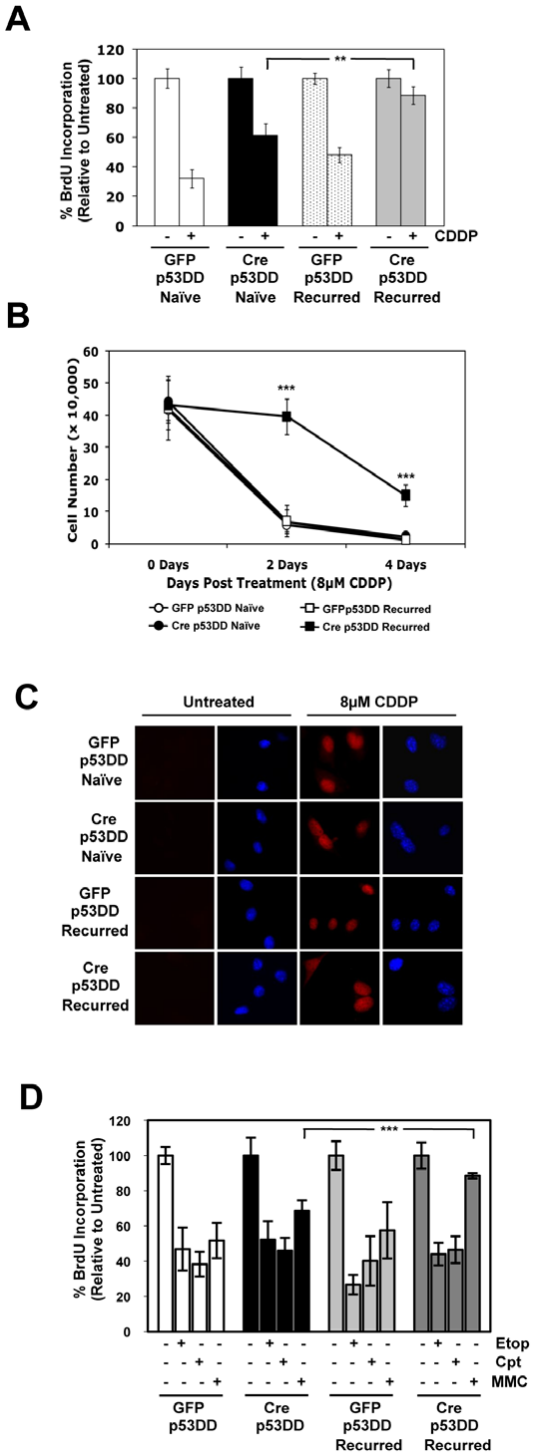
RB-deficiency promotes discrete therapeutic resistance after recovery from therapy-induced damage. (A) Cells were treated with 8 uM CDDP for 24 h, pulsed with BrdU for 1 h prior to harvesting, and processed as described in Figure 1C. Average percent BrdU incorporation relative to untreated controls is shown. **p = 0.0024 (B) Cells were treated with 8 uM CDDP for 24 h, allowed to recover in fresh media for 4 days, and viable cells were counted at indicated time points. ***p<0.0001 (C) Cells were treated with 8 uM CDDP for 24 h and stained for CDDP-adduct formation. Red, CDDP adducts; Blue, DNA stained with DAPI. (D) Cells were treated with etoposide (Etop, 1 uM), camptothecin (Cpt, 5 uM), or mitomycin C (MMC, 2 uM) for 24 h, pulsed with BrdU 1 h prior to harvesting, and processed as described in Figure 1C. Average percent BrdU incorporation relative to untreated controls is shown. ***p<0.0001.

Many cancer models have been shown to develop resistance to therapy [45]. As the loss of RB was observed to promote tumorigenesis after recovery from therapy-induced damage, the response of this cell population to subsequent treatment was evaluated. Both naïve and recurred, RB-proficient p53DD cells exhibited a significant decrease in replication upon treatment with CDDP (Figure 6A). Similar to previous results, naïve, RB-deficient cells were able to significantly bypass the DNA damage checkpoint compared to RB-proficient cells, though still displaying some sensitivity. Interestingly, RB-deficient p53DD cells that have recovered from an initial treatment of CDDP were more resistant to a second treatment, as compared to all other cell lines (Figure 6A), and the ability of the RB-deficient p53DD, recurred cell population to continue proliferating post-CDDP treatment was enhanced (Figure 6B). One potential reason for the differences in sensitivity to CDDP is related to an alteration in cellular uptake of the DNA damaging agent. As such, CDDP-adduct formation was examined within the cells by immunofluorescence. There was no significant difference in CDDP-adduct formation in any of the cell lines tested (Figure 6C). Thus, the differences in sensitivity to CDDP treatment observed between the cell lines are not due to variations in CDDP damage, but instead must be attributed to a difference in cellular response to the damage. To determine whether these findings represented a multi-drug resistance phenotype or specific resistance to CDDP, the cell populations were challenged with additional therapeutic agents. Interestingly, all cell populations displayed sensitivity to etoposide or camptothecin treatments (Figure 6D). However, RB-deficient p53DD, recurred cells displayed a resistance to mitomycin C treatment that was comparable to the CDDP resistance phenotype and not observed in any of the other cell lines (Figure 6D). Combined, these data indicate that loss of RB facilitates not only the transformed phenotype, but resistance to subsequent challenge with discrete therapeutic agents.

## Discussion

Cancer is one of many diseases that arise from multiple genetic and epigenetic alterations within a cell [2], [46], [47], [48]. Here, we dissected how two commonly inactivated tumor suppressors function in the response to therapy-induced damage and examined the consequence of such challenge in terms of oncogenic behavior and therapeutic resistance.

At a molecular level, the RB and p53 pathways intersect at multiple points that have implications for the behavior of tumors harboring distinct combinations of tumor suppressor pathway aberrations [18], [20]. Critically, these tumor suppressor pathways are key modulators of stress signals and represent an important barrier to tumorigenesis. Loss of RB and p53 has lead to enhanced tumor susceptibility in various cell types and mouse models in response to DNA damaging agents, demonstrating the importance of RB and p53 in mediating cellular response to exogenous stress [49], [50], [51]. Additionally, studies examining oncogene activation have indicated that certain types of endogenous stresses are capable of inducing DNA damage and subsequent p53 inactivation [52]. Loss if p53 function in this setting leads to genomic instability, which promotes cancer development. Interestingly, inactivation of RB itself also has been shown to elicit a damage response [53]. Furthermore, there are numerous additional endogenous stress signals, including metabolic, oxidative and mitotic stresses, which are characteristic of cancers and thought to be tolerated by cancer cells through inactivation of tumor suppressor pathways [54]. Thus, tumor suppressor pathways, such as the RB and p53 pathways, are critical for mediating cellular response to both exogenous and endogenous stress signals, and inactivation of these pathways paves the way for cancer development.

As shown here, deletion of RB and targeting of p53 with a dominant-negative strategy yielded a similar effect on the expression of genes that are regulated by the E2F family of transcription factors. This result is consistent with prior studies that show similar gene expression programs are regulated by these pathways in microarray analyses [14], [55]. In further keeping with these results, microarray analyses carried out in cells harboring the dominant negative p53 allele indicated little effect of RB deletion on gene expression programs in the absence of extrinsic stress. While overall these findings suggest that RB and p53 function in an overlapping pathway, there was a striking dependency on RB for the cell cycle inhibitory response to cisplatin. This role for RB was evident both in the context of acute and chronic exposures, a finding that is highly reflective of studies performed in human tumor models that suggest RB status is dominant to p53 status in determining cell cycle inhibition upon exposure to genotoxic therapeutic agents [25], [26]. Importantly, in the setting of chronic, low dose cisplatin exposure, there was a dramatic reduction in E2F-regulated targets, an effect that was singularly dependent on the presence of RB. In such conditions, RB-proficient cells establish a seemingly permanent growth arrest that is indicative of cellular senescence. By employing targeted deletion of RB in these populations, it was demonstrated that loss of RB is not only necessary for the initiation of this state, but also for its maintenance. Combined, these findings indicate that even in the presence of compromised p53 function, RB plays important roles in mediating specific cellular and molecular responses to DNA damage.

The composite loss of RB and p53 function is observed in multiple settings associated with cancer. For example, DNA tumor viruses have evolved key mechanisms that abrogate both tumor suppressor pathways in the same infected cell [56]. Here we investigated the dual influence of dysregulation of these key tumor suppressors in the context of oncogenic transformation. Interestingly, although p53 plays a dominant role in proliferation, the loss of RB was able to further accelerate the proliferation of such cell populations in cell culture. In spite of this growth advantage, dual RB/p53-deficient cells are not spontaneously transformed. For example, there is minimal tumor growth from these cells in nude mouse models. Thus, the transformed phenotype is a result of additional lesions beyond loss of these tumor suppressors alone. As shown here, cisplatin-mediated DNA damage can represent a key cooperating factor leading to oncogenic transformation.

Prior studies have demonstrated that RB loss can alter DNA ploidy in the presence of DNA damage, and tetraploid p53-deficient cells can readily progress to an oncogenic phenotype [57], [58], [59], [60]. Therefore, it was surprising in our model that minimal alterations in ploidy were observed, and there was no clear evidence of gross chromosomal instability. However, microarray analyses demonstrated significant changes in gene expression, specifically in the RB-deficient cell population that had recurred following CDDP treatment. Interestingly, these genes are neither directly regulated by p53 or RB/E2F, nor are they direct targets of acute DNA damage. Rather, a number of genes in this complex program corresponded to previously published “cooperation response genes” involved in a cooperative transformation process involving p53 [44]. Furthermore, there was dramatic down-regulation of a number of genes previously shown to be involved in suppressing tumorigenesis in various models [39], [40], [41], [42], [43]. This down-regulation was the result of alterations in the epigenetic programming of the cells, indicating that cooperative inactivation of the RB and p53 pathways can lead to modifications in epigenetic signaling. Taken together, these data suggest that the composite inactivation of RB and p53 has a profound impact on the response to genotoxic insult, enabling a genetic rewiring that is indicative of cellular transformation. In keeping with this concept, specifically RB-deficient, recurred cells exhibited a transformed phenotype and were tumorigenic in immune compromised mice.

Prior studies have implicated RB as playing a complex role in the response to chemotherapeutic agents [8], [25], [26], [27]. Upon exposure to cytotoxic agents, RB loss is associated with a predilection toward increased apoptotic death at a cellular level and enhanced sensitivity in specific xenograft models [25], [26], [27], [35]. In fact, even in the presence of compromised p53 function, an initial sensitivity to the cytotoxic effects of cisplatin in cells lacking RB was observed. However, under the low/clinically relevant doses utilized herein, viable cells uniformly escaped this treatment. We believe that this is significant, as recurrence following treatment with conventional cytotoxic chemotherapies is a well-established clinical problem. A striking characteristic of RB-deficient cells in response to CDDP is their failure to undergo a senescent-like cell cycle arrest. This finding has implications for therapeutic response, wherein the establishment of senescence has been associated with improved response to therapeutic approaches [61], and suggests that somatic loss of RB in tumors could contribute to ultimate tumorigenic outgrowth of the cell population. Importantly, it has been observed that Rb gene dosage has a significant impact on susceptibility to secondary malignancies induced by DNA damage (i.e. ionizing radiation) [30]. Thus, this pathway could also be relevant in the context of therapy-induced malignancies. Tumors that recur post-therapy are often exceedingly aggressive and resistant to subsequent therapeutic interventions. As shown here, the oncogenic behavior of cells lacking RB was particularly aggressive and exhibited resistance to subsequent rounds of cisplatin and mitomycin C treatment, but not treatment with etoposide or camptothecin. Thus, RB status could be an important determinant in directing treatment regimens for both primary and recurred malignancies.

Combined, these findings indicate that RB-deficient tumors are prone to significant evolution during therapeutic challenge, and reveal an additional mechanism by which RB loss promotes disease progression. Further studies investigating the impact of distinct environmental and therapeutic stresses on tumor suppressor pathways are beginning to reveal key facets of both tumor etiology and treatment response. Such analyses hold the promise of understanding both mechanisms to limit cancer progression and to more effectively intercede in clinical treatment.

## Supporting Information

Table S1 Supporting Information Table S1
(2.34 MB DOC)
