# Supplementary material for: Differential Impact of Tumor Suppressor Pathways on DNA Damage Response and Therapy-Induced Transformation in a Mouse Primary Cell Model
Source: PLoS One. 2010 Jan 1;5(1):e8558. doi: 10.1371/journal.pone.0008558 (PMC2796719; doi:10.1371/journal.pone.0008558)
Supplement: Table S1 — Supporting Information Table S1 (2.34 MB DOC) [file pone.0008558.s001.doc]

**Table S1**

Probe Set: Gene Symbol: Fold-change:

| 1452231_x_at | Sfrp1 | 0.006028995 |
| --- | --- | --- |
| 1434592_at | Rpl39l | 0.007041743 |
| 1456878_at | Grem2 | 0.008866234 |
| 1424850_at | Lum | 0.00906939 |
| 1455101_at | Chl1 | 0.009516095 |
| 1436566_at | Sfrp2 | 0.01055662 |
| 1417307_at | AW551984 | 0.011632877 |
| 1453313_at | Gpm6b | 0.011814042 |
| 1460600_at | Scara3 | 0.013161633 |
| 1454032_at | Sfrp1 | 0.0138375 |
| 1449152_at | Gm106 | 0.014516588 |
| 1456378_s_at | Clca1 | 0.015606343 |
| 1426852_x_at | Cpxm1 | 0.017958365 |
| 1436955_at | Sfrp1 | 0.020285387 |
| 1416200_at | Gpm6b | 0.020566814 |
| 1417312_at | Clca1 | 0.020618606 |
| 1435190_at | Slc14a1 | 0.021146528 |
| 1434151_at | Cyp2f2 | 0.022010557 |
| 1450117_at | Cd55 | 0.025007347 |
| 1426971_at | Atp8b1 | 0.025547257 |
| 1417552_at | Cd55 | 0.028724246 |
| 1417293_at | Egfl6 | 0.02888135 |
| 1417900_a_at | 2810022L02Rik | 0.02961172 |
| 1426318_at | Sfrp1 | 0.029794028 |
| 1417951_at | Gm106 | 0.029869635 |
| 1458394_at | Clu | 0.030072663 |
| 1418469_at | Chl1 | 0.030502217 |
| 1438910_a_at | Mmp3 | 0.03221355 |
| 1425914_a_at | Bnc1 | 0.032682307 |
| 1426013_s_at | Nnat | 0.032934856 |
| 1434497_at | LOC100044883 | 0.033913687 |
| 1420842_at | LOC100044883 | 0.03503284 |
| 1450976_at | 8030474K03Rik | 0.036203057 |
| 1418517_at | Chi3l1 | 0.038023323 |
| 1449878_a_at | Gdf10 | 0.038204897 |
| 1437959_at | --- | 0.03911012 |
| 1436990_s_at | 1500015O10Rik | 0.040004514 |
| 1450959_at | Tfpi2 | 0.041188076 |
| 1450454_at | Ripk3 | 0.04141659 |
| 1416221_at | Stox2 | 0.041571017 |
| 1448669_at | Isl1 | 0.042145047 |
| 1433712_at | Tshz2 | 0.04405989 |
| 1456765_at | Irx2 | 0.04668518 |
| 1455135_at | 9930013L23Rik | 0.049807597 |
| 1458916_at | Dmrt2 | 0.05316843 |
| 1431469_a_at | Ch25h | 0.053724952 |
| 1452719_at | Clu | 0.054029085 |
| 1428415_at | Ctsk | 0.054600302 |
| 1418135_at | E430002G05Rik | 0.055345174 |
| 1460474_at | Rgs2 | 0.055476706 |
| 1434171_at | Dio2 | 0.056735914 |
| 1422912_at | Kcnj15 | 0.05679053 |
| 1424783_a_at | Ptges | 0.058700014 |
| 1437162_at | Cd55 | 0.059033502 |
| 1417164_at | Igfbp3 | 0.06127631 |
| 1421034_a_at | Csf3 | 0.06158098 |
| 1438251_x_at | Il1rn | 0.061662227 |
| 1451567_a_at | Ptges | 0.062399693 |
| 1447670_at | Cdo1 | 0.06298446 |
| 1418185_at | Clu | 0.06378631 |
| 1429779_at | Mmp23 | 0.06662176 |
| 1452903_at | Cthrc1 | 0.06663064 |
| 1418052_at | Wnt5a | 0.067110814 |
| 1425964_x_at | AI747699 | 0.06732481 |
| 1435730_at | Mmp23 | 0.06775739 |
| 1452985_at | Rbp4 | 0.067902856 |
| 1454824_s_at | Gas6 | 0.07067 |
| 1451716_at | Auts2 | 0.07311508 |
| 1447704_s_at | Clu | 0.07342151 |
| 1454953_at | 2210016F16Rik | 0.073937505 |
| 1458245_at | Niban | 0.07761236 |
| 1416594_at | Hspa1a | 0.07848573 |
| 1438504_x_at | Niban | 0.08034805 |
| 1458947_at | Dpt | 0.08118816 |
| 1436388_a_at | D930003E18Rik | 0.08240514 |
| 1452365_at | Igfbp3 | 0.0832518 |
| 1417143_at | C1qtnf7 | 0.08345123 |
| 1423818_a_at | 2810405K02Rik | 0.08630771 |
| 1420590_at | Inhbb | 0.08923436 |
| 1428629_at | Rgs2 | 0.090078585 |
| 1433101_at | AI427515 | 0.091113746 |
| 1448293_at | Sorbs2 | 0.09166301 |
| 1435888_at | Svep1 | 0.09219642 |
| 1416287_at | Kctd15 | 0.09240399 |
| 1453556_x_at | Pde7b | 0.092605844 |
| 1448640_at | Vldlr | 0.09294602 |
| 1440687_at | Dapk1 | 0.092964925 |
| 1440662_at | Gda | 0.093110874 |
| 1427191_at | Cxcl16 | 0.09335845 |
| 1443007_at | Clca1 | 0.09359273 |
| 1442067_at | Cyp7b1 | 0.09488036 |
| 1426869_at | Gda | 0.097904064 |
| 1434364_at | Slc14a1 | 0.10068116 |
| 1416429_a_at | Apoe | 0.10238386 |
| 1435934_at | Vnn1 | 0.103185214 |
| 1459211_at | Wnt5a | 0.10367962 |
| 1423140_at | --- | 0.10553554 |
| 1451749_at | Ddx4 | 0.10576544 |
| 1428698_at | Abcc3 | 0.10680783 |
| 1420703_at | Ptger2 | 0.10778115 |
| 1419248_at | Tac1 | 0.10804548 |
| 1452404_at | Cyp7b1 | 0.108532354 |
| 1449082_at | Vldlr | 0.10868043 |
| 1423428_at | Isyna1 | 0.108717844 |
| 1442914_at | Penk1 | 0.10962755 |
| 1434380_at | Zfp105 | 0.10993062 |
| 1418547_at | Neto2 | 0.11115975 |
| 1455213_at | 1500009L16Rik | 0.11207874 |
| 1456174_x_at | Col24a1 | 0.118274406 |
| 1449006_at | C1qtnf7 | 0.119158134 |
| 1448136_at | --- | 0.11944864 |
| 1444289_at | 1110067I12Rik | 0.12019109 |
| 1433930_at | Cbr3 | 0.12166966 |
| 1444715_at | Prelp | 0.12267927 |
| 1436626_at | Aldh1l1 | 0.1253303 |
| 1457024_x_at | Rpgr | 0.12585591 |
| 1420127_s_at | Apod | 0.12626071 |
| 1445860_at | Mapk13 | 0.12646016 |
| 1444486_at | Il13ra2 | 0.12748 |
| 1457935_at | AI646023 | 0.12945734 |
| 1448359_a_at | --- | 0.12987356 |
| 1419666_x_at | Serping1 | 0.13144699 |
| 1451566_at | Mpped2 | 0.13260493 |
| 1421349_x_at | 1500015O10Rik | 0.13302721 |
| 1448395_at | Dnaja4 | 0.13349618 |
| 1435745_at | Rgs2 | 0.13372728 |
| 1435258_at | Slc38a4 | 0.1350096 |
| 1450769_s_at | C1qtnf2 | 0.13566828 |
| 1416647_at | Cadm2 | 0.13575977 |
| 1416432_at | Rgs4 | 0.13605143 |
| 1434030_at | Auts2 | 0.13608041 |
| 1428025_s_at | Steap4 | 0.13683772 |
| 1448968_at | a | 0.13779175 |
| 1427982_s_at | Steap4 | 0.13832714 |
| 1429582_at | Peli2 | 0.13836025 |
| 1417071_s_at | Csgalnact1 | 0.14220467 |
| 1436207_at | Lgi2 | 0.14234355 |
| 1422818_at | Iigp1 | 0.1424905 |
| 1418946_at | Larp6 | 0.14669898 |
| 1460670_at | Iigp1 | 0.14686096 |
| 1426481_at | Adam33 | 0.1469672 |
| 1460116_s_at | Ccl8 | 0.14790772 |
| 1448823_at | 1700112E06Rik | 0.14800742 |
| 1436520_at | Cpxm2 | 0.14847472 |
| 1428669_at | Prkar1b | 0.14862563 |
| 1455447_at | Fbln7 | 0.14863291 |
| 1416628_at | Mmp13 | 0.1492473 |
| 1459897_a_at | Cxadr | 0.14956823 |
| 1452646_at | Thbs2 | 0.14997688 |
| 1448919_at | Gch1 | 0.15029843 |
| 1444232_at | LOC100040525 | 0.1508987 |
| 1451019_at | Crabp2 | 0.15267467 |
| 1434902_at | Zfp709 | 0.15309466 |
| 1456212_x_at | Serpina3g | 0.153634 |
| 1416700_at | Isl1 | 0.15429331 |
| 1430353_at | Gfpt2 | 0.15475374 |
| 1460208_at | Rtn4rl2 | 0.15856794 |
| 1418359_at | Il16 | 0.15962133 |
| 1429401_at | 0610006I08Rik | 0.16027941 |
| 1455631_at | Thbs2 | 0.16106331 |
| 1436676_at | Mark1 | 0.16123252 |
| 1431931_a_at | Flywch2 | 0.16150975 |
| 1424180_a_at | Rb1 | 0.16308734 |
| 1416985_at | Mark1 | 0.1631375 |
| 1425016_at | --- | 0.16385144 |
| 1426893_at | Ednrb | 0.16395783 |
| 1450243_a_at | Cxcl14 | 0.16406918 |
| 1424892_at | Dio2 | 0.16481626 |
| 1418537_at | Adam33 | 0.16536236 |
| 1458436_at | 1110018M03Rik | 0.1667639 |
| 1452359_at | Smoc2 | 0.16742608 |
| 1457184_at | Csgalnact1 | 0.1680985 |
| 1456011_x_at | Gpnmb | 0.16841465 |
| 1443249_at | Iqgap2 | 0.16909985 |
| 1428321_at | Il1rn | 0.16946216 |
| 1437197_at | C920005C14Rik | 0.16964965 |
| 1449531_at | Rnf130 | 0.16989593 |
| 1437087_at | Vcam1 | 0.1703349 |
| 1427293_a_at | Thbs2 | 0.17047107 |
| 1451604_a_at | Tnc | 0.17188455 |
| 1433939_at | Angptl4 | 0.17400473 |
| 1416625_at | Asb4 | 0.1750267 |
| 1417777_at | Rasl11a | 0.17568706 |
| 1425829_a_at | Slc2a3 | 0.17614351 |
| 1453152_at | Fbn1 | 0.17615606 |
| 1460252_s_at | Rarres1 | 0.1764733 |
| 1436320_at | --- | 0.17649798 |
| 1440668_at | Ptgfr | 0.1765896 |
| 1433452_at | Vcam1 | 0.17804381 |
| 1425591_a_at | Clip4 | 0.1805427 |
| 1433993_at | Aff3 | 0.18097374 |
| 1457701_at | 4632428N05Rik | 0.1818931 |
| 1438317_a_at | B4galnt1 | 0.1842012 |
| 1432946_at | AW548124 | 0.18440592 |
| 1417980_a_at | Tnfrsf11b | 0.18448523 |
| 1426534_a_at | Kcne4 | 0.18710826 |
| 1455038_at | Mfap5 | 0.18931516 |
| 1456609_at | Slc38a1 | 0.18942457 |
| 1434520_at | Rcan2 | 0.18949482 |
| 1423854_a_at | Slc2a3 | 0.19142191 |
| 1420632_a_at | Il1rn | 0.1917658 |
| 1452937_s_at | Il1rl1 | 0.19257122 |
| 1434510_at | Trim44 | 0.19400291 |
| 1458121_at | Ptges | 0.19472617 |
| 1419208_at | OTTMUSG00000010657 | 0.19500518 |
| 1447288_at | Ahr | 0.19621104 |
| 1428651_at | Syce2 | 0.19645166 |
| 1437399_at | Fst | 0.19652171 |
| 1460187_at | Dnaja4 | 0.19675866 |
| 1446934_at | Wnt4 | 0.19716592 |
| 1452257_at | Ptgfr | 0.19749388 |
| 1419667_at | Dact1 | 0.19765413 |
| 1417174_at | Pparg | 0.1978658 |
| 1460586_at | Cbr1 | 0.19797838 |
| 1443589_at | Ahrr | 0.1985814 |
| 1421977_at | Sulf2 | 0.19883355 |
| 1418181_at | Rnf113a2 | 0.19894558 |
| 1435749_at | Vldlr | 0.19983463 |
| 1442129_at | Vit | 0.20061278 |
| 1441547_at | Lama4 | 0.20253582 |
| 1421163_a_at | --- | 0.20315206 |
| 1456523_at | Mtm1 | 0.20343992 |
| 1449318_at | Trim44 | 0.20378375 |
| 1451131_at | EG628412 | 0.20391822 |
| 1426530_a_at | Trim44 | 0.20446154 |
| 1439616_at | Fmod | 0.20494099 |
| 1450207_at | Acsl6 | 0.20510316 |
| 1418655_at | Peli2 | 0.2057536 |
| 1448021_at | Klhl22 | 0.20613037 |
| 1458648_at | Hivep3 | 0.2062717 |
| 1452388_at | Arnt2 | 0.20745851 |
| 1426856_at | G530011O06Rik | 0.20872164 |
| 1436678_at | Tlr2 | 0.20942675 |
| 1430887_s_at | Vcam1 | 0.21142167 |
| 1451119_a_at | Ror2 | 0.212007 |
| 1452340_at | Zfp518 | 0.21284658 |
| 1443714_at | Lgi2 | 0.21321023 |
| 1436636_at | Fut11 | 0.2141786 |
| 1422645_at | Rnf157 | 0.21488193 |
| 1416978_at | Dpep1 | 0.21555844 |
| 1422479_at | Prelp | 0.21613885 |
| 1416371_at | D430019H16Rik | 0.21668069 |
| 1437454_a_at | Foxf1a | 0.21746643 |
| 1438732_at | BC022623 | 0.2175834 |
| 1460194_at | Cap1 | 0.21841292 |
| 1434815_a_at | Gpm6b | 0.21941787 |
| 1459878_a_at | Gch1 | 0.22152214 |
| 1423091_a_at | Nbl1 | 0.22157663 |
| 1449388_at | Vcam1 | 0.22386001 |
| 1437227_at | Ptx3 | 0.2240098 |
| 1419665_a_at | Pctp | 0.2258153 |
| 1452068_at | 5430433G21Rik | 0.22655301 |
| 1449089_at | --- | 0.22696634 |
| 1428767_at | Stox2 | 0.22764058 |
| 1418677_at | Il1rl1 | 0.22770698 |
| 1452968_at | U90926 | 0.22775112 |
| 1417230_at | Ptprm | 0.22828092 |
| 1423327_at | --- | 0.22912617 |
| 1436405_at | 1110062M06Rik | 0.23231524 |
| 1437829_s_at | Sfrp1 | 0.23278105 |
| 1435162_at | AW551984 | 0.23341817 |
| 1451290_at | AI256396 | 0.23368321 |
| 1416473_a_at | Mpped2 | 0.23410997 |
| 1451362_at | Lrrc8d | 0.23411874 |
| 1458096_at | Ephb2 | 0.23488286 |
| 1447624_s_at | Fap | 0.23495004 |
| 1449024_a_at | Fmod | 0.23512128 |
| 1450425_a_at | Bmp4 | 0.23595698 |
| 1453386_at | --- | 0.23596348 |
| 1415977_at | --- | 0.2374258 |
| 1419606_a_at | 6430511F03 | 0.23747149 |
| 1453173_at | A230050P20Rik | 0.23758367 |
| 1422540_at | Slc38a4 | 0.23843002 |
| 1438635_x_at | Mfap5 | 0.23970523 |
| 1434384_at | Ror1 | 0.2398226 |
| 1418446_at | Cpeb1 | 0.24103597 |
| 1454781_x_at | Hivep3 | 0.2425371 |
| 1423311_s_at | Fmod | 0.24345067 |
| 1438620_x_at | Tnc | 0.24345951 |
| 1428114_at | Enpp2 | 0.24439673 |
| 1435088_at | Plagl1 | 0.24450025 |
| 1417753_at | D14Ertd668e | 0.24598023 |
| 1454295_at | Glis3 | 0.24648078 |
| 1454764_s_at | Nfia | 0.24659847 |
| 1458963_at | Npas2 | 0.24728252 |
| 1440147_at | Ifi203 | 0.24767768 |
| 1450782_at | Ppap2b | 0.24829035 |
| 1434550_at | Btbd14a | 0.25027454 |
| 1430904_at | Tgm1 | 0.2507601 |
| 1422943_a_at | Wnk3 | 0.25178295 |
| 1453851_a_at | 3830406C13Rik | 0.2536575 |
| 1423160_at | Mtus1 | 0.25556958 |
| 1448799_s_at | Fmod | 0.25561687 |
| 1444108_at | Ppap2b | 0.2556181 |
| 1438982_s_at | Il33 | 0.2565027 |
| 1417399_at | Plekhb2 | 0.25677493 |
| 1453004_at | Tmem181 | 0.25721252 |
| 1452738_at | Ptgfr | 0.25855562 |
| 1443157_at | --- | 0.25936902 |
| 1431475_a_at | Pde1a | 0.26096964 |
| 1417730_at | 5830408B19Rik | 0.26098663 |
| 1429134_at | Mark1 | 0.2610424 |
| 1428346_at | LOC672215 | 0.2621368 |
| 1422980_a_at | Crlf1 | 0.2624336 |
| 1434291_a_at | Rora | 0.26278865 |
| 1428647_at | LOC100047963 | 0.26293412 |
| 1434379_at | 1200016E24Rik | 0.26296994 |
| 1454708_at | 2210016F16Rik | 0.26328114 |
| 1433994_at | Nfia | 0.26336232 |
| 1452848_at | Wisp2 | 0.26352328 |
| 1423266_at | Sema3f | 0.2636214 |
| 1457673_at | Cadm2 | 0.2636906 |
| 1417391_a_at | Cyp4v3 | 0.26410443 |
| 1457719_at | --- | 0.26536068 |
| 1432129_a_at | Rerg | 0.26637763 |
| 1433919_at | Tm7sf3 | 0.26678687 |
| 1431300_at | Ppp1r3b | 0.2673479 |
| 1459434_at | Cacna1g | 0.26774758 |
| 1460500_at | Fut11 | 0.26776642 |
| 1450826_a_at | Mtus1 | 0.26780066 |
| 1459552_at | Bscl2 | 0.26836348 |
| 1440007_at | Sgcb | 0.26876104 |
| 1449062_at | Pfkfb3 | 0.27003074 |
| 1423889_at | Lifr | 0.2701312 |
| 1450586_at | Spin2 | 0.2701374 |
| 1460634_at | --- | 0.27064377 |
| 1416543_at | Abat | 0.27076808 |
| 1428660_s_at | Birc3 | 0.27132553 |
| 1440292_at | --- | 0.2714421 |
| 1439422_a_at | Hoxc10 | 0.27150348 |
| 1431173_at | Slc10a6 | 0.27197242 |
| 1452418_at | LOC100046643 | 0.2719763 |
| 1424456_at | Hist1h2bb | 0.27198082 |
| 1435251_at | 9530066K23Rik | 0.27200022 |
| 1454983_at | Ppp1r3c | 0.27236563 |
| 1436332_at | Clip4 | 0.27245763 |
| 1421594_a_at | Csf2ra | 0.27327484 |
| 1417147_at | Bdh1 | 0.2751527 |
| 1425631_at | A030009H04Rik | 0.27537802 |
| 1436574_at | Rcan2 | 0.27584547 |
| 1437403_at | Slc16a10 | 0.27599174 |
| 1418486_at | Ptp4a3 | 0.276659 |
| 1417937_at | --- | 0.27673277 |
| 1422571_at | 2010305C02Rik | 0.2776154 |
| 1437771_at | Dpep1 | 0.2777204 |
| 1431035_at | Tnfsf13b | 0.2786456 |
| 1438868_at | Mafb | 0.279701 |
| 1455768_at | Pitx2 | 0.28033188 |
| 1426851_a_at | Rab40b | 0.2809426 |
| 1432260_at | Lifr | 0.28154656 |
| 1456981_at | Il4ra | 0.2821583 |
| 1454984_at | Btbd14a | 0.28233474 |
| 1433434_at | Arl6ip1 | 0.28338045 |
| 1445095_at | Slc38a1 | 0.2834723 |
| 1450784_at | AL024213 | 0.28432435 |
| 1421097_at | Serpinb1b | 0.2844649 |
| 1428384_at | --- | 0.2853069 |
| 1430045_at | Aldoc | 0.28535846 |
| 1440966_at | Mtm1 | 0.28609514 |
| 1460170_at | Tnfrsf21 | 0.28655437 |
| 1454904_at | 1700112E06Rik | 0.28674695 |
| 1428136_at | Endog | 0.28699955 |
| 1425328_at | Cxadr | 0.28755578 |
| 1455646_at | --- | 0.28791657 |
| 1435486_at | Mrgprf | 0.28798723 |
| 1429977_at | --- | 0.28816164 |
| 1436799_at | AU042950 | 0.2883932 |
| 1459606_at | Mylk | 0.28867933 |
| 1430012_at | Tiam1 | 0.28918225 |
| 1428988_at | Mark1 | 0.28929397 |
| 1437268_at | Rgs4 | 0.28965977 |
| 1449118_at | C1qdc2 | 0.2897209 |
| 1427407_s_at | Ly6a | 0.28977373 |
| 1427629_at | Mmp19 | 0.290366 |
| 1446498_at | Klhl23 | 0.2915549 |
| 1452379_at | Camk2n1 | 0.29260182 |
| 1449033_at | Arl6ip1 | 0.29367825 |
| 1455087_at | Tslp | 0.29410708 |
| 1448960_at | Mfap2 | 0.2952124 |
| 1457429_s_at | Micalcl | 0.29525325 |
| 1416576_at | Ankrd29 | 0.29532796 |
| 1436137_at | Ctla2a | 0.29537705 |
| 1428401_at | Cend1 | 0.2957463 |
| 1436448_a_at | Phactr2 | 0.2960297 |
| 1457426_at | C630028N24Rik | 0.2964915 |
| 1436309_at | EG628412 | 0.29709953 |
| 1435468_at | Rora | 0.29762265 |
| 1434758_at | Cyp2j6 | 0.29769552 |
| 1416017_at | Dapk1 | 0.298605 |
| 1420973_at | Oasl2 | 0.29948622 |
| 1421065_at | Ifi203 | 0.29968014 |
| 1451332_at | Gstm4 | 0.30031362 |
| 1419834_x_at | Il1r1 | 0.30081245 |
| 1424319_at | EG633640 | 0.3011581 |
| 1418589_a_at | 3830406C13Rik | 0.30358693 |
| 1434483_at | Glt8d4 | 0.3042552 |
| 1421348_a_at | Map3k8 | 0.30488113 |
| 1423753_at | Rab3il1 | 0.30497003 |
| 1435071_at | Tnfaip2 | 0.30524388 |
| 1418538_at | Ifi203 | 0.30551803 |
| 1450723_at | Rasl11b | 0.30589175 |
| 1420312_s_at | Cercam | 0.30604762 |
| 1460458_at | Ptprz1 | 0.3077778 |
| 1438954_x_at | Oplah | 0.30798793 |
| 1423062_at | LOC100047743 | 0.30851403 |
| 1438236_at | Klf15 | 0.30915532 |
| 1423413_at | LOC100044204 | 0.31091884 |
| 1423707_at | Ccl7 | 0.31183589 |
| 1452273_at | Hpse | 0.31189337 |
| 1455323_at | Mogat2 | 0.3123055 |
| 1418070_at | Neto2 | 0.3124469 |
| 1439789_at | B930041F14Rik | 0.31277484 |
| 1448471_a_at | Irx1 | 0.31280905 |
| 1433481_at | --- | 0.31300506 |
| 1446771_at | Cyp4v3 | 0.31379184 |
| 1441145_at | Cnrip1 | 0.31386563 |
| 1417359_at | Cxcl16 | 0.3144553 |
| 1417153_at | Ctla2a | 0.31474867 |
| 1429351_at | --- | 0.31490716 |
| 1456941_at | Ctsf | 0.31492877 |
| 1417282_at | Mark1 | 0.31500685 |
| 1433795_at | Tuba8 | 0.3158359 |
| 1442140_at | Prickle2 | 0.31607524 |
| 1460229_at | BC034090 | 0.31662717 |
| 1428187_at | Dcn | 0.31790873 |
| 1416286_at | LOC676870 | 0.31842655 |
| 1418592_at | LOC245350 | 0.3187777 |
| 1448985_at | Rspo3 | 0.3193164 |
| 1434414_at | Map3k1 | 0.31957227 |
| 1423296_at | DXErtd242e | 0.31980014 |
| 1453171_s_at | Synpo | 0.32125944 |
| 1426940_at | Fbn1 | 0.32134566 |
| 1450259_a_at | Sulf2 | 0.32217103 |
| 1451533_at | Ugcg | 0.32261163 |
| 1436193_at | Naprt1 | 0.32348534 |
| 1453071_s_at | Npl | 0.32383835 |
| 1426574_a_at | Rnf157 | 0.3256595 |
| 1417001_a_at | C1qdc2 | 0.3286557 |
| 1449630_s_at | Tuba8 | 0.329205 |
| 1426906_at | 2010004M13Rik | 0.32982397 |
| 1449449_at | --- | 0.3299574 |
| 1424835_at | Bmyc | 0.3304941 |
| 1434310_at | Galc | 0.33217666 |
| 1455854_a_at | Gstt1 | 0.33233887 |
| 1426915_at | Fstl3 | 0.33263102 |
| 1454858_x_at | Ptgir | 0.33296013 |
| 1417527_at | 9530096D07Rik | 0.3330767 |
| 1415897_a_at | Il13ra1 | 0.33362842 |
| 1419255_at | Ptprz1 | 0.33438745 |
| 1449602_at | Srpx2 | 0.33492103 |
| 1433885_at | Lrrc8d | 0.33509076 |
| 1417505_s_at | Hist3h2a | 0.3359569 |
| 1440355_at | Bace2 | 0.33615908 |
| 1460411_s_at | Mlf1 | 0.3366949 |
| 1447014_at | Nudt6 | 0.3369907 |
| 1433695_at | B230380D07Rik | 0.33715764 |
| 1460163_at | Angpt1 | 0.33720613 |
| 1418937_at | --- | 0.33732465 |
| 1417382_at | Prkar1b | 0.33776698 |
| 1425107_a_at | Rnf157 | 0.33915678 |
| 1460302_at | Fbn1 | 0.33992827 |
| 1418492_at | Npr2 | 0.34018368 |
| 1444408_at | Nsd1 | 0.34123293 |
| 1455161_at | Il4ra | 0.34261465 |
| 1432268_at | Mfsd9 | 0.34263784 |
| 1444045_at | Sgcb | 0.3426939 |
| 1448237_x_at | Slc16a3 | 0.3430346 |
| 1453058_at | Stag3 | 0.34386465 |
| 1454614_at | --- | 0.34401184 |
| 1451895_a_at | Btbd6 | 0.3442458 |
| 1447854_s_at | Rbmx | 0.34461996 |
| 1451043_at | Tm7sf3 | 0.34569618 |
| 1435691_at | Rgl1 | 0.34637386 |
| 1454965_at | LOC100046086 | 0.3465059 |
| 1416164_at | Tmem141 | 0.34763312 |
| 1452979_at | Med24 | 0.34827018 |
| 1424007_at | Psd3 | 0.348954 |
| 1455030_at | Gbp6 | 0.3493671 |
| 1454930_at | Aox1 | 0.35111922 |
| 1457231_at | --- | 0.35163984 |
| 1437458_x_at | Ssh3 | 0.351726 |
| 1441746_at | Mtus1 | 0.35187915 |
| 1448181_at | Tmc4 | 0.35214648 |
| 1433639_at | Auts2 | 0.35299835 |
| 1432415_at | 6030451C04Rik | 0.3531256 |
| 1428111_at | LOC100045019 | 0.35327706 |
| 1416537_at | Rnf157 | 0.3538151 |
| 1434962_x_at | Trp53inp1 | 0.35438448 |
| 1436543_at | Prkar1b | 0.35440812 |
| 1424544_at | C730049O14Rik | 0.35538396 |
| 1429111_at | Aldh3a1 | 0.3555035 |
| 1440133_x_at | Prkar1b | 0.3555366 |
| 1448901_at | Pcdhb19 | 0.3558441 |
| 1427270_a_at | Col12a1 | 0.35734057 |
| 1429335_at | Sgcb | 0.35750186 |
| 1423233_at | Dab2 | 0.3583502 |
| 1424988_at | Tspyl3 | 0.35960406 |
| 1434415_at | Boc | 0.36069024 |
| 1423672_at | Nov | 0.36093378 |
| 1420984_at | Il11ra1 | 0.36102185 |
| 1432282_a_at | Il3ra | 0.36148688 |
| 1448996_at | Ralgds | 0.36151597 |
| 1426261_s_at | Zfp704 | 0.36161944 |
| 1452872_at | LOC100041546 | 0.36170658 |
| 1425180_at | Sema3c | 0.3623652 |
| 1420653_at | 4-Sep | 0.3623935 |
| 1452145_at | Mitf | 0.3626545 |
| 1455040_s_at | Rab27b | 0.36313695 |
| 1416846_a_at | --- | 0.36497116 |
| 1427912_at | Rora | 0.36580458 |
| 1451798_at | Sgip1 | 0.36630562 |
| 1435705_at | --- | 0.3668176 |
| 1448477_at | Slc39a8 | 0.3670086 |
| 1433084_at | Nov | 0.36724937 |
| 1450644_at | Crispld2 | 0.3677409 |
| 1452008_at | Hspb6 | 0.36834362 |
| 1441643_at | 3-Sep | 0.3689684 |
| 1438602_s_at | Parp12 | 0.36946347 |
| 1437673_at | Zcchc3 | 0.3695393 |
| 1440651_at | --- | 0.36971146 |
| 1430127_a_at | Tnn | 0.37034705 |
| 1451244_a_at | Foxred2 | 0.37037414 |
| 1449641_at | --- | 0.37066892 |
| 1454628_at | Lifr | 0.3709788 |
| 1425527_at | Serf1 | 0.3713303 |
| 1425511_at | Phactr2 | 0.37337425 |
| 1424103_at | Heg1 | 0.37350845 |
| 1450881_s_at | Crispld2 | 0.37398592 |
| 1422803_at | Fbln1 | 0.3749921 |
| 1436101_at | 2310014G06Rik | 0.37657472 |
| 1417389_at | Il13ra1 | 0.3772564 |
| 1455401_at | AA409316 | 0.3787762 |
| 1416767_a_at | Nfia | 0.37902248 |
| 1424265_at | 2510009E07Rik | 0.3795745 |
| 1434051_s_at | Sgce | 0.37977192 |
| 1422822_at | Gli2 | 0.37989995 |
| 1424763_at | Adal | 0.3802391 |
| 1419532_at | Enpp2 | 0.38032266 |
| 1438038_at | Asb4 | 0.38200396 |
| 1437763_at | Slit3 | 0.3823943 |
| 1455099_at | Lysmd2 | 0.383588 |
| 1443926_at | Bach2 | 0.38360217 |
| 1418753_at | Endog | 0.38364586 |
| 1417384_at | OTTMUSG00000008561 | 0.38436222 |
| 1434334_at | Mab21l1 | 0.38456813 |
| 1433435_at | Pcdhb22 | 0.38506418 |
| 1417703_at | Dmd | 0.38554153 |
| 1423161_s_at | 2810047C21Rik | 0.38565964 |
| 1434939_at | Syne2 | 0.38684136 |
| 1427321_s_at | Ptplad2 | 0.38685 |
| 1452761_a_at | Dkk3 | 0.3870177 |
| 1460626_at | LOC632297 | 0.387406 |
| 1447928_at | 4930488E11Rik | 0.38840568 |
| 1418457_at | Tceal1 | 0.38863504 |
| 1427086_at | --- | 0.3887271 |
| 1416982_at | --- | 0.38907164 |
| 1454726_s_at | LOC100045055 | 0.3891005 |
| 1417281_a_at | C76566 | 0.38925636 |
| 1435357_at | Figf | 0.3899555 |
| 1442392_at | Eif4g3 | 0.3899902 |
| 1433979_at | Lcn2 | 0.3912332 |
| 1437324_x_at | Crispld2 | 0.3912978 |
| 1434280_at | Antxr1 | 0.3917432 |
| 1417130_s_at | Man1c1 | 0.39183894 |
| 1429682_at | Arhgef6 | 0.3924841 |
| 1439814_at | Slc16a2 | 0.39251783 |
| 1438498_at | Ktelc1 | 0.39262956 |
| 1448229_s_at | Timp3 | 0.39282444 |
| 1438055_at | Il17ra | 0.3928424 |
| 1448449_at | Rgl1 | 0.39300478 |
| 1455697_at | B230380D07Rik | 0.3932695 |
| 1448316_at | Osbpl6 | 0.39391267 |
| 1440498_at | Ube1l | 0.39400107 |
| 1423017_a_at | 6230427J02Rik | 0.39439467 |
| 1449488_at | Timp3 | 0.39501008 |
| 1417924_at | Kitl | 0.39591306 |
| 1448482_at | Slc16a10 | 0.3959912 |
| 1434786_at | Slc6a17 | 0.3961754 |
| 1449070_x_at | Slc1a6 | 0.39662832 |
| 1424475_at | --- | 0.39703533 |
| 1417795_at | Il17ra | 0.39788547 |
| 1424740_at | --- | 0.39820287 |
| 1434325_x_at | Slc22a23 | 0.3983652 |
| 1460341_at | Tspan11 | 0.39902136 |
| 1448925_at | Adamtsl3 | 0.40008977 |
| 1424420_at | Mmp2 | 0.40019104 |
| 1442169_at | Prkg1 | 0.4011403 |
| 1426818_at | Papss2 | 0.40127358 |
| 1422896_at | Bach2 | 0.40180573 |
| 1448619_at | A330021E22Rik | 0.40188366 |
| 1420796_at | Btbd14a | 0.40260258 |
| 1448842_at | BC031353 | 0.40398967 |
| 1416013_at | Ebf1 | 0.40413424 |
| 1442005_at | Tnfaip2 | 0.40420172 |
| 1458268_s_at | Cpeb3 | 0.4043164 |
| 1459660_at | Acvr1b | 0.4046944 |
| 1459348_at | Grb10 | 0.40524516 |
| 1436041_at | OTTMUSG00000010657 | 0.40524873 |
| 1446331_at | Fcgrt | 0.40526807 |
| 1451260_at | Ppp1r3d | 0.40636617 |
| 1453054_at | Ifi203 | 0.40704235 |
| 1447852_x_at | Plekha4 | 0.4073394 |
| 1438953_at | Calml4 | 0.4076633 |
| 1423298_at | Unc93b1 | 0.4079117 |
| 1438680_at | LOC634731 | 0.409201 |
| 1434034_at | Figf | 0.40991405 |
| 1453569_s_at | Lefty1 | 0.41006234 |
| 1438870_at | Figf | 0.41041616 |
| 1429692_s_at | Reck | 0.4106356 |
| 1454773_at | Cxcl12 | 0.41102517 |
| 1427038_at | Tln2 | 0.41130134 |
| 1455165_at | Abcc9 | 0.41156152 |
| 1451477_at | Zcchc3 | 0.41201428 |
| 1449012_s_at | Mmp2 | 0.41298702 |
| 1460065_at | Pbx1 | 0.4130919 |
| 1419097_a_at | 2610028L16Rik | 0.4133712 |
| 1429273_at | Add3 | 0.41382107 |
| 1454161_s_at | Timp3 | 0.41385368 |
| 1435948_at | Zhx1 | 0.41388512 |
| 1452412_at | 2810047C21Rik | 0.4145077 |
| 1419080_at | 6230416C02Rik | 0.41510323 |
| 1429418_at | Arl6ip1 | 0.41528296 |
| 1447929_at | Tshz2 | 0.41601083 |
| 1427934_at | Sipa1l1 | 0.41620237 |
| 1457881_at | Ccnd2 | 0.41633466 |
| 1437613_s_at | Parc | 0.41676328 |
| 1431382_a_at | A330021E22Rik | 0.41687033 |
| 1416354_at | Lsm14a | 0.41709343 |
| 1434830_at | 1110050K14Rik | 0.41759112 |
| 1424784_at | Pitx1 | 0.41789162 |
| 1443924_at | Aplp1 | 0.41810197 |
| 1420583_a_at | Spry2 | 0.4181552 |
| 1419163_s_at | --- | 0.4188884 |
| 1416811_s_at | Spon2 | 0.41993874 |
| 1436368_at | Kank1 | 0.4202192 |
| 1419089_at | Fgd4 | 0.42033777 |
| 1455090_at | --- | 0.42033988 |
| 1427242_at | Hspa1b | 0.42040148 |
| 1436590_at | Tspan11 | 0.42056558 |
| 1429799_at | Fhod3 | 0.4211998 |
| 1417256_at | Cxxc5 | 0.42126465 |
| 1457040_at | --- | 0.4218211 |
| 1423673_at | B230380D07Rik | 0.42215073 |
| 1456495_s_at | Npepl1 | 0.42233154 |
| 1415935_at | --- | 0.42293286 |
| 1435066_at | Il6st | 0.42358413 |
| 1426110_a_at | --- | 0.42364165 |
| 1418511_at | Eid2 | 0.42380747 |
| 1460684_at | Sned1 | 0.42429793 |
| 1427233_at | Slc16a2 | 0.4246984 |
| 1426363_x_at | 9030425E11Rik | 0.4251483 |
| 1433454_at | Il6st | 0.4252196 |
| 1434130_at | Fas | 0.42535642 |
| 1435460_at | 2310007H09Rik | 0.42612723 |
| 1439839_at | --- | 0.42613286 |
| 1452363_a_at | Dab2 | 0.4262834 |
| 1418074_at | Irx3 | 0.42664948 |
| 1450967_at | Pogk | 0.42680195 |
| 1435551_at | Gpr137b | 0.4275773 |
| 1435339_at | Zfp810 | 0.42758983 |
| 1421998_at | Cxxc5 | 0.4281337 |
| 1454849_x_at | Zfp575 | 0.4283986 |
| 1440169_x_at | LOC632297 | 0.4285908 |
| 1456335_at | Auh | 0.4287532 |
| 1429888_a_at | Cd276 | 0.42910466 |
| 1424072_at | LOC632297 | 0.4293429 |
| 1424094_at | Tspyl3 | 0.42952716 |
| 1453372_at | LOC676870 | 0.4296335 |
| 1439433_a_at | AU041133 | 0.4297159 |
| 1439066_at | LOC100047963 | 0.4306193 |
| 1438042_at | 1700048O20Rik | 0.4311697 |
| 1434580_at | Pcdh7 | 0.4315121 |
| 1455428_at | Gp1bb | 0.4316409 |
| 1448939_at | Il13ra1 | 0.43173835 |
| 1440041_at | Nkd1 | 0.43185136 |
| 1432432_a_at | Hspa1b | 0.43189904 |
| 1457344_at | Dcn | 0.43194416 |
| 1427313_at | Hspa1b | 0.43203118 |
| 1418454_at | --- | 0.4323747 |
| 1433529_at | Trp53inp2 | 0.43238184 |
| 1434861_at | Dnmt3l | 0.43246877 |
| 1431364_a_at | Saa3 | 0.43305224 |
| 1422804_at | Dhx58 | 0.43433324 |
| 1454675_at | Sema3c | 0.4347968 |
| 1435175_at | Fbln1 | 0.43496394 |
| 1447788_s_at | Agpat2 | 0.43534204 |
| 1421037_at | Timp3 | 0.4368142 |
| 1442595_at | Pbx1 | 0.4379127 |
| 1425784_a_at | Tm7sf3 | 0.43803632 |
| 1443363_at | Lipa | 0.4382777 |
| 1442116_at | Pde7b | 0.4384669 |
| 1422541_at | Dhcr24 | 0.4389591 |
| 1417568_at | Nope | 0.43896112 |
| 1424669_at | Acvrl1 | 0.44006965 |
| 1448243_at | Ptgs1 | 0.44007045 |
| 1449731_s_at | Ccnd2 | 0.44189596 |
| 1426383_at | Sesn3 | 0.4425799 |
| 1451970_at | AW548124 | 0.4429697 |
| 1425035_s_at | Samd5 | 0.4434425 |
| 1448558_a_at | C230093N12Rik | 0.44373327 |
| 1426913_at | B230317C12Rik | 0.44541696 |
| 1453540_at | Cd302 | 0.44567645 |
| 1449542_at | Olfm1 | 0.44628644 |
| 1448663_s_at | Shisa4 | 0.4463878 |
| 1453775_at | Cxcl12 | 0.4468646 |
| 1416749_at | Wtip | 0.44752443 |
| 1451430_at | Tor3a | 0.44790202 |
| 1459961_a_at | Kitl | 0.44807267 |
| 1437245_at | Lss | 0.44866684 |
| 1460248_at | Il1rap | 0.44883823 |
| 1423607_at | Hspb1 | 0.44887018 |
| 1439798_at | Rora | 0.44973266 |
| 1426315_a_at | Pdk2 | 0.45023984 |
| 1415943_at | LOC100044115 | 0.4505224 |
| 1428692_at | 5430435G22Rik | 0.45100132 |
| 1419684_at | Entpd5 | 0.45116174 |
| 1454748_at | Pros1 | 0.45130607 |
| 1419518_at | Prl2c2 | 0.45184967 |
| 1427485_at | Zbtb4 | 0.4527425 |
| 1417565_at | Dlg2 | 0.45319015 |
| 1428097_at | Il20ra | 0.45334357 |
| 1420123_at | Nsd1 | 0.45353276 |
| 1451428_x_at | Gli2 | 0.45404282 |
| 1441909_s_at | 1700027N10Rik | 0.45449194 |
| 1429367_at | LOC100039795 | 0.45453373 |
| 1433914_at | Hspb1 | 0.4548335 |
| 1427760_s_at | --- | 0.45512044 |
| 1421469_a_at | Emp3 | 0.45605782 |
| 1460365_a_at | C230093N12Rik | 0.4560809 |
| 1431751_a_at | 9030425L15Rik | 0.4577028 |
| 1440388_at | Thsd1 | 0.45781997 |
| 1435237_at | Socs3 | 0.45878938 |
| 1430549_at | Slc5a7 | 0.45887697 |
| 1454909_at | Hdac11 | 0.45891428 |
| 1429712_at | Yipf5 | 0.45981354 |
| 1456217_at | Cdyl | 0.46040925 |
| 1453853_a_at | Nxn | 0.4615065 |
| 1457632_s_at | Neto2 | 0.46157432 |
| 1450448_at | Gal3st4 | 0.46162125 |
| 1435032_at | Zfp521 | 0.4623097 |
| 1436362_x_at | --- | 0.462386 |
| 1416342_at | --- | 0.46267715 |
| 1449845_a_at | --- | 0.46380353 |
| 1440831_at | D4Bwg0951e | 0.4638994 |
| 1446656_at | Htra1 | 0.4639204 |
| 1448618_at | H2afy2 | 0.46403238 |
| 1418674_at | Mmab | 0.46416712 |
| 1448428_at | Gpc1 | 0.4647156 |
| 1436197_at | Arrdc4 | 0.46482247 |
| 1415855_at | Nrip1 | 0.4659794 |
| 1417923_at | Htra1 | 0.46661678 |
| 1428808_at | Gpr39 | 0.4669353 |
| 1449310_at | --- | 0.46723023 |
| 1428646_at | Ebf1 | 0.46768486 |
| 1436003_at | Nlrx1 | 0.46773067 |
| 1430310_at | Thra | 0.46780524 |
| 1416474_at | Lpar1 | 0.46786904 |
| 1423996_a_at | Ephb4 | 0.46824756 |
| 1417439_at | Als2cl | 0.46826708 |
| 1435458_at | Dhcr24 | 0.46852276 |
| 1455744_at | Rspo2 | 0.46865368 |
| 1434274_at | Macrod1 | 0.46911013 |
| 1457780_at | Gm98 | 0.46955362 |
| 1435193_at | Twist2 | 0.46978906 |
| 1435047_at | OTTMUSG00000009332 | 0.4711717 |
| 1451173_at | Socs3 | 0.47149983 |
| 1416491_at | Naga | 0.47266498 |
| 1455956_x_at | Sgip1 | 0.47266674 |
| 1415806_at | Trp53inp2 | 0.4726941 |
| 1441030_at | Polb | 0.47271225 |
| 1419674_a_at | Chst12 | 0.47316155 |
| 1420754_at | Sdk1 | 0.47326607 |
| 1435943_at | Fkbp14 | 0.4732954 |
| 1435514_at | Gpr137b | 0.47335562 |
| 1457675_at | St3gal1 | 0.4735587 |
| 1426774_at | Il13ra1 | 0.47386867 |
| 1450652_at | Socs3 | 0.47401658 |
| 1460259_s_at | B630019K06Rik | 0.47432548 |
| 1452357_at | Epha1 | 0.4747073 |
| 1435488_at | --- | 0.4758259 |
| 1435146_s_at | Loxl1 | 0.47618815 |
| 1420500_at | Htra3 | 0.47658548 |
| 1433558_at | --- | 0.47694343 |
| 1448890_at | --- | 0.47713646 |
| 1420499_at | Il6st | 0.47721112 |
| 1434479_at | D1Ertd471e | 0.47724542 |
| 1444797_at | Ccnd2 | 0.47798467 |
| 1455244_at | Tmem141 | 0.47800204 |
| 1416754_at | Nek6 | 0.47821975 |
| 1441843_s_at | Fbxl2 | 0.47851738 |
| 1422629_s_at | Ccnd2 | 0.4788543 |
| 1434123_at | --- | 0.47935113 |
| 1426074_at | B4galt4 | 0.47950625 |
| 1436966_at | Add3 | 0.4802529 |
| 1423047_at | Tmem53 | 0.48067996 |
| 1419811_at | Dhcr7 | 0.48099655 |
| 1427278_at | Dock4 | 0.4810108 |
| 1435153_at | Rtp4 | 0.4812551 |
| 1460196_at | 5230400M06Rik | 0.48158866 |
| 1449335_at | Cacng8 | 0.48177359 |
| 1431810_a_at | OTTMUSG00000000421 | 0.4818732 |
| 1427885_at | Creld1 | 0.48193696 |
| 1426539_at | Aldh1b1 | 0.48208997 |
| 1418512_at | A430107O13Rik | 0.48240703 |
| 1424304_at | Plat | 0.48241705 |
| 1427051_at | Prickle1 | 0.4827472 |
| 1426225_at | 2410081M15Rik | 0.48335856 |
| 1432400_at | Zmynd15 | 0.48416466 |
| 1417836_at | 2810021J22Rik | 0.4842918 |
| 1447861_x_at | Rab27b | 0.48449752 |
| 1439834_at | Hfe | 0.4851178 |
| 1438796_at | Bscl2 | 0.48538113 |
| 1417813_at | Nope | 0.48558617 |
| 1436501_at | Tmem185b | 0.48579404 |
| 1442531_at | Gpr124 | 0.4858291 |
| 1437130_at | Prkd2 | 0.48591256 |
| 1454796_at | A030001D20Rik | 0.48624995 |
| 1437056_x_at | Arfgap3 | 0.4867234 |
| 1444450_at | Sirpa | 0.48716077 |
| 1439926_at | Ptprj | 0.48724875 |
| 1419088_at | 1810054D07Rik | 0.48745647 |
| 1438861_at | LOC100040519 | 0.48758942 |
| 1453020_at | Dab2 | 0.48765528 |
| 1423365_at | Adck4 | 0.48899755 |
| 1425894_at | Mageh1 | 0.48899806 |
| 1435072_at | Hspb2 | 0.4890805 |
| 1437256_at | Pik3r1 | 0.48939297 |
| 1430392_at | Egfr | 0.4895455 |
| 1419687_at | Ndg2 | 0.4903063 |
| 1436729_at | 4733401H18Rik | 0.49041736 |
| 1423926_at | --- | 0.49053076 |
| 1436387_at | Klhl24 | 0.4905421 |
| 1423372_at | AA763515 | 0.4909598 |
| 1452664_a_at | Pik3r1 | 0.49168342 |
| 1434378_a_at | Slc43a1 | 0.4919676 |
| 1429030_at | Auh | 0.49210823 |
| 1426277_at | Mylip | 0.49247548 |
| 1434089_at | Tmem154 | 0.49296063 |
| 1417801_a_at | Tspan11 | 0.49315226 |
| 1447784_x_at | Rnd3 | 0.49370027 |
| 1424670_s_at | Adk | 0.49390864 |
| 1416401_at | 2810047C21Rik | 0.493993 |
| 1445107_at | Cnp | 0.49442887 |
| 1422641_at | 1700112E06Rik | 0.49452326 |
| 1455822_x_at | --- | 0.49468452 |
| 1451412_a_at | --- | 0.49478137 |
| 1453698_at | Chmp2a | 0.49553227 |
| 1448259_at | Ccnd2 | 0.49553344 |
| 1418762_at | Dmd | 0.49558014 |
| 1418072_at | Dusp10 | 0.49570325 |
| 1417883_at | Dkk3 | 0.49593493 |
| 1428402_at | BC030046 | 0.4962699 |
| 1417163_at | Bcl6 | 0.49668196 |
| 1430886_at | Tpcn2 | 0.497018 |
| 1446990_at | Entpd5 | 0.49740294 |
| 1435504_at | Impact | 0.49741408 |
| 1418133_at | Fads3 | 0.49836195 |
| 1455297_at | Peli2 | 0.49882874 |
| 1421425_a_at | --- | 0.4988535 |
| 1436614_at | Kctd12b | 0.49935144 |
| 1438798_at | Zfp629 | 0.49951062 |
| 1416452_at | --- | 0.49952522 |
| 1453241_a_at | Lpar1 | 0.49999315 |
| 1443088_at | Gsdmd | 0.5000721 |
| 1425114_at | Fndc4 | 0.5001826 |
| 1424217_at | EG621083 | 0.5002378 |
| 1424625_a_at | A830007P12Rik | 0.5004255 |
| 1460619_at | Slc35a2 | 0.500727 |
| 1450642_at | Gstt2 | 0.5008763 |
| 1437442_at | Cdyl | 0.5010693 |
| 1429313_at | Serpinf1 | 0.5016617 |
| 1455899_x_at | Serf2 | 0.5017582 |
| 1418933_at | Pitpnc1 | 0.5020855 |
| 1455073_at | Gprc5b | 0.5024307 |
| 1436085_at | Eef2k | 0.50246215 |
| 1418400_at | Tcf3 | 0.50252324 |
| 1441368_at | ENSMUSG00000057924 | 0.5025453 |
| 1436841_at | Rnase4 | 0.5027309 |
| 1424679_at | Cebpd | 0.5029857 |
| 1449438_at | Tnnt1 | 0.5031669 |
| 1456554_at | Daam1 | 0.5037541 |
| 1419359_at | D4Wsu53e | 0.5040421 |
| 1454746_at | --- | 0.5042218 |
| 1435603_at | Gpx7 | 0.50427455 |
| 1435910_at | Sc5d | 0.50467956 |
| 1452922_at | Prrg3 | 0.50491446 |
| 1454900_s_at | D330022A01Rik | 0.50494075 |
| 1448460_at | Hoxa10 | 0.5050925 |
| 1437534_at | Guca1a | 0.5054165 |
| 1439293_at | Ypel2 | 0.5057072 |
| 1425587_a_at | Fads3 | 0.5057654 |
| 1449670_x_at | Zfp518b | 0.5061167 |
| 1436673_at | Slit2 | 0.5061544 |
| 1418752_at | D4Wsu53e | 0.5068982 |
| 1441776_at | Smad6 | 0.5069064 |
| 1454711_at | Rnf125 | 0.50712055 |
| 1454931_at | AW555464 | 0.5071646 |
| 1454048_a_at | Ankrd29 | 0.5075795 |
| 1436994_a_at | Phyh | 0.5075807 |
| 1418156_at | Rnpepl1 | 0.50782275 |
| 1459840_s_at | Fbxl20 | 0.5078278 |
| 1427165_at | Il11ra1 | 0.5082223 |
| 1435084_at | C77713 | 0.50831896 |
| 1429348_at | --- | 0.5084853 |
| 1447993_a_at | Bnc2 | 0.5090667 |
| 1424797_a_at | Serpinb9 | 0.5092568 |
| 1456329_at | Anxa8 | 0.509268 |
| 1425383_a_at | Hddc3 | 0.5094351 |
| 1420498_a_at | Acat1 | 0.5098115 |
| 1451181_at | Osmr | 0.50981885 |
| 1455440_at | Ccdc28b | 0.5098635 |
| 1433668_at | LOC640441 | 0.5103522 |
| 1435133_at | Zfp422 | 0.51058716 |
| 1417022_at | Nrip1 | 0.51066244 |
| 1450004_at | --- | 0.5113389 |
| 1419823_s_at | Hoxc8 | 0.5113503 |
| 1434333_a_at | Serpinf1 | 0.51144844 |
| 1450767_at | Dnaja4 | 0.5116182 |
| 1448606_at | Nupr1 | 0.51178914 |
| 1424762_at | Irak4 | 0.51229477 |
| 1453724_a_at | Ficd | 0.512354 |
| 1434196_at | --- | 0.51262784 |
| 1425269_at | AI450540 | 0.5130003 |
| 1447844_at | 1700008J07Rik | 0.5130505 |
| 1416321_s_at | Shroom3 | 0.51338524 |
| 1416832_at | --- | 0.51345015 |
| 1423371_at | Frmd6 | 0.5140447 |
| 1418586_at | Camkk2 | 0.51484305 |
| 1428732_at | Abtb2 | 0.51490957 |
| 1449368_at | Lhfpl2 | 0.5151704 |
| 1416637_at | Lhfpl2 | 0.515854 |
| 1425525_a_at | Npc2 | 0.51586324 |
| 1428391_at | Nrip1 | 0.5158948 |
| 1419132_at | Hsdl2 | 0.516213 |
| 1433551_at | Rbm12b | 0.516254 |
| 1418186_at | Snx33 | 0.5162772 |
| 1432136_s_at | Tusc1 | 0.5163289 |
| 1419415_a_at | Zfp36l1 | 0.51670134 |
| 1427093_at | Mmp19 | 0.5167172 |
| 1433453_a_at | Add3 | 0.5171524 |
| 1451405_at | Alpl | 0.5175819 |
| 1433855_at | Prkg2 | 0.517902 |
| 1433500_at | Zfp362 | 0.51850426 |
| 1436189_at | Glb1l | 0.5187022 |
| 1451983_at | Pigh | 0.51872516 |
| 1431226_a_at | Cdc42bpg | 0.51879656 |
| 1433523_at | 5730593F17Rik | 0.51892567 |
| 1438971_x_at | Mtmr7 | 0.51912755 |
| 1434153_at | Cd1d1 | 0.5191648 |
| 1437765_at | Jak2 | 0.5191803 |
| 1435982_at | Pdzrn3 | 0.51935315 |
| 1438202_at | Zfp707 | 0.51977444 |
| 1458074_at | Pitx1 | 0.5200741 |
| 1426210_x_at | --- | 0.5207808 |
| 1444585_at | Egfr | 0.5208869 |
| 1454755_at | Gale | 0.5210984 |
| 1448889_at | Lpin1 | 0.5212032 |
| 1418932_at | --- | 0.5213031 |
| 1440342_at | 1110032A03Rik | 0.5214048 |
| 1455372_at | Atoh8 | 0.52146614 |
| 1431988_at | Lrrc49 | 0.52215147 |
| 1452889_at | 1810021J13Rik | 0.52221686 |
| 1457731_at | Rxra | 0.522498 |
| 1424613_at | 8430426H19Rik | 0.5226506 |
| 1416355_at | Rab7l1 | 0.52269715 |
| 1419182_at | --- | 0.52276444 |
| 1442620_at | LOC676870 | 0.52284163 |
| 1427279_at | H6pd | 0.52288747 |
| 1456344_at | --- | 0.5231521 |
| 1451643_a_at | Tspan11 | 0.52326465 |
| 1426368_at | Ccnd2 | 0.5238564 |
| 1449145_a_at | --- | 0.5250441 |
| 1447864_s_at | --- | 0.5250609 |
| 1436842_at | Abtb2 | 0.52540296 |
| 1437685_x_at | 1110003E01Rik | 0.52572244 |
| 1451253_at | Col12a1 | 0.5257612 |
| 1459170_at | Hexa | 0.5261747 |
| 1429822_at | Thbs1 | 0.52625 |
| 1448792_a_at | Stom | 0.5264949 |
| 1428761_a_at | 9030611O19Rik | 0.52667236 |
| 1420516_at | Serpinb6b | 0.5267254 |
| 1435464_at | B130040O20Rik | 0.52723134 |
| 1451932_a_at | Prkg2 | 0.52761984 |
| 1418591_at | Akap2 | 0.5276483 |
| 1446304_at | Tm7sf2 | 0.5279266 |
| 1426775_s_at | --- | 0.5280295 |
| 1419654_at | 5031439G07Rik | 0.52832764 |
| 1440192_at | Bcl3 | 0.52844036 |
| 1415894_at | Snupn | 0.52891713 |
| 1433610_at | Galnt12 | 0.5289362 |
| 1425505_at | Hist1h1c | 0.5292678 |
| 1453773_at | Rtkn2 | 0.52936614 |
| 1423506_a_at | Ralgps2 | 0.5295636 |
| 1417214_at | Rab3c | 0.5298315 |
| 1448687_at | Mettl7a1 | 0.53097713 |
| 1424655_at | Gdnf | 0.53140336 |
| 1444704_at | 4931406P16Rik | 0.53157806 |
| 1437318_at | --- | 0.53201485 |
| 1423295_at | Igf2bp3 | 0.5321811 |
| 1440972_at | Emilin2 | 0.5321908 |
| 1434796_at | Tnfaip8 | 0.53251034 |
| 1450663_at | --- | 0.53264034 |
| 1437347_at | 5730508B09Rik | 0.53284603 |
| 1425528_at | Hsd17b7 | 0.53338516 |
| 1447948_at | Eno3 | 0.53347045 |
| 1451537_at | Prkd2 | 0.5336067 |
| 1448818_at | 3110070M22Rik | 0.5337139 |
| 1419332_at | Ccpg1 | 0.533935 |
| 1426833_at | 2210408K08Rik | 0.5339951 |
| 1424082_at | Ccnd2 | 0.53428847 |
| 1423473_at | A430107O13Rik | 0.53429806 |
| 1417853_at | Ppp1r12b | 0.53433645 |
| 1433742_at | Osmr | 0.5346533 |
| 1423306_at | Loh11cr2a | 0.5353385 |
| 1428252_at | Cd248 | 0.5353412 |
| 1449252_at | Actr1b | 0.53572774 |
| 1428942_at | Cml1 | 0.5357658 |
| 1451236_at | Prrx1 | 0.5360045 |
| 1449876_at | Daam1 | 0.5360137 |
| 1416981_at | Mgst1 | 0.53608495 |
| 1428427_at | Daglb | 0.53610814 |
| 1418807_at | Foxo1 | 0.5362952 |
| 1417860_a_at | Zbtb34 | 0.53645617 |
| 1416136_at | Tchh | 0.5365008 |
| 1448952_at | --- | 0.5367352 |
| 1416753_at | Trim47 | 0.5368864 |
| 1419427_at | 2-Sep | 0.5370115 |
| 1457119_at | Dnm1 | 0.537061 |
| 1433826_at | Bdkrb1 | 0.53723365 |
| 1433691_at | 5430435G22Rik | 0.53729427 |
| 1421869_at | Prrx1 | 0.5374252 |
| 1434982_at | Gadd45g | 0.5374704 |
| 1417185_at | Zbtb4 | 0.53758174 |
| 1439539_at | Stard5 | 0.5376596 |
| 1433745_at | Stom | 0.53832656 |
| 1429258_at | Map4k4 | 0.53835386 |
| 1441506_at | ENSMUSG00000073981 | 0.5384628 |
| 1419376_at | --- | 0.5385469 |
| 1451518_at | Klhl24 | 0.53861576 |
| 1415961_at | Cyp2j6 | 0.53875065 |
| 1427143_at | Nupr1 | 0.53945076 |
| 1438426_at | 1700029I01Rik | 0.5402179 |
| 1429593_at | 1300014I06Rik | 0.54035294 |
| 1416123_at | Dact3 | 0.54076445 |
| 1437303_at | Igf2bp2 | 0.54120064 |
| 1457128_at | Rdh11 | 0.5412228 |
| 1436589_x_at | Commd9 | 0.54134417 |
| 1435089_at | Lipa | 0.5413805 |
| 1451426_at | Zfyve1 | 0.5416094 |
| 1422603_at | Rom1 | 0.54196364 |
| 1419006_s_at | A030009H04Rik | 0.5420274 |
| 1436293_x_at | Tmem136 | 0.54234177 |
| 1428155_at | Masp1 | 0.5428541 |
| 1435029_at | --- | 0.543284 |
| 1423805_at | C130015C19 | 0.5435992 |
| 1455687_at | --- | 0.5437149 |
| 1435748_at | Nek6 | 0.54387563 |
| 1456778_at | P2ry6 | 0.543877 |
| 1456341_a_at | Map4k4 | 0.54388636 |
| 1449124_at | --- | 0.54410684 |
| 1460255_at | --- | 0.5441772 |
| 1423312_at | --- | 0.5446521 |
| 1448201_at | Pitpnc1 | 0.5451702 |
| 1441870_s_at | D130051D11Rik | 0.54525363 |
| 1425789_s_at | Caprin1 | 0.5452611 |
| 1425145_at | Ctso | 0.54624194 |
| 1460251_at | Ap3m2 | 0.546504 |
| 1433887_at | --- | 0.54655266 |
| 1443906_at | Adal | 0.54708993 |
| 1427178_at | Fbln5 | 0.54773384 |
| 1455346_at | Pdgfrb | 0.54816186 |
| 1450351_a_at | Ifitm2 | 0.5481631 |
| 1435540_at | Rabl2a | 0.54878306 |
| 1424932_at | Tbcel | 0.54907334 |
| 1435866_s_at | 2010203O07Rik | 0.54908186 |
| 1447706_at | Stc1 | 0.54951906 |
| 1437052_s_at | --- | 0.5495612 |
| 1452059_at | --- | 0.54957366 |
| 1423794_at | --- | 0.5496624 |
| 1454733_at | Dusp10 | 0.5497575 |
| 1454677_at | Nhlrc1 | 0.5502821 |
| 1452296_at | BC046404 | 0.5503179 |
| 1433651_at | Ece1 | 0.5506003 |
| 1434150_a_at | Scamp1 | 0.5508217 |
| 1460700_at | --- | 0.55097735 |
| 1457832_at | Pak3 | 0.55140924 |
| 1422720_at | Phf21a | 0.5515776 |
| 1422465_a_at | Higd1c | 0.5518414 |
| 1434745_at | Slc38a9 | 0.55185074 |
| 1451978_at | Csad | 0.5520267 |
| 1447018_at | Zmym6 | 0.55226994 |
| 1437833_at | Ndrg1 | 0.55262357 |
| 1425510_at | Prkar1b | 0.55280596 |
| 1425214_at | Irak4 | 0.5528698 |
| 1433617_s_at | Akap2 | 0.55340654 |
| 1435857_s_at | ENSMUSG00000074630 | 0.5537489 |
| 1422567_at | --- | 0.5539822 |
| 1459183_at | Sesn3 | 0.55439305 |
| 1436761_s_at | --- | 0.5545942 |
| 1441076_at | Ttc30b | 0.5546775 |
| 1443952_at | Mvp | 0.55533546 |
| 1456611_at | Acaa1a | 0.5559265 |
| 1428776_at | Gpr137b | 0.5559468 |
| 1416551_at | Oat | 0.55598813 |
| 1425896_a_at | 4833442J19Rik | 0.5560613 |
| 1451229_at | Slc39a8 | 0.55629337 |
| 1419164_at | --- | 0.5567749 |
| 1424140_at | Slit2 | 0.5567883 |
| 1435147_x_at | 1810013D10Rik | 0.5568258 |
| 1448754_at | Rab8b | 0.5570677 |
| 1424659_at | Tmc7 | 0.5572132 |
| 1417070_at | Mapk8ip3 | 0.55723345 |
| 1417638_at | Bambi | 0.5574864 |
| 1450731_s_at | Dcun1d5 | 0.5578598 |
| 1457464_at | Hipk3 | 0.5581334 |
| 1452894_at | Rarg | 0.5582085 |
| 1425985_s_at | --- | 0.5583302 |
| 1452939_a_at | EG433144 | 0.5583654 |
| 1417461_at | Ube2h | 0.55847186 |
| 1419015_at | Bckdha | 0.5585514 |
| 1435752_s_at | Nfia | 0.55867666 |
| 1424987_at | 5033421C21Rik | 0.5588702 |
| 1452249_at | Mtss1 | 0.5590159 |
| 1443122_at | --- | 0.5592389 |
| 1418057_at | Tmem100 | 0.55926204 |
| 1439774_at | LOC100048079 | 0.55929756 |
| 1416950_at | Lpar1 | 0.55967104 |
| 1442325_at | D7Ertd715e | 0.55978304 |
| 1449143_at | 4931406P16Rik | 0.5598123 |
| 1423074_at | Trim62 | 0.5604208 |
| 1449083_at | Tns1 | 0.5604612 |
| 1437070_at | Prrx1 | 0.56083906 |
| 1429590_at | --- | 0.56085426 |
| 1428550_at | Car5b | 0.56099296 |
| 1448250_at | Pitpnc1 | 0.56178975 |
| 1460295_s_at | Mamdc2 | 0.5619109 |
| 1428626_at | Lmod1 | 0.5619271 |
| 1452650_at | Adamts4 | 0.5623716 |
| 1418002_at | Bmpr2 | 0.56242067 |
| 1424713_at | Tcta | 0.56261486 |
| 1426858_at | Slc7a3 | 0.5627081 |
| 1460242_at | Scamp1 | 0.5627384 |
| 1430074_x_at | Slc38a2 | 0.5628876 |
| 1415903_at | Fbxl20 | 0.56315196 |
| 1455214_at | Unc13b | 0.56321305 |
| 1439755_at | Pld3 | 0.56347084 |
| 1456087_at | --- | 0.56357354 |
| 1430183_at | Ephb3 | 0.56362844 |
| 1416808_at | D930014E17Rik | 0.5637463 |
| 1429031_at | B430007K19Rik | 0.56388766 |
| 1426418_at | Dnajc1 | 0.5640248 |
| 1422631_at | Ypel2 | 0.5641022 |
| 1437689_x_at | Tcf3 | 0.5645663 |
| 1416567_s_at | Ifnar2 | 0.56486386 |
| 1435264_at | Ptprj | 0.5653967 |
| 1440799_s_at | Pkig | 0.5661202 |
| 1427126_at | Bmper | 0.5664514 |
| 1438756_at | Gpr146 | 0.5664657 |
| 1439506_at | Pdgfrb | 0.5670784 |
| 1447093_at | 2510002D24Rik | 0.5672589 |
| 1426403_at | --- | 0.5675614 |
| 1437279_x_at | Hist1h1c | 0.56778276 |
| 1416708_a_at | Col5a1 | 0.5683132 |
| 1441679_at | Ndrg1 | 0.5683326 |
| 1428791_at | Stx11 | 0.5683812 |
| 1442322_at | Ppm1a | 0.5685937 |
| 1421870_at | Enox1 | 0.56859696 |
| 1439747_at | Tert | 0.5687405 |
| 1418269_at | Nfatc4 | 0.56878096 |
| 1445326_at | Ldhb | 0.5690371 |
| 1434277_a_at | Acat1 | 0.569305 |
| 1430135_at | Sbsn | 0.5693233 |
| 1446929_at | Snapc1 | 0.569528 |
| 1440132_s_at | Enpp4 | 0.5702685 |
| 1436584_at | Limk1 | 0.5704176 |
| 1427981_a_at | Nagk | 0.57044697 |
| 1415944_at | Napa | 0.5704718 |
| 1421392_a_at | Ubfd1 | 0.57059646 |
| 1444396_at | D12Ertd123e | 0.5707657 |
| 1448950_at | --- | 0.57077116 |
| 1457368_at | Slit2 | 0.5708333 |
| 1448751_at | Mfsd10 | 0.5708617 |
| 1418379_s_at | 9530086O07Rik | 0.5713416 |
| 1433723_s_at | D13Ertd666e | 0.5716835 |
| 1425210_s_at | Sod2 | 0.57169724 |
| 1426607_at | 4931406P16Rik | 0.5716998 |
| 1421428_at | Smox | 0.571875 |
| 1420124_s_at | Man2c1 | 0.57188904 |
| 1457260_at | Plcd1 | 0.57214195 |
| 1418854_at | Spred1 | 0.57225335 |
| 1454942_at | Slc6a17 | 0.57272357 |
| 1416124_at | Slc12a6 | 0.5732745 |
| 1436195_at | 9330128J19Rik | 0.5733826 |
| 1459991_at | 4930431B09Rik | 0.5737005 |
| 1425509_at | Egfl7 | 0.5737167 |
| 1448729_a_at | Ccpg1 | 0.5747135 |
| 1441513_at | Nid1 | 0.5747749 |
| 1443253_at | EG434402 | 0.5748985 |
| 1427164_at | Adamtsl4 | 0.5751184 |
| 1439395_at | D7Ertd715e | 0.575238 |
| 1437181_at | Ndrg1 | 0.57588005 |
| 1427020_at | BC011487 | 0.5759096 |
| 1446921_at | Rbbp6 | 0.5762078 |
| 1427202_at | Zfp36 | 0.5767676 |
| 1419869_s_at | Sema6d | 0.57690275 |
| 1418675_at | Cdc14b | 0.57704663 |
| 1455859_at | Tcta | 0.57705325 |
| 1416168_at | Fndc4 | 0.57734007 |
| 1452130_at | H2afy2 | 0.5777324 |
| 1452907_at | Col5a1 | 0.577893 |
| 1439368_a_at | Ndrg1 | 0.57806057 |
| 1427620_at | Tbc1d23 | 0.5781994 |
| 1449043_at | Mt2 | 0.57827306 |
| 1454685_at | Ebf1 | 0.57846624 |
| 1439849_at | Abhd5 | 0.57847816 |
| 1423876_at | Bsdc1 | 0.57885146 |
| 1432080_s_at | Stard5 | 0.5790297 |
| 1449561_at | Bmpr2 | 0.5793026 |
| 1418455_at | Tbcel | 0.5798481 |
| 1426722_at | Ttc28 | 0.57996684 |
| 1423865_at | 1200015N20Rik | 0.5803152 |
| 1428098_a_at | Ptprf | 0.5803357 |
| 1447100_s_at | Copg | 0.5805669 |
| 1459026_at | A930037G23Rik | 0.5806742 |
| 1427550_at | Gpsm3 | 0.5811538 |
| 1429032_at | Sipa1l1 | 0.58129823 |
| 1417952_at | Pim1 | 0.58132434 |
| 1433891_at | Stard5 | 0.58142585 |
| 1426925_at | Olfm1 | 0.58143705 |
| 1452093_at | Leprel2 | 0.58197206 |
| 1440153_at | Tshz1 | 0.5822098 |
| 1416430_at | Papss2 | 0.58250076 |
| 1416322_at | EG433144 | 0.58300656 |
| 1438579_at | Tram2 | 0.58311796 |
| 1448407_at | Jak2 | 0.58312875 |
| 1452826_s_at | 2610014I16Rik | 0.58325887 |
| 1449005_at | Usp11 | 0.5832693 |
| 1447359_at | Timp2 | 0.58330435 |
| 1425280_at | Prrx1 | 0.5833321 |
| 1423768_at | Dnajc3a | 0.58350796 |
| 1423261_at | Nr4a3 | 0.5835116 |
| 1419616_at | Rab4b | 0.5837571 |
| 1424761_at | 2700062C07Rik | 0.5837615 |
| 1444015_at | Wdr5b | 0.5837883 |
| 1454789_x_at | Sod2 | 0.5838814 |
| 1422771_at | Slc35a2 | 0.5840543 |
| 1431837_at | Klhl29 | 0.5841701 |
| 1429270_a_at | Tpbg | 0.5842531 |
| 1453196_a_at | Gpr137b-ps | 0.5842648 |
| 1428064_at | Ccdc28b | 0.5843357 |
| 1436987_at | 4931406I20Rik | 0.5845007 |
| 1449219_at | D14Abb1e | 0.58451515 |
| 1435529_at | Khk | 0.58453524 |
| 1423632_at | Klf2 | 0.5845562 |
| 1441977_at | Tgfb1 | 0.5846134 |
| 1424400_a_at | Clip1 | 0.5846426 |
| 1418097_a_at | Oxsm | 0.58470374 |
| 1434924_at | Acss2 | 0.5847675 |
| 1440650_at | Zfp790 | 0.5847785 |
| 1435900_at | --- | 0.5848335 |
| 1436495_s_at | Slc11a2 | 0.58496547 |
| 1416463_at | Ksr1 | 0.58510727 |
| 1429506_at | Actn3 | 0.5853276 |
| 1434507_at | --- | 0.58534 |
| 1433901_at | Pole4 | 0.58547133 |
| 1454783_at | Itpkc | 0.5854751 |
| 1415911_at | Pak3 | 0.5859181 |
| 1440013_at | 2010002N04Rik | 0.58597225 |
| 1436221_at | 1810058I24Rik | 0.5860715 |
| 1450040_at | D930026N18Rik | 0.5861714 |
| 1451461_a_at | Nod1 | 0.58621037 |
| 1449227_at | Tssc1 | 0.58641076 |
| 1441942_x_at | Gltscr1 | 0.5864625 |
| 1426734_at | Lipa | 0.5867651 |
| 1451550_at | Loxl1 | 0.58685243 |
| 1418476_at | Leng1 | 0.5874742 |
| 1436436_at | Mansc1 | 0.58762956 |
| 1426623_a_at | Grasp | 0.5876436 |
| 1425934_a_at | Pvrl2 | 0.5877126 |
| 1451904_a_at | 1810054D07Rik | 0.58776075 |
| 1456053_at | P2rx4 | 0.5877764 |
| 1435465_at | Nek6 | 0.58801615 |
| 1416673_at | St6galnac4 | 0.58816636 |
| 1417193_at | Prtg | 0.58824044 |
| 1428862_at | 2310005E10Rik | 0.588247 |
| 1452009_at | Has1 | 0.5884022 |
| 1452973_at | Arhgap1 | 0.5885779 |
| 1417903_at | --- | 0.5886471 |
| 1455893_at | Klc3 | 0.5888532 |
| 1416273_at | Clip4 | 0.58885604 |
| 1448986_x_at | --- | 0.5890643 |
| 1431079_at | --- | 0.5896349 |
| 1447830_s_at | Psip1 | 0.5896701 |
| 1419292_at | LOC100039284 | 0.5897404 |
| 1442916_at | Zfp324 | 0.5900134 |
| 1426441_at | Pik3r1 | 0.5900249 |
| 1442196_at | Rilpl1 | 0.5901125 |
| 1417850_at | Arhgef12 | 0.5902783 |
| 1428437_at | Hexim1 | 0.5904745 |
| 1428650_at | 4930402C16Rik | 0.5906965 |
| 1429012_at | Bach1 | 0.59094334 |
| 1440796_at | Mvd | 0.59097165 |
| 1443153_at | Slc12a6 | 0.5910501 |
| 1448835_at | A530064N14Rik | 0.5911144 |
| 1421571_a_at | 1810054D07Rik | 0.5912332 |
| 1448490_at | Spred1 | 0.5912563 |
| 1455741_a_at | 4933431E20Rik | 0.59220386 |
| 1437341_x_at | Vldlr | 0.59244746 |
| 1450687_at | Ube2h | 0.5925762 |
| 1418666_at | B4galt5 | 0.59260213 |
| 1436063_at | Zfp260 | 0.5926276 |
| 1422528_a_at | 2010111I01Rik | 0.5927643 |
| 1417483_at | Rbmx | 0.59284765 |
| 1439630_x_at | 2010107G23Rik | 0.5929464 |
| 1439181_at | Snx32 | 0.5932118 |
| 1460206_at | Saa2 | 0.59328276 |
| 1429244_at | LOC100046232 | 0.59368235 |
| 1436150_at | 2310051E17Rik | 0.59371597 |
| 1434734_at | Clcn6 | 0.59373355 |
| 1439256_x_at | --- | 0.5940425 |
| 1426871_at | Lancl3 | 0.59412676 |
| 1458140_at | Dlg2 | 0.5941435 |
| 1438985_x_at | BC031353 | 0.59418935 |
| 1426440_at | Il17d | 0.5942477 |
| 1436515_at | Fstl1 | 0.59434247 |
| 1430030_at | Stat5a | 0.59452575 |
| 1433661_at | 7530414M10Rik | 0.5949933 |
| 1419247_at | 2610020H08Rik | 0.5950818 |
| 1444646_at | Apcdd1 | 0.5952994 |
| 1427747_a_at | 3-Mar | 0.59537345 |
| 1423284_at | Ldoc1l | 0.595386 |
| 1452445_at | LOC629952 | 0.59546405 |
| 1451191_at | Higd2a | 0.5955681 |
| 1437252_at | Slc35f5 | 0.5956316 |
| 1418108_at | C030011O14Rik | 0.5957124 |
| 1426867_at | Ndufab1 | 0.59572375 |
| 1418396_at | Ralgps2 | 0.59576094 |
| 1439965_at | --- | 0.59589684 |
| 1452312_at | Lztfl1 | 0.5960689 |
| 1418402_at | Sbsn | 0.59616655 |
| 1418288_at | D930005D10Rik | 0.59647334 |
| 1432466_a_at | Epha1 | 0.5965226 |
| 1424890_at | Pnrc1 | 0.59664816 |
| 1435213_at | Mapkapk3 | 0.5966634 |
| 1448050_s_at | Stoml1 | 0.59682524 |
| 1452284_at | Irgq | 0.59701586 |
| 1423075_at | 1200015N20Rik | 0.59733516 |
| 1436537_at | Rarg | 0.5973949 |
| 1424474_a_at | Rbak | 0.5974666 |
| 1436326_at | Trip11 | 0.5974999 |
| 1448391_at | Cend1 | 0.59755075 |
| 1426970_a_at | --- | 0.5976218 |
| 1440400_at | Zfyve1 | 0.5976835 |
| 1454901_at | Slc41a2 | 0.59842986 |
| 1453747_at | Masp1 | 0.5986251 |
| 1426535_at | --- | 0.59889585 |
| 1418071_s_at | Meis2 | 0.5989705 |
| 1422317_a_at | Glb1l | 0.59903127 |
| 1417211_a_at | Zdhhc24 | 0.59926474 |
| 1427406_at | Megf8 | 0.5993848 |
| 1421987_at | Creb3 | 0.59943765 |
| 1441108_at | Zkscan1 | 0.5995025 |
| 1449520_at | D430015B01Rik | 0.59954304 |
| 1436894_at | Emb | 0.5995979 |
| 1435285_at | Atp5e | 0.5999327 |
| 1434193_at | Nxn | 0.60007083 |
| 1435087_at | Slc9a3r2 | 0.60011005 |
| 1418936_at | Stat3 | 0.60019606 |
| 1434283_at | 1810054D07Rik | 0.60023296 |
| 1434639_at | Ncald | 0.60073745 |
| 1415857_at | Sytl2 | 0.60128456 |
| 1419668_at | Ppp1r3c | 0.60136837 |
| 1426081_a_at | Ldhb | 0.6017112 |
| 1428500_at | 2400009B08Rik | 0.6020558 |
| 1423438_at | Thbs4 | 0.60211766 |
| 1434439_at | Gpr146 | 0.6021535 |
| 1448825_at | Atp2a2 | 0.6025747 |
| 1430385_a_at | Zdhhc4 | 0.60267943 |
| 1447952_at | Pitpnc1 | 0.6028505 |
| 1431505_at | Afap1 | 0.60298467 |
| 1424382_at | 4632404H12Rik | 0.60304147 |
| 1419042_at | Kbtbd11 | 0.60311615 |
| 1449209_a_at | 4633401B06Rik | 0.6033558 |
| 1451314_a_at | Tgfbr3 | 0.6033721 |
| 1433506_at | Rell1 | 0.6035404 |
| 1455688_at | Ahnak2 | 0.60356176 |
| 1437846_x_at | Tacc1 | 0.6035769 |
| 1439833_at | Tcta | 0.6036104 |
| 1421670_a_at | Ydjc | 0.60370344 |
| 1419514_at | Ext1 | 0.6037939 |
| 1448610_a_at | Higd1a | 0.6038715 |
| 1426293_at | Boc | 0.6039503 |
| 1421162_a_at | Emb | 0.60396975 |
| 1425514_at | Zfp36l1 | 0.60425353 |
| 1415989_at | Eml1 | 0.60432065 |
| 1449326_x_at | --- | 0.6043729 |
| 1423297_at | Harbi1 | 0.6044528 |
| 1451446_at | Armcx1 | 0.60449195 |
| 1435385_at | C530044N13Rik | 0.6047485 |
| 1419162_s_at | Cry2 | 0.60480756 |
| 1437345_a_at | Tfpt | 0.60515743 |
| 1424646_at | Gla | 0.6051651 |
| 1450377_at | Lyrm2 | 0.6053362 |
| 1449686_s_at | Atp8b4 | 0.60568666 |
| 1449168_a_at | E2f6 | 0.6058426 |
| 1455204_at | Parp3 | 0.60587925 |
| 1424829_at | AW551984 | 0.60595524 |
| 1428695_at | 4930402H24Rik | 0.6061132 |
| 1417278_a_at | --- | 0.60614973 |
| 1428199_at | Rilpl2 | 0.60618216 |
| 1439064_at | Cpeb3 | 0.6061871 |
| 1438644_x_at | Tmco4 | 0.6062121 |
| 1417848_at | H13 | 0.60630476 |
| 1453282_at | Commd9 | 0.60643893 |
| 1417148_at | Map1lc3a | 0.6065919 |
| 1441709_at | Hsd17b7 | 0.60672134 |
| 1435345_at | LOC100044968 | 0.6067857 |
| 1418445_at | Maff | 0.60683864 |
| 1456945_at | Map3k14 | 0.6070813 |
| 1439840_at | 1110003E01Rik | 0.6070975 |
| 1453212_at | Skap2 | 0.607118 |
| 1416983_s_at | Copz2 | 0.6075395 |
| 1436401_at | --- | 0.60757935 |
| 1438963_s_at | 6330416G13Rik | 0.6077688 |
| 1453418_at | Pole4 | 0.6079562 |
| 1418955_at | Adcy9 | 0.60810125 |
| 1418535_at | Golgb1 | 0.6084786 |
| 1452148_at | --- | 0.6089907 |
| 1449621_s_at | Cat | 0.6091554 |
| 1439191_at | Camkk2 | 0.6094317 |
| 1420696_at | 0610007P14Rik | 0.609447 |
| 1437766_at | 2310033K02Rik | 0.6095485 |
| 1423572_at | Hspa12a | 0.6099887 |
| 1425558_at | --- | 0.610131 |
| 1436791_at | Stat3 | 0.61030245 |
| 1440691_at | Farp2 | 0.610311 |
| 1418760_at | Lrpap1 | 0.6103311 |
| 1460412_at | Dennd1a | 0.61034924 |
| 1449372_at | Cdc42ep4 | 0.6105073 |
| 1428821_at | Meis2 | 0.6105093 |
| 1425458_a_at | Surf4 | 0.6105276 |
| 1421061_at | C330011K17Rik | 0.6106707 |
| 1434425_at | Ikbke | 0.61067265 |
| 1418626_a_at | Zfp260 | 0.6107554 |
| 1436970_a_at | Bace2 | 0.61106896 |
| 1417104_at | 2310009A05Rik | 0.6111694 |
| 1417107_at | Fgfr1 | 0.61129194 |
| 1436033_at | Gp1bb | 0.61137253 |
| 1455395_at | Pkd2 | 0.6115423 |
| 1455898_x_at | E130016E03Rik | 0.6120819 |
| 1434404_at | Slc38a9 | 0.6122189 |
| 1448557_at | Snhg10 | 0.6123036 |
| 1433769_at | Slc25a45 | 0.61232543 |
| 1436502_at | Dfna5h | 0.6124735 |
| 1422917_at | Dnajc1 | 0.6125009 |
| 1447862_x_at | Zfyve21 | 0.61266863 |
| 1444008_at | Adcy9 | 0.6126764 |
| 1448666_s_at | Ptpdc1 | 0.6128157 |
| 1453266_at | Wbscr27 | 0.61293787 |
| 1452843_at | Lgr4 | 0.61305195 |
| 1424359_at | Lyst | 0.6135523 |
| 1423379_at | 2310028H24Rik | 0.6138667 |
| 1454974_at | Hbp1 | 0.6139741 |
| 1427391_a_at | Pxk | 0.6142526 |
| 1439687_at | 9030419F21Rik | 0.6142942 |
| 1460049_s_at | Hist2h2be | 0.614337 |
| 1417393_a_at | --- | 0.6143878 |
| 1419191_at | Fuca1 | 0.61461854 |
| 1440304_at | Klhl5 | 0.6146963 |
| 1460623_at | Centd2 | 0.6147076 |
| 1457230_at | Dcun1d3 | 0.6151836 |
| 1447883_x_at | OTTMUSG00000016644 | 0.61518466 |
| 1421976_at | Trafd1 | 0.61533356 |
| 1443757_x_at | Adc | 0.6160147 |
| 1415856_at | Shox2 | 0.6160388 |
| 1422466_at | --- | 0.61612535 |
| 1428026_at | Pak3 | 0.6162282 |
| 1442406_at | Slc4a2 | 0.61644745 |
| 1425526_a_at | 2700079J08Rik | 0.6168276 |
| 1423230_at | Vamp4 | 0.6171282 |
| 1435606_at | 5330426P16Rik | 0.6177126 |
| 1439790_at | Slc38a2 | 0.617758 |
| 1435158_at | Cd99 | 0.6178427 |
| 1417460_at | Ldhb | 0.6180473 |
| 1439764_s_at | Meis2 | 0.6181114 |
| 1434128_a_at | Peg10 | 0.6183836 |
| 1416578_at | Ick | 0.61856323 |
| 1440660_at | Ptpdc1 | 0.61884755 |
| 1455870_at | Lman2 | 0.6188574 |
| 1446850_at | Nedd9 | 0.6188729 |
| 1445316_at | 8430420F16Rik | 0.6188738 |
| 1451235_at | Rcn3 | 0.61935043 |
| 1456720_at | Dnajc3a | 0.61968863 |
| 1418749_at | 1300018J18Rik | 0.6199683 |
| 1426292_at | Jarid1b | 0.6200881 |
| 1448871_at | 4632428N05Rik | 0.6200978 |
| 1435872_at | Dnajc3a | 0.62018937 |
| 1429399_at | 3110001I20Rik | 0.6202985 |
| 1426799_at | Naaa | 0.6204715 |
| 1431208_a_at | Casp12 | 0.62055475 |
| 1458746_at | EG432879 | 0.62067306 |
| 1444089_at | Pkd2 | 0.6217479 |
| 1419416_a_at | Sidt2 | 0.6218242 |
| 1422821_s_at | Ccl27 | 0.62203854 |
| 1428579_at | Slc38a9 | 0.62244755 |
| 1426894_s_at | Dnase2a | 0.622648 |
| 1440038_at | Angptl2 | 0.62318724 |
| 1435993_at | 2310068J16Rik | 0.6232655 |
| 1436697_at | Rab9 | 0.6235094 |
| 1449195_s_at | --- | 0.62357986 |
| 1449001_at | Slc9a3r2 | 0.623844 |
| 1416926_at | Cdkn2b | 0.6239718 |
| 1440847_at | Nkd1 | 0.6241211 |
| 1435743_at | 2310009A05Rik | 0.6243046 |
| 1421075_s_at | Utp14b | 0.6245028 |
| 1427919_at | Numbl | 0.6246806 |
| 1448875_at | Myst1 | 0.6247887 |
| 1418980_a_at | Nedd9 | 0.6248881 |
| 1449528_at | Tsnax | 0.62501425 |
| 1416675_s_at | Ap3m2 | 0.6250615 |
| 1436294_at | Slc39a13 | 0.62522185 |
| 1426298_at | Sptlc2 | 0.6254295 |
| 1427592_at | Gpr176 | 0.62553066 |
| 1427516_a_at | Usp25 | 0.62578243 |
| 1447602_x_at | Klhl5 | 0.6258197 |
| 1453242_x_at | Tob2 | 0.625973 |
| 1425515_at | Sdc1 | 0.62614465 |
| 1453255_at | AI504432 | 0.6264399 |
| 1444531_at | Psmd9 | 0.62656194 |
| 1448665_at | Urm1 | 0.6267528 |
| 1437667_a_at | A930008G19Rik | 0.62682176 |
| 1451416_a_at | Ppfibp2 | 0.6270273 |
| 1429722_at | Crlf2 | 0.6271567 |
| 1419721_at | Gsk3b | 0.6272278 |
| 1435785_at | Cmtm3 | 0.6273615 |
| 1437927_at | Lman2 | 0.62747884 |
| 1425037_at | 1500011B03Rik | 0.6276655 |
| 1424634_at | Gcc1 | 0.6279142 |
| 1443939_at | Dbt | 0.6282675 |
| 1425840_a_at | Thg1l | 0.6283974 |
| 1436989_s_at | Mrps12 | 0.6286998 |
| 1421066_at | Pold4 | 0.6287434 |
| 1435097_at | Ltb4dh | 0.628814 |
| 1438123_at | Ttc17 | 0.6291139 |
| 1425913_a_at | Cdc14b | 0.6291329 |
| 1454753_at | Ptprj | 0.62914175 |
| 1424807_at | Stat5a | 0.6292734 |
| 1424183_at | BC022687 | 0.62927955 |
| 1447345_at | Gpr109a | 0.629386 |
| 1427019_at | --- | 0.62942743 |
| 1439345_at | Prrx1 | 0.62949055 |
| 1431259_at | Sdc1 | 0.6297816 |
| 1422177_at | 9930031P18Rik | 0.63012207 |
| 1447096_at | Ehd2 | 0.6304256 |
| 1449298_a_at | Adam19 | 0.6305132 |
| 1454283_at | Commd9 | 0.63065755 |
| 1423596_at | Mpv17 | 0.63073534 |
| 1443270_at | --- | 0.63103646 |
| 1430514_a_at | C330006P03Rik | 0.63115895 |
| 1417849_at | Zscan18 | 0.6312414 |
| 1429693_at | Ppm1a | 0.63163817 |
| 1423422_at | Zfp93 | 0.6319611 |
| 1421612_a_at | D1Ertd471e | 0.6320426 |
| 1444073_at | Lrig1 | 0.6321092 |
| 1425060_s_at | Zfp383 | 0.6325559 |
| 1424268_at | C1qdc2 | 0.6326407 |
| 1460243_at | Gats | 0.6326786 |
| 1453055_at | 2210419D22Rik | 0.6326804 |
| 1431619_a_at | Rai14 | 0.6327219 |
| 1449334_at | Eif2c4 | 0.633083 |
| 1417574_at | AW987390 | 0.63325083 |
| 1428198_at | Cldnd1 | 0.63331616 |
| 1434361_at | Rilpl1 | 0.63371 |
| 1448162_at | Stat3 | 0.63389945 |
| 1451775_s_at | AA986860 | 0.6343778 |
| 1439364_a_at | BC043301 | 0.6344288 |
| 1417129_a_at | Dnajc3a | 0.6346188 |
| 1419043_a_at | Angptl2 | 0.6349253 |
| 1431932_s_at | C730025P13Rik | 0.63511765 |
| 1434820_s_at | Dusp22 | 0.6358174 |
| 1429265_a_at | Ssh1 | 0.63588554 |
| 1446512_at | Masp1 | 0.63666 |
| 1415874_at | Acat1 | 0.6366681 |
| 1426221_at | Atg4b | 0.6368719 |
| 1420760_s_at | Tnks1bp1 | 0.6373926 |
| 1440238_at | --- | 0.63755876 |
| 1449303_at | Rnf11 | 0.63763565 |
| 1417757_at | Isoc2b | 0.63766974 |
| 1434411_at | Zfp707 | 0.63777447 |
| 1417627_a_at | Fstl1 | 0.63796157 |
| 1426231_at | Nfe2l2 | 0.63805014 |
| 1453514_at | Map1lc3a | 0.6383735 |
| 1451939_a_at | Pak3 | 0.6383781 |
| 1447944_at | Antxr1 | 0.63839304 |
| 1458802_at | Birc2 | 0.6384913 |
| 1459579_at | BC008163 | 0.63896805 |
| 1435727_s_at | Apbb1ip | 0.6390563 |
| 1422498_at | Zfp329 | 0.639081 |
| 1451257_at | Pvrl2 | 0.63912344 |
| 1425942_a_at | Nfkbia | 0.6391395 |
| 1436788_at | OTTMUSG00000008561 | 0.639205 |
| 1451271_a_at | 7-Mar | 0.6392383 |
| 1450085_at | Slc25a36 | 0.6394317 |
| 1454967_at | Ltbp3 | 0.6396843 |
| 1460667_at | Foxo1 | 0.6398381 |
| 1417179_at | Ablim1 | 0.63993675 |
| 1426587_a_at | 8430436O14Rik | 0.64006263 |
| 1445539_at | Timp2 | 0.64045656 |
| 1435565_at | Sdc1 | 0.6404841 |
| 1454671_at | Mettl7a1 | 0.64075893 |
| 1438110_at | --- | 0.64089936 |
| 1433662_s_at | Serp2 | 0.64095116 |
| 1448117_at | Tacc1 | 0.64110565 |
| 1432524_at | Cdadc1 | 0.6413807 |
| 1434028_at | Zfp790 | 0.6416123 |
| 1433449_at | --- | 0.6417419 |
| 1427358_a_at | Usp12 | 0.6420431 |
| 1443770_x_at | Slc11a2 | 0.64204836 |
| 1434458_at | Foxo1 | 0.6427141 |
| 1417101_at | --- | 0.64279574 |
| 1460197_a_at | Wdr70 | 0.6428567 |
| 1434279_at | Spnb2 | 0.6428672 |
| 1425850_a_at | Zbtb43 | 0.64328057 |
| 1441842_s_at | Snapc3 | 0.64335316 |
| 1435893_at | Zfp606 | 0.6434644 |
| 1455965_at | Camkk2 | 0.64358485 |
| 1429514_at | Clip4 | 0.6441337 |
| 1426260_a_at | Uaca | 0.6442065 |
| 1452744_at | 4931408A02Rik | 0.6444609 |
| 1417152_at | Cat | 0.64454347 |
| 1416301_a_at | Tbc1d13 | 0.64454955 |
| 1417852_x_at | Hspa2 | 0.64458793 |
| 1455796_x_at | 5430404G13Rik | 0.6445928 |
| 1429033_at | Acss2 | 0.6446284 |
| 1449130_at | 6820431F20Rik | 0.64475286 |
| 1455860_at | 4921505C17Rik | 0.644823 |
| 1436325_at | 9630023C09Rik | 0.6448249 |
| 1416302_at | Auts2 | 0.6449384 |
| 1418718_at | Hipk3 | 0.6452009 |
| 1440935_at | 1810048J11Rik | 0.6452565 |
| 1422977_at | A930006K02Rik | 0.6455565 |
| 1439573_at | Vamp4 | 0.64581597 |
| 1433725_at | Cerk | 0.6459681 |
| 1429896_at | Spred1 | 0.6460349 |
| 1423819_s_at | Spred1 | 0.6461851 |
| 1447300_at | Rbms2 | 0.6462955 |
| 1421818_at | Mvk | 0.6463071 |
| 1421087_at | Cd47 | 0.6466144 |
| 1431352_s_at | Loxl3 | 0.64677 |
| 1434278_at | Nfia | 0.64687985 |
| 1426208_x_at | Dirc2 | 0.6469295 |
| 1444628_at | Il1r2 | 0.64706373 |
| 1459868_x_at | Bet1l | 0.64711016 |
| 1439123_at | Snx13 | 0.64730304 |
| 1422615_at | Otud5 | 0.64739615 |
| 1418013_at | Tm9sf2 | 0.6474897 |
| 1428331_at | Tmem50b | 0.6475882 |
| 1448865_at | D530033C11Rik | 0.64760226 |
| 1451264_at | Cend1 | 0.64763296 |
| 1416741_at | Pdpn | 0.6479036 |
| 1453268_at | Chmp2b | 0.64903224 |
| 1416783_at | Acp2 | 0.6492136 |
| 1452840_at | Tbc1d24 | 0.6492462 |
| 1434330_at | Stx12 | 0.649299 |
| 1416101_a_at | Rab14 | 0.6493649 |
| 1423134_at | Nkapl | 0.6495157 |
| 1420904_at | Oraov1 | 0.6495346 |
| 1434499_a_at | Tpd52l2 | 0.6498705 |
| 1435120_at | Mycbp2 | 0.64992714 |
| 1457042_at | Ddr2 | 0.65010494 |
| 1431995_at | Nrbp2 | 0.6501406 |
| 1455396_at | Acvr1 | 0.6501444 |
| 1426883_at | Mxd1 | 0.6502172 |
| 1423611_at | Zc3h15 | 0.65022403 |
| 1434129_s_at | Mark4 | 0.65035385 |
| 1429987_at | Kdelr3 | 0.6503724 |
| 1424608_a_at | Per3 | 0.65041894 |
| 1438800_at | Scp2 | 0.6506644 |
| 1459595_at | Htatip2 | 0.65101236 |
| 1436459_at | Arhgap17 | 0.65124273 |
| 1416358_at | Myo9a | 0.6513189 |
| 1453243_at | 5230400M03Rik | 0.6515538 |
| 1444041_at | Prkg1 | 0.65164447 |
| 1418945_at | Phf2 | 0.6518301 |
| 1416109_at | Atp2a2 | 0.6520788 |
| 1455235_x_at | --- | 0.6521914 |
| 1430388_a_at | --- | 0.6522518 |
| 1437718_x_at | Dhrs7 | 0.6524045 |
| 1439549_at | Prkd2 | 0.6524916 |
| 1435830_a_at | Aak1 | 0.6525568 |
| 1453924_a_at | Dtnbp1 | 0.6525901 |
| 1440037_at | Fmnl2 | 0.6526226 |
| 1448908_at | 4930550G17Rik | 0.6527009 |
| 1431820_at | --- | 0.65293366 |
| 1435238_x_at | Bzw2 | 0.6529384 |
| 1449494_at | Elavl4 | 0.6533262 |
| 1454654_at | Arfgap3 | 0.65378016 |
| 1439255_s_at | Tollip | 0.653856 |
| 1429444_at | 11-Sep | 0.65389514 |
| 1437748_at | Hdlbp | 0.653916 |
| 1422094_a_at | 1110019K23Rik | 0.65443027 |
| 1444433_at | Cnp | 0.65446115 |
| 1455971_at | Lima1 | 0.6544629 |
| 1439136_at | Maf | 0.65455127 |
| 1454838_s_at | Wipi2 | 0.6545973 |
| 1452911_at | Tank | 0.6546687 |
| 1436438_s_at | --- | 0.65484774 |
| 1439806_at | --- | 0.6549209 |
| 1457139_at | Zfp574 | 0.65495455 |
| 1438171_x_at | Higd1a | 0.6553567 |
| 1456084_x_at | Itm2c | 0.65547436 |
| 1424923_at | Pla2g4a | 0.6558872 |
| 1457034_at | Papola | 0.6559048 |
| 1439231_at | Zkscan5 | 0.6561226 |
| 1458657_at | Nek9 | 0.65633136 |
| 1418129_at | Cd82 | 0.656556 |
| 1424034_at | Tspan5 | 0.65680635 |
| 1423687_a_at | A930008G19Rik | 0.6569236 |
| 1438520_at | Nrip1 | 0.65707904 |
| 1433501_at | --- | 0.6571746 |
| 1452632_at | Osbpl6 | 0.6573619 |
| 1422478_a_at | --- | 0.65749055 |
| 1445564_at | Pogk | 0.65754086 |
| 1421228_at | Zfp704 | 0.65779483 |
| 1452318_a_at | Adnp | 0.65787286 |
| 1449450_at | Mettl9 | 0.6580107 |
| 1449533_at | Caprin1 | 0.6580452 |
| 1451309_at | --- | 0.65804756 |
| 1419309_at | C1qtnf5 | 0.658092 |
| 1421199_at | Psmd8 | 0.65815455 |
| 1426819_at | Rbx1 | 0.65822124 |
| 1440954_at | Ppm1k | 0.6585365 |
| 1435714_x_at | Cd99 | 0.65855324 |
| 1450699_at | Inpp5e | 0.6585674 |
| 1439090_at | Bcl2l2 | 0.65879506 |
| 1425663_at | Zfp84 | 0.65884197 |
| 1448775_at | 4833408D11Rik | 0.6591238 |
| 1438855_x_at | 1200007C13Rik | 0.65916646 |
| 1455607_at | --- | 0.6592111 |
| 1427127_x_at | Ank3 | 0.65943307 |
| 1452366_at | 2610110G12Rik | 0.6594389 |
| 1446086_s_at | Zfyve21 | 0.6595631 |
| 1449893_a_at | Tle3 | 0.65959483 |
| 1450449_a_at | 9630025I21Rik | 0.65972906 |
| 1416122_at | Mycbp2 | 0.6598718 |
| 1438682_at | Wdr22 | 0.6599388 |
| 1426405_at | Fbxo33 | 0.66011417 |
| 1451814_a_at | Bet1l | 0.66028154 |
| 1438685_at | --- | 0.6604804 |
| 1435259_s_at | Slc44a1 | 0.6605816 |
| 1437760_at | Acp2 | 0.6606954 |
| 1424497_at | Ccdc91 | 0.6607151 |
| 1452519_a_at | Spnb2 | 0.66082895 |
| 1418981_at | --- | 0.6611051 |
| 1434465_x_at | --- | 0.6612148 |
| 1447831_s_at | Pvt1 | 0.66136867 |
| 1434222_at | Ext2 | 0.66140676 |
| 1452063_at | Dpp8 | 0.661531 |
| 1424182_at | Rdh11 | 0.6617034 |
| 1421844_at | Timp2 | 0.6618686 |
| 1416619_at | Ivd | 0.6619999 |
| 1428858_at | Trio | 0.66219133 |
| 1446732_at | Gnpda2 | 0.6622203 |
| 1444302_at | Trio | 0.6623507 |
| 1417599_at | Shb | 0.66242075 |
| 1434427_a_at | Clip1 | 0.662482 |
| 1416948_at | --- | 0.6627855 |
| 1423141_at | Dok5 | 0.6628434 |
| 1436964_at | Rc3h2 | 0.6628863 |
| 1435354_at | Rreb1 | 0.66306686 |
| 1416481_s_at | Ift20 | 0.6631645 |
| 1420349_at | 9230104K21Rik | 0.6635088 |
| 1420688_a_at | Riok3 | 0.66372335 |
| 1421074_at | --- | 0.66393334 |
| 1424050_s_at | 1110032O16Rik | 0.6640434 |
| 1417960_at | Hs6st1 | 0.6641013 |
| 1419165_at | Uckl1 | 0.66410965 |
| 1434674_at | Kdelc2 | 0.6642572 |
| 1450213_at | Stk3 | 0.66433674 |
| 1433505_a_at | 4921537I17Rik | 0.66439503 |
| 1416511_a_at | Rnf24 | 0.66440254 |
| 1436456_at | Pcdh7 | 0.66442156 |
| 1418100_at | Dab2ip | 0.66466546 |
| 1439610_at | Aff1 | 0.6647608 |
| 1418941_at | C79130 | 0.664979 |
| 1428657_at | Arid5b | 0.66509515 |
| 1428851_at | Plekhm1 | 0.6653385 |
| 1427820_at | Dennd2a | 0.6653946 |
| 1426784_at | Insig1 | 0.6655228 |
| 1420443_at | Gramd1a | 0.66562754 |
| 1424068_at | Ubfd1 | 0.6656547 |
| 1426246_at | Prpf6 | 0.6658896 |
| 1456160_at | Cnih4 | 0.6660256 |
| 1428894_at | Cav1 | 0.6661962 |
| 1420715_a_at | Ttf1 | 0.6662221 |
| 1457248_x_at | 0610030E20Rik | 0.66626155 |
| 1451479_a_at | Tpbg | 0.66642964 |
| 1420905_at | Nfkbiz | 0.6664388 |
| 1450872_s_at | Zfp260 | 0.66645616 |
| 1425537_at | Insig2 | 0.66649604 |
| 1419712_at | Kptn | 0.66650313 |
| 1419435_at | --- | 0.6665684 |
| 1460367_at | Pcca | 0.6665818 |
| 1431704_a_at | 2810002D19Rik | 0.66665554 |
| 1437467_at | Rabggtb | 1.5004561 |
| 1453361_at | 9530058B02Rik | 1.500472 |
| 1436400_at | Twsg1 | 1.5008405 |
| 1456591_x_at | Ccdc86 | 1.5013478 |
| 1428960_at | Ptprd | 1.5013553 |
| 1419315_at | Syk | 1.5014949 |
| 1419598_at | Reep6 | 1.5015001 |
| 1417914_at | LOC100047915 | 1.5019457 |
| 1420343_at | Plaur | 1.5020349 |
| 1421052_a_at | Med14 | 1.5020819 |
| 1428534_at | Itgb1 | 1.5022235 |
| 1437244_at | Cpne4 | 1.5022808 |
| 1423477_at | Tmpo | 1.5027561 |
| 1455405_at | Saal1 | 1.5028654 |
| 1417031_at | Kbtbd8 | 1.5029638 |
| 1448740_at | Cenpq | 1.5032668 |
| 1433266_at | Rell2 | 1.5038077 |
| 1442018_at | Dhps | 1.503915 |
| 1419582_at | Gjb5 | 1.5040004 |
| 1434917_at | Emp1 | 1.5044305 |
| 1421190_at | Cdca7l | 1.5054228 |
| 1418777_at | C920008N22Rik | 1.5054752 |
| 1428291_at | Pa2g4 | 1.5056448 |
| 1427735_a_at | Cdc5l | 1.5057414 |
| 1426239_s_at | Sfrs6 | 1.5058553 |
| 1429870_at | Mllt11 | 1.506174 |
| 1458249_at | Spink10 | 1.5063558 |
| 1423120_at | Ide | 1.506358 |
| 1423569_at | Iqgap3 | 1.5064538 |
| 1418540_a_at | Zfp617 | 1.5064585 |
| 1417910_at | Suclg2 | 1.5065383 |
| 1444480_at | Tmtc4 | 1.5065992 |
| 1460468_s_at | Zfpm2 | 1.5067798 |
| 1426570_a_at | Pabpc4l | 1.5068506 |
| 1425534_at | Pde7a | 1.5071647 |
| 1449125_at | Ccna2 | 1.5071906 |
| 1452031_at | D10627 | 1.5073926 |
| 1458165_at | Slc5a3 | 1.5075153 |
| 1453069_at | --- | 1.5075825 |
| 1446897_at | 9530004M16Rik | 1.507701 |
| 1426472_at | --- | 1.5077477 |
| 1425118_at | Rfxank | 1.507949 |
| 1422011_s_at | BC057552 | 1.508492 |
| 1449965_at | Rnf217 | 1.5087174 |
| 1419619_at | Mapk6 | 1.508751 |
| 1416382_at | Ttc13 | 1.5088836 |
| 1441666_at | Emp2 | 1.5090067 |
| 1449865_at | Phca | 1.5090868 |
| 1428523_at | Trmt1 | 1.5093445 |
| 1452592_at | Gcn5l2 | 1.5096526 |
| 1456307_s_at | Cep55 | 1.5098767 |
| 1426423_at | Sos1 | 1.5099587 |
| 1429463_at | Jph1 | 1.5103898 |
| 1449936_at | Chd1 | 1.5107028 |
| 1456126_at | Ube2c | 1.5107459 |
| 1424143_a_at | Wdr43 | 1.5110838 |
| 1446421_at | Ppap2c | 1.5111914 |
| 1449928_at | --- | 1.5114808 |
| 1428963_at | Dcun1d1 | 1.5116066 |
| 1438272_at | 4931407J08Rik | 1.5121585 |
| 1456652_at | Narg1l | 1.5125597 |
| 1449885_at | Fxr1 | 1.5127649 |
| 1452080_a_at | Ttc27 | 1.5130798 |
| 1426994_at | Eny2 | 1.513198 |
| 1438210_at | Col4a6 | 1.5137309 |
| 1429703_at | D17Wsu104e | 1.514069 |
| 1419717_at | --- | 1.5145062 |
| 1444319_at | Lrrc1 | 1.5145243 |
| 1426605_at | Slc30a4 | 1.5145357 |
| 1423292_a_at | Kpnb1 | 1.5152696 |
| 1448914_a_at | Mcm5 | 1.5155013 |
| 1452598_at | Polr3c | 1.5155839 |
| 1422704_at | --- | 1.5161157 |
| 1456386_at | Igf2bp1 | 1.5164282 |
| 1453139_at | Hoxc4 | 1.5166533 |
| 1457167_at | Lama5 | 1.5168011 |
| 1432059_x_at | Ttc14 | 1.5175117 |
| 1425155_x_at | Cenpn | 1.5175816 |
| 1427819_at | AK220484 | 1.5180498 |
| 1438701_at | 4930506M07Rik | 1.5182283 |
| 1439078_at | --- | 1.5183563 |
| 1425059_at | --- | 1.519015 |
| 1434348_at | 0610010F05Rik | 1.5192066 |
| 1446809_at | Grwd1 | 1.5193905 |
| 1438325_at | Alg13 | 1.5194503 |
| 1439347_at | Plscr2 | 1.5195804 |
| 1428696_at | Wdr46 | 1.5195968 |
| 1431255_at | Xrn2 | 1.5197097 |
| 1450607_s_at | Papd5 | 1.5199066 |
| 1422038_a_at | Gtsf1 | 1.5203615 |
| 1434653_at | Slc18a2 | 1.5203924 |
| 1449645_s_at | D17Wsu104e | 1.5206478 |
| 1418152_at | LOC100048559 | 1.5209627 |
| 1421566_at | Cmbl | 1.5210222 |
| 1415793_at | LOC100047915 | 1.5214448 |
| 1424955_at | Tro | 1.5215894 |
| 1434287_at | Snx16 | 1.5217476 |
| 1417040_a_at | Acd | 1.5218707 |
| 1444254_at | Prkar2b | 1.521906 |
| 1426929_at | Anxa4 | 1.5220542 |
| 1442880_at | Nol11 | 1.5220866 |
| 1424833_at | Trim37 | 1.5221082 |
| 1437152_at | AA409261 | 1.5226852 |
| 1456684_at | Arv1 | 1.5228846 |
| 1458389_at | D17Ertd663e | 1.5230895 |
| 1443978_at | --- | 1.5231357 |
| 1416953_at | Sema3g | 1.523523 |
| 1451431_a_at | Ulbp1 | 1.5237737 |
| 1424567_at | 1810029B16Rik | 1.5240729 |
| 1419494_a_at | Rfxap | 1.5241796 |
| 1422982_at | Mum1l1 | 1.524601 |
| 1453972_x_at | Tmem40 | 1.5248007 |
| 1452171_at | Ccl25 | 1.5250694 |
| 1419697_at | Isoc1 | 1.5253901 |
| 1424704_at | Uhrf1 | 1.5261661 |
| 1418638_at | Zfp364 | 1.5261666 |
| 1417146_at | Ahcyl1 | 1.5268366 |
| 1439807_at | Nkrf | 1.5272278 |
| 1436155_at | --- | 1.5272319 |
| 1416554_at | Nes | 1.5272417 |
| 1424176_a_at | Gas2l3 | 1.5275509 |
| 1448439_at | Pa2g4 | 1.5275967 |
| 1453761_at | Sgsm1 | 1.5277379 |
| 1449821_a_at | Stk10 | 1.5277449 |
| 1441917_s_at | 3200002M19Rik | 1.5278213 |
| 1435308_at | Bxdc5 | 1.5280993 |
| 1428452_at | Znrf2 | 1.5286661 |
| 1460238_at | Stxbp5 | 1.5290794 |
| 1418219_at | 1700020C11Rik | 1.5291561 |
| 1441994_at | Rbm39 | 1.5297385 |
| 1459731_at | Rap2b | 1.5301646 |
| 1418046_at | Dclk1 | 1.5308007 |
| 1425790_a_at | Cyp39a1 | 1.531513 |
| 1450854_at | Pla2g12a | 1.531669 |
| 1441340_at | Pnpla6 | 1.5318806 |
| 1424792_at | Hnrnpd | 1.5324789 |
| 1450496_a_at | Bbs12 | 1.5328734 |
| 1434239_at | rp9 | 1.5329151 |
| 1429723_at | --- | 1.5331106 |
| 1424568_at | LOC100046214 | 1.5334355 |
| 1437548_at | Vac14 | 1.5337962 |
| 1416313_at | D0Kist2 | 1.5342875 |
| 1458375_at | Cacnb2 | 1.5345224 |
| 1426073_at | 2810433K01Rik | 1.5348592 |
| 1436222_at | Arhgef2 | 1.5348746 |
| 1418602_at | 4833424O12Rik | 1.5349234 |
| 1435383_x_at | 2810474O19Rik | 1.5351831 |
| 1415803_at | Dhrs13 | 1.5358143 |
| 1453233_s_at | Rnpc3 | 1.5358822 |
| 1451454_at | Ahcyl2 | 1.5361383 |
| 1422671_s_at | Narg1 | 1.5366167 |
| 1433521_at | Ankrd13b | 1.5369654 |
| 1445919_at | Nfya | 1.537246 |
| 1430667_at | Rad23a | 1.5378275 |
| 1436329_at | Gemin5 | 1.5379087 |
| 1436361_at | Malt1 | 1.5385451 |
| 1451809_s_at | Arrb2 | 1.5386142 |
| 1424907_a_at | Bcl2 | 1.5387807 |
| 1420859_at | 4930430F08Rik | 1.539433 |
| 1460305_at | Eya4 | 1.5397408 |
| 1453988_a_at | Runx2 | 1.5407718 |
| 1455649_at | Nme6 | 1.5407947 |
| 1455790_at | Dynlt3 | 1.5409104 |
| 1442511_at | Hist1h4a | 1.5411742 |
| 1446899_at | Nr1d2 | 1.541229 |
| 1416559_at | Samd12 | 1.5417223 |
| 1447888_x_at | Jak3 | 1.5417544 |
| 1453468_at | Rwdd2a | 1.5423155 |
| 1436103_at | Pde7a | 1.542545 |
| 1457568_at | 1810005K13Rik | 1.5425732 |
| 1438051_at | Gins3 | 1.5426159 |
| 1419289_a_at | Col4a5 | 1.5428848 |
| 1454750_a_at | Nup43 | 1.5429064 |
| 1437064_at | Ctbs | 1.5432714 |
| 1427287_s_at | Kcnmb1 | 1.5436224 |
| 1426544_a_at | Arl6ip2 | 1.5437635 |
| 1424793_a_at | Ccr1 | 1.5439075 |
| 1417911_at | Wasf1 | 1.5439091 |
| 1428670_at | Phf17 | 1.5439608 |
| 1452232_at | --- | 1.544317 |
| 1432187_at | Farsa | 1.5443313 |
| 1434067_at | Tom1l1 | 1.5445751 |
| 1425051_at | Sacs | 1.5446417 |
| 1447870_x_at | Ercc6l | 1.5446681 |
| 1460617_s_at | Kif11 | 1.5452812 |
| 1436737_a_at | Mrpl47 | 1.5455577 |
| 1421038_a_at | 6430706D22Rik | 1.5457087 |
| 1421727_at | Rps15a | 1.5458286 |
| 1452635_x_at | Fxr1 | 1.545941 |
| 1430606_at | E2f5 | 1.5459588 |
| 1427434_at | Sh2d1b1 | 1.54631 |
| 1439152_at | Zcchc18 | 1.5463151 |
| 1440602_at | Frk | 1.5463303 |
| 1426526_s_at | Epb4.1l5 | 1.546333 |
| 1425463_at | Rtn4 | 1.5479778 |
| 1455861_at | Crmp1 | 1.5479894 |
| 1437195_x_at | 6330409N04Rik | 1.5480893 |
| 1447980_s_at | Suclg2 | 1.549275 |
| 1453550_a_at | Suz12 | 1.549351 |
| 1452075_at | Wee1 | 1.5497004 |
| 1456401_at | Taf4b | 1.5498524 |
| 1459747_at | Tinagl | 1.5500047 |
| 1427518_at | Lingo2 | 1.5501033 |
| 1423915_at | Leprel1 | 1.5502534 |
| 1438324_at | 5730590G19Rik | 1.5502809 |
| 1420858_at | Acy3 | 1.5503061 |
| 1448289_at | Ccdc41 | 1.5508122 |
| 1433670_at | Cdt1 | 1.5513461 |
| 1436186_at | Fhit | 1.5515072 |
| 1416915_at | Rasa4 | 1.5515321 |
| 1449546_a_at | Rap2b | 1.5517273 |
| 1418412_at | Mgat4a | 1.551919 |
| 1418726_a_at | --- | 1.5519772 |
| 1422562_at | Adat2 | 1.5519955 |
| 1434194_at | Mef2c | 1.5520605 |
| 1451152_a_at | Pik3c2a | 1.5520947 |
| 1455817_x_at | LOC100042253 | 1.552183 |
| 1457089_at | --- | 1.5526171 |
| 1417553_at | Ncam1 | 1.5527506 |
| 1423624_at | 1500003O22Rik | 1.5528567 |
| 1460314_s_at | --- | 1.5533055 |
| 1445882_at | Sgsm1 | 1.5534452 |
| 1426369_at | Ctr9 | 1.5537044 |
| 1452240_at | Ccdc5 | 1.5537964 |
| 1429372_at | Trmt11 | 1.5538024 |
| 1436465_at | Frk | 1.5538559 |
| 1454120_a_at | Itga3 | 1.5541018 |
| 1454197_a_at | Msh2 | 1.5543506 |
| 1425416_s_at | Lsm2 | 1.5544833 |
| 1429154_at | Pcgf6 | 1.5545111 |
| 1416231_at | Arsg | 1.5550191 |
| 1416646_at | Rrm1 | 1.5551059 |
| 1421997_s_at | Tfb1m | 1.5553517 |
| 1452807_s_at | Slc5a3 | 1.5553532 |
| 1423848_at | Mlstd2 | 1.5554876 |
| 1439307_at | Dusp6 | 1.555667 |
| 1423465_at | E2f5 | 1.5563115 |
| 1426609_at | Rap2b | 1.5564346 |
| 1458114_at | Trim37 | 1.5579524 |
| 1444980_at | Stmn2 | 1.558011 |
| 1447275_at | Dnajc19 | 1.5584595 |
| 1452114_s_at | Elovl4 | 1.5585567 |
| 1441325_at | Shmt2 | 1.558826 |
| 1426910_at | Pecr | 1.5591873 |
| 1429579_at | --- | 1.5594578 |
| 1421265_a_at | Sass6 | 1.5596399 |
| 1428390_at | Klf5 | 1.5601629 |
| 1421027_a_at | Pold3 | 1.56017 |
| 1456603_at | Troap | 1.5602523 |
| 1435671_at | --- | 1.5604451 |
| 1420592_a_at | Med1 | 1.5605788 |
| 1439899_at | 2810004N23Rik | 1.5606064 |
| 1452092_at | Agpat5 | 1.5607163 |
| 1416052_at | Dctd | 1.561058 |
| 1452191_at | Mta3 | 1.5611085 |
| 1434003_a_at | --- | 1.5611473 |
| 1419589_at | Exosc8 | 1.5611616 |
| 1430139_at | Gcat | 1.5612996 |
| 1422170_at | --- | 1.5616633 |
| 1438366_x_at | --- | 1.5618165 |
| 1439093_at | Plekhh3 | 1.5622104 |
| 1450940_at | Rftn1 | 1.5623593 |
| 1460440_at | Cobl | 1.5625172 |
| 1417061_at | 2610528E23Rik | 1.5629033 |
| 1448539_a_at | D15Wsu169e | 1.5645323 |
| 1425464_at | Zxdb | 1.5648056 |
| 1427276_at | Ppp1r12a | 1.5649441 |
| 1418289_at | Hist1h3a | 1.5652046 |
| 1457045_at | Plb1 | 1.565214 |
| 1419169_at | Blvra | 1.5652872 |
| 1442624_at | 2810025M15Rik | 1.5653368 |
| 1452050_at | Nucb2 | 1.5654335 |
| 1438916_x_at | Wwp2 | 1.5655493 |
| 1416871_at | Wwc1 | 1.5660348 |
| 1449022_at | Msn | 1.5663213 |
| 1420420_at | Smtn | 1.566492 |
| 1417824_at | --- | 1.566617 |
| 1436827_at | Arrb2 | 1.5667425 |
| 1429792_at | Fmn2 | 1.5672175 |
| 1418545_at | Ipo7 | 1.5678142 |
| 1418815_at | Neil3 | 1.5681432 |
| 1424144_at | Inha | 1.5685227 |
| 1443750_s_at | Col8a1 | 1.5688928 |
| 1419130_at | --- | 1.5690393 |
| 1417378_at | 2900072G11Rik | 1.5693386 |
| 1420991_at | Rrp9 | 1.5702256 |
| 1437107_at | Gna14 | 1.5706842 |
| 1426370_at | LOC630729 | 1.5714356 |
| 1448910_at | Tspan2 | 1.5714694 |
| 1460273_a_at | Smpdl3b | 1.5717099 |
| 1443870_at | Utp20 | 1.5717235 |
| 1425053_at | Suz12 | 1.5723859 |
| 1440699_at | Hddc2 | 1.5723891 |
| 1437853_x_at | Zfp52 | 1.5725209 |
| 1440451_at | BC021395 | 1.5728883 |
| 1450423_s_at | Asf1b | 1.5731105 |
| 1420682_at | --- | 1.5731245 |
| 1416998_at | Scoc | 1.5732331 |
| 1455686_at | Epb4.1l5 | 1.5736169 |
| 1438716_at | Itpr2 | 1.5736516 |
| 1424589_s_at | Stxbp5 | 1.5737134 |
| 1450627_at | Nr2c2ap | 1.5737929 |
| 1452863_at | 9130011J15Rik | 1.5742384 |
| 1436544_at | Kirrel3 | 1.5749247 |
| 1429663_at | Tmem8 | 1.5751485 |
| 1420417_at | Anp32e | 1.5757229 |
| 1442745_x_at | Pstpip2 | 1.5762656 |
| 1420664_s_at | Gabarapl2 | 1.5766143 |
| 1435945_a_at | Foxp4 | 1.5767214 |
| 1427357_at | Slc25a30 | 1.5767407 |
| 1420906_at | Uchl3 | 1.5769438 |
| 1429172_a_at | Arntl | 1.5770929 |
| 1457032_at | Sgol1 | 1.5775822 |
| 1455771_at | Pus10 | 1.5779538 |
| 1433653_at | Tnfaip8l1 | 1.5786757 |
| 1438631_x_at | Ar | 1.5787603 |
| 1458264_at | Ncapg | 1.579022 |
| 1424231_s_at | Ccnf | 1.5791552 |
| 1431353_at | Gas5 | 1.5800444 |
| 1418022_at | D730048J04Rik | 1.5802462 |
| 1439305_at | Aprt | 1.5804725 |
| 1420990_at | Nup43 | 1.5807041 |
| 1444140_at | BC049349 | 1.5811498 |
| 1441447_at | 1190003J15Rik | 1.5812911 |
| 1452841_at | Nudt12 | 1.5817068 |
| 1429520_a_at | Exoc6 | 1.5824409 |
| 1452606_at | --- | 1.5825601 |
| 1455991_at | Setdb1 | 1.5825912 |
| 1433845_x_at | Smarca5 | 1.5828726 |
| 1459573_at | --- | 1.5831711 |
| 1453366_at | LOC100048307 | 1.5832973 |
| 1418859_at | Foxp4 | 1.5837076 |
| 1415810_at | Tmco7 | 1.5848765 |
| 1421118_a_at | Syt1 | 1.5850831 |
| 1450522_a_at | Dusp9 | 1.585809 |
| 1437122_at | Fez2 | 1.586069 |
| 1416721_s_at | Nars2 | 1.5862399 |
| 1425862_a_at | Fmn2 | 1.5863719 |
| 1451987_at | Folr1 | 1.5864111 |
| 1455717_s_at | Llgl2 | 1.5872258 |
| 1450505_a_at | Katnal2 | 1.5873687 |
| 1423952_a_at | 9430081I23Rik | 1.5874428 |
| 1421758_at | 5133401H06Rik | 1.587444 |
| 1451261_s_at | Ms4a6d | 1.5876414 |
| 1443694_at | Atic | 1.5882219 |
| 1443983_at | Isoc1 | 1.5882794 |
| 1424843_a_at | Tnfaip2 | 1.5884867 |
| 1431920_a_at | Depdc1a | 1.5885499 |
| 1420416_at | Gpr56 | 1.5892861 |
| 1455144_s_at | 5730409N24Rik | 1.5897658 |
| 1421881_a_at | AI451617 | 1.5899537 |
| 1452954_at | AK122209 | 1.5899799 |
| 1451884_a_at | Tspan2 | 1.5906078 |
| 1427275_at | Cct3 | 1.591202 |
| 1450134_at | Hmgb3 | 1.5923077 |
| 1439862_at | Tead4 | 1.5931983 |
| 1420835_at | Anxa3 | 1.5941448 |
| 1435006_s_at | Tnfrsf22 | 1.5945727 |
| 1437110_at | C430042M11Rik | 1.5951252 |
| 1453045_at | 2610528E23Rik | 1.5952953 |
| 1418539_a_at | --- | 1.5953748 |
| 1419844_a_at | Arhgap22 | 1.5955038 |
| 1445020_at | LOC100043462 | 1.5960966 |
| 1415834_at | Prim1 | 1.5965974 |
| 1417785_at | Usp15 | 1.5966082 |
| 1431088_at | Dkc1 | 1.5971905 |
| 1421152_a_at | Speer3 | 1.597308 |
| 1422445_at | --- | 1.5973135 |
| 1431786_s_at | Klhl4 | 1.597575 |
| 1433486_at | 2810433K01Rik | 1.5975811 |
| 1453329_s_at | Dhx36 | 1.5977337 |
| 1452834_at | Sbf2 | 1.5979244 |
| 1429654_at | Areg | 1.5984465 |
| 1454043_a_at | Fxr1 | 1.5990573 |
| 1430579_at | Farsb | 1.5993563 |
| 1446212_at | 2610207I05Rik | 1.5994868 |
| 1446088_at | Slc25a24 | 1.5999694 |
| 1421227_at | 2310001A20Rik | 1.6000563 |
| 1428663_at | Elavl1 | 1.6002825 |
| 1436337_at | 4933434I06Rik | 1.6005719 |
| 1435484_at | Glt25d1 | 1.6007276 |
| 1457262_at | Klrg2 | 1.6008494 |
| 1455223_at | Sh3tc2 | 1.6008863 |
| 1433398_at | Exoc6 | 1.6026989 |
| 1448440_x_at | Dhx36 | 1.6029516 |
| 1427588_a_at | Taf4b | 1.6029854 |
| 1452180_at | --- | 1.6036618 |
| 1425162_at | Tera | 1.6044521 |
| 1452252_at | Msto1 | 1.604615 |
| 1425140_at | Ttc13 | 1.6050192 |
| 1419482_at | Heatr3 | 1.6051497 |
| 1429901_at | Zdhhc3 | 1.6052811 |
| 1451794_at | 9330182L06Rik | 1.605777 |
| 1417057_a_at | Tanc2 | 1.6058309 |
| 1450195_at | Phf6 | 1.6060888 |
| 1418453_a_at | Wwc1 | 1.6063635 |
| 1431430_s_at | Col4a6 | 1.6068183 |
| 1428250_at | Reps1 | 1.6072505 |
| 1457528_at | Exo1 | 1.607304 |
| 1451210_at | Vars | 1.607912 |
| 1419126_at | LOC100043462 | 1.6083571 |
| 1443086_at | Gemin6 | 1.6088037 |
| 1437893_at | Ubash3b | 1.60892 |
| 1435564_at | Frrs1 | 1.6089559 |
| 1434354_at | Chtf18 | 1.60951 |
| 1428195_at | Rpp40 | 1.610079 |
| 1450933_at | Narg1 | 1.6109611 |
| 1449151_at | Bxdc1 | 1.6111327 |
| 1451293_at | Hcn1 | 1.611234 |
| 1422728_at | Park2 | 1.6114565 |
| 1426533_at | St8sia4 | 1.6114669 |
| 1436790_a_at | Ms4a6d | 1.6119609 |
| 1423135_at | Frk | 1.6135212 |
| 1420394_s_at | Rbl1 | 1.6138486 |
| 1459838_s_at | Dph5 | 1.6141783 |
| 1452315_at | Naf1 | 1.6143348 |
| 1415784_at | LOC100045432 | 1.6145023 |
| 1452251_at | Prcp | 1.6160352 |
| 1422938_at | Gdpd1 | 1.6164105 |
| 1457683_at | Sgk2 | 1.6171789 |
| 1442363_at | Lactb2 | 1.6175871 |
| 1420908_at | Bxdc1 | 1.6175994 |
| 1457191_at | Esf1 | 1.6177455 |
| 1425563_s_at | Pds5a | 1.6178826 |
| 1434357_a_at | BC025546 | 1.6180326 |
| 1423024_at | Bysl | 1.6187905 |
| 1460271_at | Psrc1 | 1.6191418 |
| 1459171_at | Slc40a1 | 1.6197997 |
| 1425292_at | --- | 1.6198746 |
| 1426865_a_at | Gdap1 | 1.6200986 |
| 1426908_at | Nmt2 | 1.6215506 |
| 1420992_at | Anxa4 | 1.621569 |
| 1419012_at | Usp10 | 1.6237092 |
| 1447040_at | Ccnd1 | 1.6249025 |
| 1457776_at | Daam2 | 1.6251724 |
| 1428751_at | Pcdh17 | 1.625279 |
| 1447979_at | Pgf | 1.6253456 |
| 1429049_at | BC025546 | 1.6262181 |
| 1428580_at | Reps1 | 1.6262337 |
| 1423411_at | Mybbp1a | 1.6264111 |
| 1455792_x_at | Acsbg1 | 1.6264565 |
| 1434993_at | --- | 1.6268193 |
| 1454224_at | Dbndd2 | 1.6277616 |
| 1417376_a_at | Josd3 | 1.6283818 |
| 1454955_at | Procr | 1.6288383 |
| 1442226_at | Eif2a | 1.6294872 |
| 1440227_at | Msh2 | 1.6295031 |
| 1456041_at | Pbp2 | 1.6305307 |
| 1442289_at | Rpo1-2 | 1.6306596 |
| 1418023_at | Cgn | 1.6316396 |
| 1424397_at | Klra18 | 1.6318407 |
| 1456798_at | Afp | 1.6318939 |
| 1458588_at | Rangrf | 1.6320753 |
| 1431176_at | Vgll2 | 1.6330612 |
| 1428834_at | 0610010F05Rik | 1.6331811 |
| 1423890_x_at | Trib3 | 1.6332414 |
| 1433844_a_at | Abcb7 | 1.6336201 |
| 1434252_at | Ucn2 | 1.6345447 |
| 1445345_at | Anp32e | 1.6348083 |
| 1418024_at | Isoc1 | 1.6354829 |
| 1426341_at | H1f0 | 1.635609 |
| 1424967_x_at | Plk4 | 1.6358463 |
| 1423358_at | Lrfn3 | 1.6361982 |
| 1460241_a_at | LOC100048338 | 1.6364613 |
| 1421237_at | Dtna | 1.6368943 |
| 1440770_at | --- | 1.6371928 |
| 1419223_a_at | Tuft1 | 1.637465 |
| 1455888_at | LOC100047619 | 1.6383226 |
| 1426300_at | Hsp90ab1 | 1.6386261 |
| 1421816_at | Ociad2 | 1.6395558 |
| 1439036_a_at | AI426953 | 1.6397887 |
| 1451298_at | Gabre | 1.6398803 |
| 1460207_s_at | Cryl1 | 1.6407925 |
| 1456748_a_at | Npy1r | 1.64188 |
| 1457878_at | Ncam1 | 1.6426641 |
| 1432360_a_at | Msh6 | 1.642747 |
| 1417030_at | Stau2 | 1.6428303 |
| 1421471_at | Rab6b | 1.6429143 |
| 1438740_at | Gemin4 | 1.64458 |
| 1417419_at | 1700010I14Rik | 1.6453538 |
| 1422592_at | --- | 1.645473 |
| 1458385_at | Narg1 | 1.6460195 |
| 1439758_at | Phlpp | 1.6462562 |
| 1449555_a_at | 2410018C20Rik | 1.6469992 |
| 1420514_at | --- | 1.647374 |
| 1457326_at | Itpr2 | 1.6476742 |
| 1453598_at | Josd3 | 1.6484641 |
| 1425581_s_at | Ankrd13c | 1.648758 |
| 1432229_a_at | Dcun1d1 | 1.6488329 |
| 1436414_at | Mthfsd | 1.6492625 |
| 1425099_a_at | Ankrd37 | 1.6498275 |
| 1435303_at | Tnik | 1.6499752 |
| 1435379_at | Tpd52 | 1.6517521 |
| 1424156_at | Sorbs1 | 1.6519569 |
| 1439627_at | 2810407C02Rik | 1.6522505 |
| 1422513_at | Med14 | 1.652508 |
| 1452717_at | Mlstd2 | 1.654363 |
| 1457898_at | Gdpd1 | 1.6548043 |
| 1425052_at | Prss2 | 1.6548449 |
| 1430700_a_at | Mlstd2 | 1.6555982 |
| 1437820_at | Cryl1 | 1.6556463 |
| 1455368_at | Kirrel3 | 1.6569632 |
| 1450440_at | Itga3 | 1.6573805 |
| 1449481_at | Fancl | 1.658209 |
| 1429054_at | Acta1 | 1.658374 |
| 1433496_at | Cd109 | 1.6590878 |
| 1416748_a_at | Fbxo2 | 1.6592313 |
| 1420344_x_at | --- | 1.6595224 |
| 1455735_at | Col8a1 | 1.660162 |
| 1452192_at | Pcdhb16 | 1.6604583 |
| 1446612_at | Tcea1 | 1.6608611 |
| 1437314_a_at | Birc1f | 1.6608909 |
| 1416670_at | --- | 1.661148 |
| 1429764_at | 2600010E01Rik | 1.6612067 |
| 1436558_at | --- | 1.6612216 |
| 1434016_at | Rbm39 | 1.6632907 |
| 1433487_at | Josd3 | 1.6633208 |
| 1418761_at | Lamp3 | 1.6635824 |
| 1433495_at | Kcnq5 | 1.6640601 |
| 1451351_at | Afp | 1.6646036 |
| 1453223_s_at | LOC100044162 | 1.6649314 |
| 1427483_at | Itpr2 | 1.6649466 |
| 1437700_at | 9930104L06Rik | 1.6655066 |
| 1436393_a_at | Sgms1 | 1.665729 |
| 1435750_at | AI451617 | 1.6663276 |
| 1438641_x_at | Lhx6 | 1.6667256 |
| 1428349_s_at | Cep68 | 1.6667631 |
| 1453187_at | Arhgef9 | 1.6685002 |
| 1420361_at | LOC630539 | 1.6688935 |
| 1436178_at | Gadd45gip1 | 1.6690512 |
| 1460408_at | A430110A21Rik | 1.669457 |
| 1416442_at | LOC100046468 | 1.6695806 |
| 1435703_at | Ada | 1.6701332 |
| 1435841_s_at | E130303B06Rik | 1.6703513 |
| 1429759_at | 4933433H22Rik | 1.6705883 |
| 1440822_x_at | 2610318N02Rik | 1.6708031 |
| 1430239_at | Smc4 | 1.6708343 |
| 1421668_x_at | Pcgf6 | 1.6712979 |
| 1457918_at | Rbl1 | 1.6713516 |
| 1426619_at | --- | 1.672118 |
| 1448320_at | Bcl2l15 | 1.6725192 |
| 1432287_a_at | LOC677224 | 1.672661 |
| 1438428_at | Rpp40 | 1.672684 |
| 1418471_at | --- | 1.67292 |
| 1428719_at | Gper | 1.673905 |
| 1416959_at | Dtna | 1.6746316 |
| 1455057_at | Mex3b | 1.6746446 |
| 1449244_at | Afp | 1.6765039 |
| 1448574_at | Thop1 | 1.6778791 |
| 1420928_at | 4933412E12Rik | 1.6779875 |
| 1424683_at | Dagla | 1.6789362 |
| 1435497_at | Mre11a | 1.6804478 |
| 1448907_at | Rwdd3 | 1.6807235 |
| 1456387_at | Ncoa1 | 1.6811558 |
| 1449154_at | Rrs1 | 1.6814919 |
| 1429106_at | Gata6 | 1.681979 |
| 1427004_at | Agpat9 | 1.6827843 |
| 1424148_a_at | Chic1 | 1.6833832 |
| 1421007_at | Olfml2b | 1.68359 |
| 1451356_at | Lphn3 | 1.684805 |
| 1448192_s_at | --- | 1.6858829 |
| 1452115_a_at | Prps1 | 1.6861008 |
| 1437709_x_at | Mtap1b | 1.6867927 |
| 1423801_a_at | Lck | 1.6881922 |
| 1453163_at | Nudt17 | 1.6882002 |
| 1454889_x_at | --- | 1.6891127 |
| 1428377_at | Grwd1 | 1.6894326 |
| 1445521_at | --- | 1.6895922 |
| 1418277_at | Tmem206 | 1.6903346 |
| 1435155_at | Syngr1 | 1.6930794 |
| 1452995_at | Rrp12 | 1.6942922 |
| 1417751_at | Ttc13 | 1.6946868 |
| 1453021_at | E2f2 | 1.6947346 |
| 1428389_s_at | Gemin5 | 1.6958761 |
| 1451306_at | 2010317E24Rik | 1.6961021 |
| 1417823_at | Pawr | 1.6973385 |
| 1421201_a_at | Pcdh10 | 1.6975176 |
| 1416173_at | LOC627626 | 1.6976075 |
| 1425475_at | Csmd3 | 1.6976256 |
| 1427227_at | Csf1 | 1.698091 |
| 1441836_x_at | Slc4a7 | 1.6991271 |
| 1449204_at | --- | 1.6994222 |
| 1448230_at | --- | 1.699553 |
| 1455750_at | 1700003F12Rik | 1.7001522 |
| 1423328_at | Sox11 | 1.7002861 |
| 1454036_a_at | Ddx27 | 1.7007825 |
| 1438307_at | --- | 1.7010913 |
| 1446179_at | Pgm2l1 | 1.7019246 |
| 1445874_at | Prkci | 1.7021927 |
| 1455799_at | 2410076I21Rik | 1.7026545 |
| 1430640_a_at | Ipo7 | 1.7043124 |
| 1417300_at | Rab6b | 1.7049364 |
| 1448194_a_at | 6720463M24Rik | 1.7050573 |
| 1448465_at | LOC671878 | 1.7050914 |
| 1441482_at | Otud4 | 1.707056 |
| 1457367_at | Rrad | 1.7084973 |
| 1444693_at | Gas5 | 1.7094917 |
| 1450193_at | 2010309G21Rik | 1.7101402 |
| 1436627_at | Nktr | 1.7107328 |
| 1433573_x_at | 1110012J17Rik | 1.7109047 |
| 1456597_at | Pgm2l1 | 1.7123473 |
| 1436577_at | Ctgf | 1.7139575 |
| 1428092_at | Slc1a3 | 1.714259 |
| 1452614_at | Schip1 | 1.7143242 |
| 1439553_s_at | Cd93 | 1.7148316 |
| 1424150_at | Atp11c | 1.7151197 |
| 1460441_at | Wdr43 | 1.7164155 |
| 1426569_a_at | Hells | 1.7179049 |
| 1417656_at | Brunol4 | 1.7194645 |
| 1430786_at | Hoxa7 | 1.7195253 |
| 1419483_at | Chd1l | 1.7196516 |
| 1437332_at | Ipo7 | 1.720756 |
| 1438672_at | Tmem206 | 1.7211959 |
| 1454737_at | Dock8 | 1.7215377 |
| 1456854_at | Ak5 | 1.7219973 |
| 1454102_at | Onecut2 | 1.7224712 |
| 1443095_at | Tpd52 | 1.7228884 |
| 1416689_at | LOC72520 | 1.7242064 |
| 1457145_at | Sfrs7 | 1.7257093 |
| 1419310_s_at | Slc25a30 | 1.7292895 |
| 1428471_at | LOC100046959 | 1.7304565 |
| 1440633_at | Gm944 | 1.7309318 |
| 1448031_at | 2810433K01Rik | 1.7313704 |
| 1423232_at | Gdpd5 | 1.7323583 |
| 1417333_at | Stap2 | 1.7349967 |
| 1424300_at | Fanca | 1.7352884 |
| 1418780_at | Hells | 1.7356707 |
| 1460448_s_at | Slc25a13 | 1.7400928 |
| 1436469_at | Tmem206 | 1.7404277 |
| 1431054_at | Ovgp1 | 1.7413148 |
| 1450562_at | --- | 1.7440362 |
| 1457687_at | Hic1 | 1.7444721 |
| 1417156_at | Cd2ap | 1.7452222 |
| 1435892_at | 5031425E22Rik | 1.7452667 |
| 1441692_at | Nat1 | 1.7456014 |
| 1440005_at | Ehd4 | 1.7473316 |
| 1439510_at | Ttc14 | 1.7473639 |
| 1430128_a_at | 2810451A06Rik | 1.7473938 |
| 1425273_s_at | --- | 1.7474891 |
| 1456046_at | D12Ertd553e | 1.7483793 |
| 1425826_a_at | 3110082D06Rik | 1.7487037 |
| 1422428_at | LOC100048391 | 1.7496446 |
| 1416779_at | Neurl | 1.7500658 |
| 1453727_at | 4930420K17Rik | 1.7500721 |
| 1424341_s_at | Adamtsl5 | 1.7502747 |
| 1424092_at | A230067G21Rik | 1.7510283 |
| 1419742_at | Atp11c | 1.751121 |
| 1452469_a_at | 1700037H04Rik | 1.7515826 |
| 1433465_a_at | Dtl | 1.7521319 |
| 1432188_s_at | Nol11 | 1.7522051 |
| 1441400_at | Ece2 | 1.7528427 |
| 1436592_at | Smc4 | 1.7530321 |
| 1451782_a_at | Pes1 | 1.753471 |
| 1416126_at | D5Ertd798e | 1.7541587 |
| 1436731_at | --- | 1.7548505 |
| 1437637_at | Ptpre | 1.7552158 |
| 1416693_at | Abcc4 | 1.7558583 |
| 1433839_at | Brunol4 | 1.7561074 |
| 1455453_at | E2f8 | 1.7566411 |
| 1446286_at | Sorbs1 | 1.7571832 |
| 1420518_a_at | Slc5a3 | 1.7596018 |
| 1452707_at | Vps35 | 1.7606962 |
| 1421990_at | Hs3st1 | 1.7621669 |
| 1431116_at | Rab3ip | 1.7626228 |
| 1424328_s_at | Chml | 1.7651963 |
| 1429089_s_at | Glt25d1 | 1.765689 |
| 1451117_a_at | Dtna | 1.7659475 |
| 1428909_at | Ccna2 | 1.7661288 |
| 1451684_a_at | 5830474E16Rik | 1.7666416 |
| 1460444_at | LOC100047579 | 1.7667757 |
| 1423430_at | Pcdhb5 | 1.7668581 |
| 1446150_at | Gimap6 | 1.7680448 |
| 1429053_at | Slamf9 | 1.7684888 |
| 1424463_at | Ndn | 1.7689066 |
| 1424935_at | Slc29a1 | 1.7689553 |
| 1458676_at | Hao1 | 1.769076 |
| 1429308_at | Ide | 1.770022 |
| 1454659_at | Dtd1 | 1.7706665 |
| 1420545_a_at | Ndn | 1.7706983 |
| 1450263_at | Slfn9 | 1.7717893 |
| 1425396_a_at | AI451617 | 1.7723875 |
| 1452405_x_at | Brunol4 | 1.7730448 |
| 1452305_s_at | Brcc3 | 1.7753481 |
| 1437796_at | Efemp1 | 1.7769693 |
| 1433271_at | Dis3 | 1.777107 |
| 1426371_at | Fert2 | 1.7774849 |
| 1439556_at | Tbc1d16 | 1.7776653 |
| 1418350_at | Cpeb2 | 1.7805033 |
| 1427002_s_at | Epha2 | 1.7810348 |
| 1423429_at | Klf12 | 1.7821125 |
| 1435665_at | Cd2ap | 1.783076 |
| 1421979_at | --- | 1.7833724 |
| 1420773_at | Onecut2 | 1.786049 |
| 1452202_at | Lama2 | 1.786778 |
| 1418441_at | Nsbp1 | 1.7885443 |
| 1449098_a_at | Rnpc3 | 1.7894176 |
| 1459216_at | Pik3cb | 1.7903544 |
| 1452958_at | AU021838 | 1.7943122 |
| 1421883_at | Arrb1 | 1.7949276 |
| 1438015_at | Tns4 | 1.7957864 |
| 1442019_at | Baiap2l1 | 1.7971511 |
| 1429149_at | Pnpo | 1.7977002 |
| 1460467_at | Mreg | 1.7986069 |
| 1422082_a_at | St3gal5 | 1.7996699 |
| 1453726_s_at | Notch4 | 1.8003209 |
| 1452811_at | 1500016O10Rik | 1.8003649 |
| 1423010_at | Gins1 | 1.8030975 |
| 1446146_at | Mapk6 | 1.8042462 |
| 1426285_at | Sap30 | 1.8043027 |
| 1416645_a_at | Syt1 | 1.8050541 |
| 1450171_x_at | Ttc14 | 1.8066808 |
| 1448127_at | Mphosph6 | 1.807814 |
| 1429056_at | Ankle1 | 1.8080236 |
| 1415811_at | 5031425E22Rik | 1.8083042 |
| 1435554_at | Prx | 1.8090936 |
| 1422972_s_at | Krt80 | 1.8098891 |
| 1429820_at | --- | 1.820955 |
| 1441272_at | H2-DMb1 | 1.822221 |
| 1433622_at | Hbegf | 1.8230946 |
| 1449210_at | Cdh15 | 1.8231796 |
| 1435650_at | Dtna | 1.8248358 |
| 1425293_a_at | Zxdb | 1.8263434 |
| 1460063_at | Tns4 | 1.8285718 |
| 1442059_at | 1500016O10Rik | 1.8286991 |
| 1448885_at | BC048355 | 1.8300425 |
| 1449226_at | 8430419L09Rik | 1.8310146 |
| 1436538_at | LOC14210 | 1.8320513 |
| 1449580_s_at | Tmem47 | 1.8326089 |
| 1437486_at | Trim6 | 1.8329456 |
| 1429570_at | Pacrg | 1.8342997 |
| 1419073_at | A130040M12Rik | 1.8344718 |
| 1448552_s_at | --- | 1.8364424 |
| 1429199_s_at | 9530048O09Rik | 1.8374491 |
| 1418681_at | Gyk | 1.8405408 |
| 1438232_at | Ier2 | 1.8412766 |
| 1423938_at | AU040829 | 1.8434435 |
| 1451308_at | Krt7 | 1.8435761 |
| 1427441_a_at | Matr3 | 1.8444057 |
| 1420336_at | Ccbl2 | 1.8455368 |
| 1439284_at | Chmp4c | 1.8459249 |
| 1418852_at | 2600010E01Rik | 1.8471105 |
| 1452159_at | Phtf2 | 1.8478208 |
| 1448342_at | Il15 | 1.8510462 |
| 1418856_a_at | Casp3 | 1.8524486 |
| 1427009_at | Dock5 | 1.8524548 |
| 1428379_at | Mtap2 | 1.8533236 |
| 1424161_at | B230206F22Rik | 1.8555732 |
| 1433884_at | Dbc1 | 1.8566678 |
| 1434981_at | Asphd2 | 1.8604101 |
| 1428551_at | 1700037H04Rik | 1.8609463 |
| 1449848_at | Elavl2 | 1.8615929 |
| 1416795_at | Uhrf1 | 1.8616515 |
| 1435628_x_at | Eya1 | 1.8619075 |
| 1423314_s_at | Clcn3 | 1.8630043 |
| 1433902_at | Nol5a | 1.86443 |
| 1439034_at | LOC100046044 | 1.8659494 |
| 1448695_at | Prmt6 | 1.867643 |
| 1423479_at | Ndn | 1.869791 |
| 1451484_a_at | --- | 1.8739152 |
| 1452545_a_at | Prss22 | 1.8745204 |
| 1448126_at | --- | 1.875793 |
| 1435883_at | Nbea | 1.8764831 |
| 1422870_at | 4732474O15Rik | 1.8765348 |
| 1421792_s_at | Sfmbt2 | 1.8834492 |
| 1434469_at | 2810416A17Rik | 1.8859029 |
| 1426936_at | Emp2 | 1.8862418 |
| 1435021_at | Fut9 | 1.8867723 |
| 1430167_a_at | Brcc3 | 1.8899006 |
| 1449500_at | Igf2bp1 | 1.892855 |
| 1455841_s_at | Clcn3 | 1.8929222 |
| 1439548_at | Cdt1 | 1.8959537 |
| 1435537_at | Tbc1d4 | 1.8963015 |
| 1443368_at | Traip | 1.8972183 |
| 1421493_a_at | 2900026A02Rik | 1.897899 |
| 1432408_a_at | Gas7 | 1.8989506 |
| 1434025_at | Calr3 | 1.9009095 |
| 1454799_at | --- | 1.9024687 |
| 1446001_at | Cacnb2 | 1.903043 |
| 1425166_at | --- | 1.9035676 |
| 1455289_at | 9330118A15Rik | 1.9043568 |
| 1458509_at | Cdh2 | 1.9053589 |
| 1456660_a_at | Sec1 | 1.9065024 |
| 1453070_at | Aqp1 | 1.9096595 |
| 1431039_at | 9330118A15Rik | 1.9116579 |
| 1453370_at | Ttc9 | 1.912497 |
| 1424577_at | St6gal1 | 1.912898 |
| 1433056_at | Ndn | 1.9130855 |
| 1448016_at | 5430421F17Rik | 1.9153142 |
| 1418253_a_at | Gdap1l1 | 1.9154217 |
| 1458585_at | Kcnn4 | 1.9166256 |
| 1437553_at | Mipol1 | 1.9169685 |
| 1436871_at | Zfp52 | 1.9169708 |
| 1449331_a_at | Cx3cl1 | 1.9173707 |
| 1449356_at | Mlstd2 | 1.9174072 |
| 1438444_at | Thy1 | 1.9187107 |
| 1427646_a_at | Sorbs1 | 1.9194282 |
| 1418687_at | Foxs1 | 1.9210062 |
| 1449419_at | Nefm | 1.9216052 |
| 1424207_at | Gmps | 1.92185 |
| 1420217_x_at | Sorbs1 | 1.9218589 |
| 1433536_at | Gprc5a | 1.9232845 |
| 1422060_at | Gata6 | 1.9247867 |
| 1423187_at | Rab3ip | 1.9261684 |
| 1451248_at | Ctsc | 1.929062 |
| 1455158_at | Wdr77 | 1.9297588 |
| 1433485_x_at | 1500005K14Rik | 1.9341133 |
| 1428652_at | LOC100044566 | 1.9347876 |
| 1443921_at | Gmps | 1.9362947 |
| 1435924_at | Fgd3 | 1.9365107 |
| 1444024_at | Ndst4 | 1.9368075 |
| 1440015_at | --- | 1.9369923 |
| 1417719_at | Tmem40 | 1.937584 |
| 1449975_a_at | Pde2a | 1.9397306 |
| 1427183_at | Bcl2 | 1.9401661 |
| 1434306_at | Nmnat2 | 1.9417026 |
| 1426165_a_at | 6720401G13Rik | 1.9419158 |
| 1456764_at | Pcdhb9 | 1.9420861 |
| 1418440_at | Arc | 1.9423175 |
| 1452247_at | Ndn | 1.9429591 |
| 1443534_at | Prmt7 | 1.9467837 |
| 1433789_at | Gmip | 1.9495311 |
| 1450064_at | AI467606 | 1.9505847 |
| 1452751_at | Pigf | 1.9519045 |
| 1425681_a_at | Rhox5 | 1.9565626 |
| 1426168_a_at | Gas7 | 1.9581044 |
| 1455256_at | Ebf3 | 1.9588642 |
| 1448961_at | Itpr3 | 1.9611667 |
| 1419186_a_at | Elavl1 | 1.9612457 |
| 1447112_s_at | Nuak2 | 1.962513 |
| 1457004_at | Eya1 | 1.9631362 |
| 1445837_at | LOC100044162 | 1.9662124 |
| 1452728_at | Otud4 | 1.9679574 |
| 1452665_at | Bcl2 | 1.9687129 |
| 1434850_at | Fanca | 1.9690953 |
| 1429747_at | Matr3 | 1.9744987 |
| 1430439_at | 0610010O12Rik | 1.9749831 |
| 1420836_at | BB557941 | 1.9762465 |
| 1453623_a_at | Ly6f | 1.9768002 |
| 1457658_x_at | Cd93 | 1.978877 |
| 1436825_a_at | Clcn3 | 1.984475 |
| 1456396_at | Rapsn | 1.9851078 |
| 1426340_at | Hbegf | 1.9863669 |
| 1420008_s_at | Slc1a3 | 1.9882963 |
| 1434296_at | LOC100046255 | 1.9893054 |
| 1430289_a_at | Rbm38 | 1.9901271 |
| 1452858_at | Lcorl | 1.9912744 |
| 1435641_at | Slc1a3 | 1.992506 |
| 1443117_at | F11r | 1.993187 |
| 1440311_at | 1700009N14Rik | 1.9939454 |
| 1434353_at | Igf2bp1 | 1.9941932 |
| 1429309_at | Pctk3 | 1.9955266 |
| 1421006_at | Sox11 | 1.9983678 |
| 1440335_at | Nipsnap1 | 1.9988085 |
| 1425184_at | Sgsm1 | 1.9989789 |
| 1433738_at | Gcat | 1.9993273 |
| 1439286_at | Cda | 2.0001166 |
| 1435292_at | --- | 2.0003872 |
| 1449541_x_at | Bok | 2.0056257 |
| 1441238_at | Glrp1 | 2.0073116 |
| 1430999_a_at | Elavl1 | 2.0082443 |
| 1421189_at | Atp10d | 2.0153677 |
| 1426471_at | Mybl2 | 2.0173042 |
| 1460262_a_at | Cdyl2 | 2.0176218 |
| 1429404_at | Snhg3 | 2.0181873 |
| 1455647_at | Ank | 2.0187066 |
| 1452364_at | Clcn3 | 2.0187333 |
| 1434068_s_at | --- | 2.022938 |
| 1429055_at | C030003D03Rik | 2.023758 |
| 1419609_at | LOC676024 | 2.0258522 |
| 1429943_at | Stap2 | 2.026949 |
| 1445414_at | Nkain2 | 2.0272005 |
| 1421851_at | Nos1 | 2.0272672 |
| 1457424_at | Col11a1 | 2.0372992 |
| 1437466_at | Prss2 | 2.0381007 |
| 1425175_at | 4631426J05Rik | 2.041364 |
| 1424076_at | Mctp1 | 2.0414238 |
| 1431894_at | LOC100039963 | 2.0439994 |
| 1435108_at | Grik2 | 2.044476 |
| 1417611_at | Rbm47 | 2.0477133 |
| 1432198_at | Gnao1 | 2.0483036 |
| 1429412_at | Prss2 | 2.0485086 |
| 1435374_at | Tmem74 | 2.048512 |
| 1416988_at | Camk1d | 2.0499744 |
| 1417859_at | Dyrk3 | 2.0519588 |
| 1419082_at | Spn | 2.0551388 |
| 1440870_at | BC029169 | 2.0580034 |
| 1418349_at | Tas1r2 | 2.059725 |
| 1426090_a_at | --- | 2.0621417 |
| 1418843_at | Tnfrsf22 | 2.0623064 |
| 1426647_at | Rps6ka6 | 2.0636313 |
| 1429161_at | LOC677224 | 2.0656111 |
| 1454806_at | Plac1 | 2.0656466 |
| 1439227_at | Rwdd3 | 2.066503 |
| 1440179_x_at | Gpr56 | 2.0676627 |
| 1429909_at | 6030490I01Rik | 2.0676851 |
| 1451780_at | 2210010L05Rik | 2.0692353 |
| 1452902_at | Tmem171 | 2.0700307 |
| 1457587_at | Ttc14 | 2.0701625 |
| 1438282_at | E2f8 | 2.070573 |
| 1452098_at | 1110012J17Rik | 2.0746036 |
| 1432130_a_at | Cdh2 | 2.0756943 |
| 1449544_a_at | Slc17a6 | 2.0802624 |
| 1442703_at | Epb4.1 | 2.0825632 |
| 1436311_at | H2-DMb1 | 2.085072 |
| 1425094_a_at | Slc25a24 | 2.085434 |
| 1422640_at | Bcl2 | 2.0892491 |
| 1416216_at | Mnd1 | 2.0909028 |
| 1417297_at | Tmeff2 | 2.0960386 |
| 1432276_at | Serpinb2 | 2.0996892 |
| 1442561_at | A030004J04Rik | 2.0999243 |
| 1435398_at | Klhl1 | 2.1019766 |
| 1429443_at | Etv4 | 2.1021078 |
| 1433110_at | Kcnh2 | 2.1042202 |
| 1450742_at | Poli | 2.1045005 |
| 1460044_at | Obsl1 | 2.105228 |
| 1460377_a_at | --- | 2.1070986 |
| 1434253_s_at | Des | 2.1089687 |
| 1418261_at | --- | 2.112587 |
| 1420339_at | Ccl17 | 2.1206257 |
| 1422703_at | Fetub | 2.1249092 |
| 1428653_x_at | Cxcl11 | 2.1254308 |
| 1448947_at | Prss2 | 2.1258264 |
| 1422444_at | Otud4 | 2.132572 |
| 1420707_a_at | Bzrap1 | 2.1393545 |
| 1456319_at | Tdrkh | 2.153584 |
| 1454686_at | Hck | 2.154467 |
| 1430423_s_at | LOC100044161 | 2.172774 |
| 1426731_at | --- | 2.1753204 |
| 1415945_at | Syn1 | 2.1784956 |
| 1425154_a_at | --- | 2.1843438 |
| 1443158_at | Rgs20 | 2.187383 |
| 1443247_at | Pla1a | 2.1882 |
| 1424077_at | A130082M07Rik | 2.1900709 |
| 1424232_a_at | Sdpr | 2.191801 |
| 1437079_at | Gabrb3 | 2.1930141 |
| 1421151_a_at | Onecut2 | 2.1995313 |
| 1451631_at | --- | 2.2003357 |
| 1445678_at | Naip2 | 2.200557 |
| 1440745_at | Csf1 | 2.2053993 |
| 1417619_at | Hspa4l | 2.2090445 |
| 1432179_x_at | C1ql3 | 2.2102334 |
| 1429422_at | Kcnn4 | 2.2259564 |
| 1437461_s_at | Pcdh10 | 2.2306283 |
| 1429475_at | Hoxd9 | 2.230844 |
| 1431197_at | Prkaa2 | 2.2410913 |
| 1421134_at | 4930519F16Rik | 2.2455838 |
| 1457044_at | Dusp4 | 2.2513473 |
| 1452079_s_at | Asxl3 | 2.2534494 |
| 1439557_s_at | Itga3 | 2.253512 |
| 1447778_x_at | Egr3 | 2.255514 |
| 1445687_at | Galnt13 | 2.2602012 |
| 1455803_at | Tnik | 2.2620602 |
| 1428823_at | Gabrb3 | 2.2682974 |
| 1451040_at | Ptpn22 | 2.2757137 |
| 1431725_at | Evi2a | 2.2791288 |
| 1418326_at | Tpd52l1 | 2.281524 |
| 1424398_at | 6330407I18Rik | 2.28317 |
| 1444790_at | Maob | 2.2915208 |
| 1431235_at | Ptpre | 2.2923536 |
| 1456020_at | Daam2 | 2.3021545 |
| 1417109_at | Spire2 | 2.3065808 |
| 1450065_at | Gfra1 | 2.3092282 |
| 1448896_at | Pawr | 2.3152385 |
| 1443832_s_at | Tmem37 | 2.3179843 |
| 1428519_at | Mlkl | 2.3180792 |
| 1416610_a_at | Alcam | 2.3245885 |
| 1456786_at | C030003D03Rik | 2.3339767 |
| 1416778_at | Foxp2 | 2.3464527 |
| 1429765_at | Gp49a | 2.3468704 |
| 1425893_a_at | D9Ertd720e | 2.3530555 |
| 1427878_at | Lphn3 | 2.3536673 |
| 1456767_at | Tmem74 | 2.362292 |
| 1424270_at | Tdrd7 | 2.3635402 |
| 1419553_a_at | Cpeb2 | 2.3930914 |
| 1441677_at | Adam8 | 2.3936129 |
| 1426930_at | Hapln4 | 2.3958807 |
| 1419257_at | Trem2 | 2.399803 |
| 1423281_at | Il2rg | 2.4009917 |
| 1425050_at | Galnt13 | 2.4016325 |
| 1430247_at | Galnt7 | 2.4114306 |
| 1427253_s_at | Nipsnap1 | 2.4183304 |
| 1437224_at | --- | 2.419787 |
| 1456665_at | Gyk | 2.431922 |
| 1423625_a_at | A130082M07Rik | 2.434476 |
| 1435763_at | Calr3 | 2.458338 |
| 1417682_a_at | Csf1 | 2.4657707 |
| 1421534_at | Ebf3 | 2.4678607 |
| 1452026_a_at | Rorb | 2.4679198 |
| 1429268_at | Grik2 | 2.4685862 |
| 1436304_at | Nkain2 | 2.4830468 |
| 1421153_at | AI662270 | 2.4949522 |
| 1425658_at | LOC100044161 | 2.4974878 |
| 1452411_at | AI662270 | 2.4989438 |
| 1455784_at | A630095E13Rik | 2.5001516 |
| 1456284_at | Ranbp3l | 2.502353 |
| 1438838_at | Dusp9 | 2.5050948 |
| 1457270_at | Prdm16 | 2.5065174 |
| 1449852_a_at | Chn1 | 2.5120957 |
| 1453232_at | Sdpr | 2.5227065 |
| 1421882_a_at | Ldb2 | 2.525879 |
| 1454913_at | Galnt7 | 2.5341048 |
| 1422681_at | Pgbd5 | 2.536122 |
| 1436394_at | Bicd1 | 2.5387142 |
| 1416517_at | 2010004A03Rik | 2.5609608 |
| 1448468_a_at | Gchfr | 2.5733802 |
| 1449559_at | Bcl2 | 2.5752552 |
| 1425750_a_at | C1ql3 | 2.5843046 |
| 1445106_at | Igsf9 | 2.5875864 |
| 1450379_at | Nes | 2.5924656 |
| 1450241_a_at | Grik2 | 2.5943592 |
| 1421945_a_at | Nol4 | 2.5977473 |
| 1449455_at | Cdyl2 | 2.6019163 |
| 1435382_at | Ebf3 | 2.6126854 |
| 1418355_at | Hspa4l | 2.6521025 |
| 1418027_at | 1110002E22Rik | 2.660856 |
| 1455448_at | 1810015C04Rik | 2.664426 |
| 1442819_at | Phex | 2.670306 |
| 1456494_a_at | --- | 2.6746876 |
| 1431925_at | Gnao1 | 2.67678 |
| 1429719_at | Als2cr12 | 2.677581 |
| 1422039_at | Bicd1 | 2.6889808 |
| 1423620_at | Loxl4 | 2.6893642 |
| 1437828_s_at | C230078M08Rik | 2.699985 |
| 1426839_at | --- | 2.7042 |
| 1415923_at | Kcnab1 | 2.7131863 |
| 1460576_at | LOC545086 | 2.724102 |
| 1460666_a_at | Plekhg4 | 2.7258034 |
| 1424951_at | Ptk2b | 2.7290418 |
| 1452513_a_at | Hspa4l | 2.7366118 |
| 1426575_at | Klhl32 | 2.7401848 |
| 1448472_at | Mgst2 | 2.7483904 |
| 1421732_at | Foxc2 | 2.7574365 |
| 1455707_at | Nol4 | 2.7680123 |
| 1441559_at | Trem3 | 2.768208 |
| 1418594_a_at | Gm885 | 2.7701662 |
| 1452190_at | Pkia | 2.7715552 |
| 1437756_at | Ctnnd2 | 2.778691 |
| 1449855_s_at | A130082M07Rik | 2.8172183 |
| 1457726_at | Slc11a1 | 2.8172386 |
| 1451010_at | Sdpr | 2.8198934 |
| 1437250_at | Slc35f3 | 2.8250983 |
| 1424834_s_at | Gabrg1 | 2.8263807 |
| 1420970_at | BC005512 | 2.8273308 |
| 1443837_x_at | Slco4a1 | 2.8306253 |
| 1421886_at | Serpinb7 | 2.8366323 |
| 1424595_at | Adcy7 | 2.8405032 |
| 1417318_at | Gdap1 | 2.8412702 |
| 1429203_at | Pcdh20 | 2.8416026 |
| 1449146_at | A130040M12Rik | 2.8460066 |
| 1436413_at | Brcc3 | 2.8479803 |
| 1451620_at | Popdc3 | 2.8525777 |
| 1420352_at | Parvb | 2.8585315 |
| 1442744_at | BC005512 | 2.8600557 |
| 1422842_at | Loxl4 | 2.8688853 |
| 1424223_at | Igfbp5 | 2.8811345 |
| 1455083_at | 4921509J17Rik | 2.8817067 |
| 1427573_at | Nap1l2 | 2.8934422 |
| 1460570_at | C3ar1 | 2.905741 |
| 1417995_at | Adcy7 | 2.9145198 |
| 1456858_at | --- | 2.9146385 |
| 1421629_at | Atp1b1 | 2.9174032 |
| 1427891_at | Syt1 | 2.9193976 |
| 1435361_at | Galnt7 | 2.9261103 |
| 1446720_at | Dusp9 | 2.928026 |
| 1426301_at | Zic1 | 2.9741068 |
| 1419599_s_at | Ngef | 2.9745398 |
| 1418739_at | Rhbdl2 | 2.9758735 |
| 1431402_at | Ldb2 | 2.9777606 |
| 1452496_at | Adcy7 | 2.9838336 |
| 1448145_at | Aim2 | 2.9895911 |
| 1452521_a_at | Klhl30 | 3.005814 |
| 1423856_at | C1ql3 | 3.0136316 |
| 1423714_at | Cadm1 | 3.0167918 |
| 1430362_at | Atp1b1 | 3.0239084 |
| 1423450_a_at | Rorb | 3.0595562 |
| 1445390_at | --- | 3.0710766 |
| 1451021_a_at | Bicd1 | 3.074394 |
| 1434272_at | Btbd11 | 3.0752323 |
| 1419168_at | 1810015C04Rik | 3.1198673 |
| 1418369_at | Calr3 | 3.1245193 |
| 1458518_at | --- | 3.1274223 |
| 1456069_at | Nppb | 3.1322484 |
| 1451322_at | Iqcg | 3.1436439 |
| 1422948_s_at | --- | 3.1877627 |
| 1435507_x_at | Gzme | 3.2039232 |
| 1428784_at | Naalad2 | 3.2185223 |
| 1425176_at | Kcnab1 | 3.2206252 |
| 1443160_at | Krt19 | 3.2213416 |
| 1442082_at | Mapk10 | 3.221476 |
| 1424701_at | Slco4a1 | 3.2262478 |
| 1435452_at | Btbd11 | 3.2446008 |
| 1435640_x_at | Akap3 | 3.2593808 |
| 1436805_at | Tnik | 3.2758846 |
| 1460575_at | Gzme | 3.3012202 |
| 1425576_at | Ap1s3 | 3.3085542 |
| 1424448_at | Atp1b1 | 3.3101654 |
| 1449415_at | Ankrd1 | 3.3145757 |
| 1423615_at | Itga6 | 3.323973 |
| 1449499_at | Lrp11 | 3.3290596 |
| 1424229_at | Itga6 | 3.329351 |
| 1450995_at | 3830403N18Rik | 3.335929 |
| 1426065_a_at | Blnk | 3.3711083 |
| 1419493_a_at | Atp1b1 | 3.3760324 |
| 1438483_at | Ar | 3.379033 |
| 1420882_a_at | Cadm1 | 3.379765 |
| 1418157_at | Rorb | 3.4063227 |
| 1426113_x_at | Ldb2 | 3.4305701 |
| 1426127_x_at | --- | 3.4329357 |
| 1450313_at | Mtap2 | 3.4371057 |
| 1417444_at | Alcam | 3.442701 |
| 1419413_at | Alcam | 3.4900162 |
| 1424292_at | Sntg1 | 3.4907625 |
| 1436472_at | Prdm16 | 3.5053065 |
| 1455238_at | Pkia | 3.5375435 |
| 1435078_at | Tmcc3 | 3.5576775 |
| 1451546_s_at | Msln | 3.5707462 |
| 1421400_at | Pla2g7 | 3.6159427 |
| 1433459_x_at | Alcam | 3.625871 |
| 1429478_at | Tmcc3 | 3.6319816 |
| 1435926_at | Ranbp3l | 3.6639457 |
| 1451273_x_at | Alcam | 3.7041843 |
| 1435656_at | Tmcc3 | 3.7051017 |
| 1416295_a_at | --- | 3.7416525 |
| 1456478_at | Tmcc3 | 3.8614745 |
| 1449010_at | C3ar1 | 3.891849 |
| 1416155_at | Ankrd1 | 3.9029145 |
| 1421402_at | Tmcc3 | 3.912788 |
| 1437199_at | Chrna1 | 3.9590511 |
| 1450791_at | Ar | 3.9673424 |
| 1433272_at | Evi1 | 3.976786 |
| 1444061_at | Cyp2c55 | 3.9833002 |
| 1434147_at | 1500005K14Rik | 4.0344315 |
| 1423060_at | 1110002E22Rik | 4.0531836 |
| 1420757_at | Tnnt2 | 4.0957117 |
| 1416365_at | Mapk10 | 4.1558313 |
| 1417212_at | Rgs20 | 4.183701 |
| 1451658_a_at | Gatm | 4.1986856 |
| 1439847_s_at | Myf5 | 4.2362924 |
| 1452839_at | Chrnb1 | 4.247778 |
| 1439129_at | B830045N13Rik | 4.3239074 |
| 1460713_at | Eya1 | 4.3706894 |
| 1442680_at | Elavl2 | 4.417767 |
| 1452430_s_at | 1500005K14Rik | 4.471683 |
| 1448978_at | Dub1 | 4.561548 |
| 1422520_at | 2010300F17Rik | 4.5656776 |
| 1438160_x_at | --- | 4.5718765 |
| 1460330_at | Gimap9 | 4.5977383 |
| 1436879_x_at | Gpr149 | 4.6188507 |
| 1434468_at | Tnnt2 | 4.6727033 |
| 1418274_at | C3ar1 | 4.7721076 |
| 1435302_at | Gzmd | 4.7745833 |
| 1431178_at | Prdm16 | 4.8401403 |
| 1439272_at | Gpr149 | 4.8895793 |
| 1450229_at | Elavl2 | 4.9008994 |
| 1417976_at | H19 | 5.1205826 |
| 1434766_at | Mcpt8 | 5.1273246 |
| 1416774_at | Cd300lb | 5.1860003 |
| 1434515_at | Gzmd | 5.360803 |
| 1424081_at | LOC100044533 | 5.7150607 |
| 1426337_a_at | Pet2 | 5.936092 |
| 1416203_at | Prdm16 | 5.9880753 |
| 1427261_at | Dppa2 | 6.053003 |
| 1452402_at | Gabrb3 | 6.2198505 |
| 1430986_at | Gabrg1 | 8.294321 |
| 1457194_at | Dppa2 | 8.59135 |
| 1421101_a_at | Asb5 | 9.041353 |
| 1452476_at | Prnd | 9.25437 |
| 1453683_a_at | Pcdh20 | 9.580755 |
